# Cumulative solar ultraviolet radiation exposure and basal cell carcinoma of the skin in a nationwide US cohort using satellite and ground-based measures

**DOI:** 10.1186/s12940-019-0536-9

**Published:** 2019-12-27

**Authors:** Mark P. Little, Martha S. Linet, Michael G. Kimlin, Terrence Lee, Zaria Tatalovich, Alice J. Sigurdson, Elizabeth K. Cahoon

**Affiliations:** 1 Radiation Epidemiology Branch, Division of Cancer Epidemiology and Genetics, National Cancer Institute, NIH, DHHS, 9609 Medical Center Drive, Bethesda, MD 20892-9778 USA; 2 NHMRC Centre for Research Excellence in Sun and Health, University of the Sunshine Coast, Brisbane, Queensland 4556 Australia; 3 Cancer Council Queensland, Brisbane, Queensland Australia; 4 Surveillance Research Program, Division of Cancer Control and Population Sciences, National Cancer Institute, NIH, DHHS, Bethesda, MD 20892-9778 USA

**Keywords:** Non-ionizing radiation, Ultraviolet solar radiation, Basal cell carcinoma of the skin, Radiologic technologist

## Abstract

**Background:**

Basal cell carcinoma of the skin (BCC) is the most common cancer in populations of European ancestry. Although consistently linked with basal cell carcinoma of the skin in case-control studies, few prospective cohort studies have evaluated the shape of the exposure-response of basal cell carcinoma associated with cumulative radiant solar ultraviolet exposure (UVR).

**Methods:**

We followed 63,912 white cancer-free US radiologic technologists from entry (1983–1998) to exit (2003–2005) with known ultraviolet irradiance at up to 5 residential locations. Using generalized-additive and relative risk models we analyzed the exposure-response of basal cell carcinomas associated with ambient cumulative ultraviolet radiant exposure using ground-based National Solar Radiation database Average Daily Total Global data and satellite-based National Aeronautics and Space Administration Total Ozone Mapping Spectrometer data.

**Results:**

There were 2151 technologists with an incident primary basal cell carcinoma. Risk of basal cell carcinoma rose with increasing cumulative ultraviolet radiation exposure using both measures, such that 1 MJ cm^− 2^ increased basal cell carcinoma risk by 8.48 (95% CI 5.22, 11.09, *p* < 0.001) and by 10.15 (95% CI 6.67, 13.10, *p* < 0.001) per 10,000 persons per year using the Average Daily Total Global and Total Ozone Mapping Spectrometer ultraviolet data, respectively; relative risk was likewise elevated. There was some evidence of upward curvature in the cumulative ultraviolet exposure response using both exposure measures with a greater increase in risk of basal cell carcinoma at higher levels of ultraviolet radiation exposure, but less evidence for curvature in relative risk. There are indications of substantial variation of relative risk with time after exposure and age at exposure, so that risk is highest for the period 10–14 years after ultraviolet radiation exposure and for those exposed under the age of 25.

**Conclusions:**

We observed increases in risk of basal cell carcinoma and a similar exposure-response for ground-based and satellite ultraviolet radiation measures. Our observations suggest that interventions should concentrate on persons with higher levels of ultraviolet radiation exposure.

## Background

Exposure to solar ultraviolet radiation (UVR), and specifically ultraviolet B (UVB) radiation, is the primary risk factor for basal cell carcinoma (BCC) [1], the most common cancer in populations of European ancestry. However, few studies have evaluated the relationship between cumulative UVR and BCC risk [2]. Information on BCC is usually not uniformly collected by many US or other population-based cancer registries so that quantitative information on risk in relation to many suspected etiologically relevant factors is scarce [3, 4].

In several cohorts [5–7], UVR has been assessed using Robertson-Berger (RB) ground-based meters over the lifetime. However, the accuracy of the RB meter values has been questioned [8]. The exposure metric used was limited to average UVR over a lifetime.

More recently, the AVerage daily total GLObal solar radiation (AVGLO) ground-based estimates [9] based on total solar radiation data from the National Solar Radiation Database (NSRAD) of the Department of Energy (DOE), were used to assess skin cancer risk in the large multi-center Women’s Health Initiative observational cohort [10] and melanoma in US Surveillance, Epidemiology and End Results population-based registry data [11]. In both studies the AVGLO estimates grouped participants into four exposure groups. National Aeronautics Space Administration (NASA) Total Ozone Mapping Spectrometer (TOMS) satellite-based UVR data has been used to study breast and thyroid cancer in the US radiologic technologists (USRT) [12, 13], and melanoma in residents of Seattle-Puget Sound [14] and in the Nurses’ Health Studies (NHS) [15]. In each of these studies, a single exposure metric, often average annual ambient UVR, was considered.

Using self-reported data on incidence (with substantial medical record confirmation) of BCC, in the present paper we conduct one of the first cohort analyses of BCC to assess absolute risks in relation to cumulative UVR radiant exposure, to study the shape of the UVR exposure-response with BCC using cumulative UVR exposure, and to compare AVGLO ground-based measures versus NASA TOMS satellite measures in quantifying absolute and relative risks and the shape of the UVR-BCC relationship using cumulative UVR exposures. The metrics are based on self-reported residential history of individual cohort members. We also examine BCC risk according to the age at which UVR exposure occurs, and the number of years from UVR exposure to the time at risk. The study is distinguished by its large size, prospective approach, and population residing at a wide range of latitudes.

## Materials and methods

### Overview of USRT study

The USRT cohort consists of 146,022 radiologic technologists living in the United States and its territories who were certified by the American Registry of Radiologic Technologists (ARRT) for at least 2 years between 1926 and 1982. Methods of the study have been previously described [16–19] and detailed information can be found online at www.radtechstudy.nci.nih.gov. Periodic surveys were undertaken between 1983 and 2005. For the first survey, questionnaires were sent during 1983–89 to 132,298 surviving cohort members with a postal address; 90,305 (68%) technologists completed the questionnaire. A second questionnaire was sent to 126,628 living and located members during 1994–1998; 90,972 (72%) technologists responded. If a technologist answered both the first and second questionnaire, the earlier of the two was designated as the baseline questionnaire. A third questionnaire was sent in 2003–2005 to 105,694 participants who had answered at least one previous questionnaire; of these, 73,838 (73%) responded.

### Study population and follow-up

In the current study we evaluated the 63,912 white technologists who completed a baseline (earliest of first or second) questionnaire and the third questionnaire and did not report any specific cancer (including BCC) or an unknown type of cancer diagnosed prior to their baseline questionnaire, and who had sufficiently complete residential history that they could be linked to two separate databases of UVR irradiance estimates (AVGLO, TOMS) by calendar time period and location. Non-white respondents were excluded due to the notably lower BCC incidence rates compared to whites, and the relatively small number of these technologists. Cohort members were followed from completion of their baseline questionnaire to the earlier of first primary cancer diagnosis, including BCC, or the third questionnaire (administrative censoring).

### BCC case ascertainment and medical validation

Case definition was self-reported incident first primary BCC (with year of diagnosis) among subjects who responded to the third questionnaire, and who had previously completed the first or second questionnaire (or both). Of the 1355 self-reported BCCs from the second questionnaire, medical records were obtained on approximately 50% [6]. Among those, 97% (668 BCCs) were confirmed. For the 4862 BCCs self-reported on the third questionnaire, medical records were obtained on 2058 (42.3%) and of these 1762 (85.6%) were confirmed as a BCC. Because of the high medical record confirmation, we defined both confirmed as well as self-reported BCCs that lacked medical record confirmation.

For those cohort members reporting a skin cancer on the third questionnaire we sent an additional questionnaire for the technologists to complete and note the anatomic site of the skin cancer on a pictograph of a human form. We instructed cohort members to report only those cancers that were confirmed by pathology review and not to report “pre-cancerous” skin lesions or lesions that were treated but never confirmed to be skin cancer. For purposes of the present paper we restrict all analyses to the 2151 BCC cases who responded to the supplementary questionnaire (Additional file 2).

### UVR exposure assessment

As discussed elsewhere [20] (and see also the Discussion) a biologically relevant solar UVR measure is one that includes ultraviolet A (UVA) and UVB. A standard UVR exposure metric is cumulative UVR radiant exposure (in units of joule (J) m^− 2^), which is proportional to cumulative solar UVR energy deposition on a surface over a period of time. This measure of cumulative solar ambient UVR exposure is recommended by the Commission Internationale de l’Eclairage (CIE) (International Commission on Illumination) [21]. We estimated this measure from self-reported history of lifetime residences of each eligible technologist in the USRT using two separate solar radiation measurement datasets, one ground based, AVGLO [9, 20], the other satellite-based, TOMS [22] as follows.

On the third questionnaire, residential location for each technologist for five age periods (age < 13, 13–19, 20–39, 40–64, 64+) was collected. The NSRAD database produced by the National Renewable Energy Laboratory (NREL) under the US DOE’s Resource Assessment Program is the largest ground-based solar measurement network in the US, containing statistical summaries computed from hourly measurement data (with some infilling for missing data) for 239 US radiation stations for the period 1961–1990, including monthly, yearly, and 30-year average global solar radiation measures. Tatalovich et al [9] incorporated AVGLO measures, including latitude, longitude, and elevation from a 30 arc-second Digital Elevation Model into the ANUSPLIN spline-interpolation algorithm to deliver estimates of potential solar ambient irradiance (~ 100–3000 nm) at 1 km^2^ resolution in the US. AVGLO used 30-year averages of ANUSPLIN spline-interpolated ground solar ambient irradiance measurements after initial analysis of temporal variability that showed no statistically significant difference between the three 10-year periods embedded in the 1961–1990 data summaries for each radiation station [9]. For the current study, these estimates were aggregated to zip-codes of historical residences of persons and linked to location of residence as determined from the third questionnaire in the USRT study cohort.

The TOMS database (http://toms.gsfc.nasa.gov), which is maintained by NASA, provides a daily estimate of ambient UVB exposure, measured as the irradiance at 305 nm in mW m^− 2^ nm^− 1^ [23], in a 1° longitude × 1° latitude grid. Using city and state of residence reported on the third questionnaire for the five age periods reported above, ambient UVR irradiance was calculated at each point over the lifetime of the individual from TOMS-estimated ambient irradiance. The scaling of both these measures to provide cumulative UVR radiant exposure (in units of MJ cm^-2^) used the method previously described [20]. At a latitude of 38.9°N (approximately corresponding to that of Washington DC) a person exposed from birth to age 50 years would have a cumulative UVR radiant exposure of ~ 1112 kJ cm^− 2^ assessed via AVGLO, or ~ 971 kJ cm^− 2^ assessed via TOMS.

### Statistical analysis

In order to model the absolute excess risk associated with UVR we fitted a generalized-additive model (GAM) [24], in which the number of cases in the stratum with person years *PY*, after cumulative UVR radiant exposure, *H*(*t*) (in J cm^− 2^), at age *t*, and with various other explanatory covariates, *Z* = (*Z*_*i*_) (in most cases a log-linear function of a fifth order polynomial in birth year, log[age] and baseline questionnaire number - see footnotes to Table 2, Additional file 1: Table S2), was assumed to be Poisson distributed with mean given by:

1 $$ PY\left[\lambda \left(t,\left({Z}_i\right)|\left({\phi}_i\right),\left({\beta}_i\right)\right)+\alpha H(t)\right] $$

The parameter *α* is the excess BCC risk per year of follow-up and per unit of cumulative radiant exposure, and the (*ϕ*_*i*_), (*β*_*i*_) are other parameters used to model the log-linear background rate via the function *λ*(*t*, (*Z*_*i*_)| (*ϕ*_*i*_), (*β*_*i*_)); for example, one commonly used form of this function is $\lambda \left(t,\left({Z}_i\right)|\left({\phi}_i\right),\left({\beta}_i\right)\right)=\exp \left[\sum \limits_{i=0}^2{\phi}_i\ln {\left[t\right]}^i+\sum \limits_i{\beta}_i{Z}_i\right]$. We also fitted similar Poisson linear-quadratic or exponential relative risk models. Models were additionally fitted allowing for separate contributions of the time-varying cumulative radiant UVR exposure to temporal windows defined by time since exposure or age at exposure (Table 3). So for example in follow-up of a given individual we accumulated cumulative radiant exposure under and over the age of 25, and at each point of follow-up the cumulative radiant exposure 5–9 years, 10–14 years and > 15 years before that point; the cumulative totals, varying over follow-up of the individual, were person-year weighted as with all the other model variables. Further details of these linear/linear-quadratic/exponential models in cumulative radiant UVR exposure are given in the Additional file 1: Supplementary Methods. Model fitting was performed using R [25] and Epicure [26]. Confidence intervals (CI) were estimated from the profile likelihood [27], or if this did not converge using Wald-based CI. For each model the Akaike information criterion (AIC) [28, 29] was estimated, a measure of goodness of fit that penalizes overfitted models and also facilitates comparison of goodness of fit of non-nested models. Extensive additional analysis, in a paper that is in preparation, did not suggest that any of the variables reported in Additional file 1: Table S1, or various additional variables (natural hair color, skin complexion, eye color, Gaelic ancestry, Hispanic ethnicity) appreciably confounded the dose response.

## Results

There were a total of 63,912 subjects in this analysis of the cohort with 1,082,775 person-years of follow-up, and 2151 cases of BCC reported (Table 1). At study entrance cumulative UVR radiant exposure had an estimated mean of 849.8 kJ cm^− 2^ (range of 438.5, 2266.0) using AVGLO, and an estimated mean of 724.0 kJ cm^− 2^ (range 192.6, 1968.3) using TOMS (data not shown).

**Table 1:** Distribution (%) of selected baseline characteristics of basal cell carcinoma cases and non-cases among 63,912 white participants in the US Radiologic Technologists study

Characteristic	No BCC	BCC
Person-years at risk		
1,082,775		
Age at entry, mean (SD)		
38.81 (9.41)		
Age at exit, mean (SD)		
55.75 (8.43)		
Total	61,761	2151
Sex		
Male	12,651 (20.5)	431 (20.0)
Female	49,110 (79.5)	1720 (80.0)
Birth year		
< 1940	9787 (15.8)	558 (25.9)
1940–1949	21,060 (34.1)	732 (34.0)
1950+	30,914 (50.1)	861 (40.0)
Highest level of education		
Unknown	9703 (15.7)	228 (10.6)
School	241 (0.4)	19 (0.9)
Vocational/other	2414 (3.9)	80 (3.7)
Two-year rad tech program	29,224 (47.3)	981 (45.6)
College or graduate school	20,179 (32.7)	843 (39.2)
Smoking status		
Unknown smoking status	141 (0.2)	4 (0.2)
Never smoker	30,606 (49.6)	1067 (49.6)
Former smoker	17,502 (28.3)	678 (31.5)
Current smoker	13,144 (21.3)	394 (18.3)
Smoked, unknown if current smoker	368 (0.6)	8 (0.4)
Alcohol (drinks/week)		
Unknown	910 (1.5)	18 (0.8)
Never drinker	10,632 (17.2)	342 (15.9)
0–2.99	34,751 (56.3)	1154 (53.6)
3–6.99	9051 (14.7)	370 (17.2)
≥7	6417 (10.4)	267 (12.4)

**Fig. 1:**
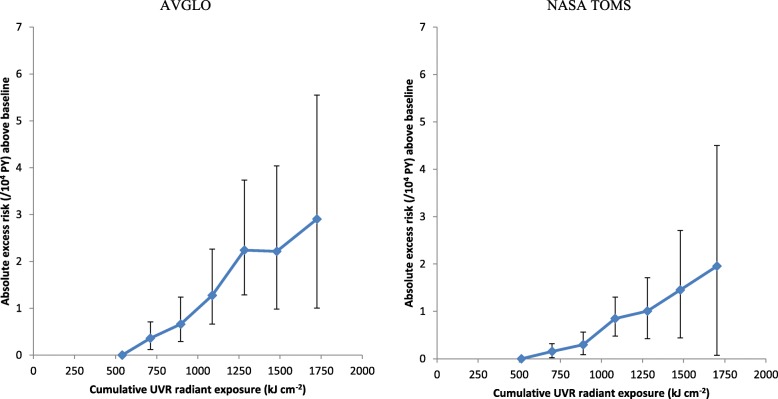
Absolute excess risk of basal cell carcinoma (+ 95% CI) over baseline level (defined by cumulative ultraviolet radiation (UVR) radiant exposure < 600 kJ m^−2^) in relation to cumulative ultraviolet radiant exposure, estimated using AVGLO or NASA TOMS data. Baseline model fitted to 63,963 white technologists adjusted for baseline questionnaire, ln[age], birth year, [birth year]^2^, [birth year]^3^, [birth year]^4^, [birth year]^5^. Intervals of UVR cumulative radiant exposure used are 0-599 (baseline), 600-799, 800-999, 1000-1199, 1200-1399, 1400-1599, 1600+ kJ cm^-2^

Table 2, Fig. 1 and Additional file 1: Table S4 demonstrate the strongly increasing risk of BCC with increasing cumulative UVR radiant exposure, so that using the AVGLO ground-based data a UVR cumulative radiant exposure of 1 MJ cm^− 2^ increases the estimated absolute risk of BCC by 8.48 (95% CI 5.22, 11.09, *p* < 0.001) per 10,000 persons per year; using the NASA TOMS satellite-based data, the estimated absolute risk was 10.15 (95% CI 6.67, 13.10, *p* < 0.001) per 1 MJ cm^− 2^ per 10,000 persons per year. Table 2, Fig. 1 and Additional file 1: Table S4 also demonstrate that there is significant (*p* < 0.002) upward curvature in the cumulative UVR radiant exposure response using either measure of UVR. The AVGLO and TOMS measures of UVR are highly correlated (Additional file 1: Figure S1). Nevertheless, the AIC statistics in Table 2 suggest that the linear-quadratic model using the AVGLO UVR data fitted slightly better than the analogous model fitted using the TOMS UVR measurements. Additional file 1: Table S2 demonstrates that there is no significant curvature in fits of the (linear) relative risk model to the AVGLO data. The analogous model fitted to the TOMS data did not converge, and so goodness of fit cannot be reliably assessed (Additional file 1: Table S2). In general linear models fit better than the log-linear model, as shown by the AIC statistics (Table 2, Additional file 1: Table S2).

**Table 2:** Curvature in exposure response of absolute risk of basal cell carcinoma (BCC) with ultraviolet radiation (UVR) cumulative radiant exposure, using AVGLO and NASA TOMS measures of UVR among 63,912 white technologists^c^

Model	Linear excess absolute risk per MJ/cm^2^ per 10^4^ person year (+ 95% CI)	Quadratic excess absolute risk per [MJ/cm^2^]^2^ per 10^4^ person year (+ 95% CI)	*p*-value	AIC
AVGLO				
Linear	8.48 (5.22, 11.1)		< 0.001^a^	11,926.4
Linear-quadratic	−0.11 (−5.17, 4.59)	13.06 (7.15, 18.9)	< 0.001^b^	11,910.2
NASA TOMS				
Linear	10.15 (6.67, 13.1)		< 0.001^a^	11,920.7
Linear-quadratic	3.34 (−2.91, 8.54)	10.1 (4.00, 16.3)	0.001^b^	11,912.1

^a^test for departure of UVR linear exposure-response from null

^b^test for departure of UVR linear-quadratic exposure-response from linearity

^c^All analysis used linear-quadratic model (S1’) with adjustment to the baseline BCC rate for baseline questionnaire, ln [age], birth year, [birth year]^2^, [birth year]^3^, [birth year]^4^, [birth year]^5^

Table 3 demonstrates that there is at best borderline significant variation of excess absolute risk by time since exposure (*p* = 0.279) or with age at exposure (*p* = 0.085); this contrasts with more pronounced and significant variation of relative risk both with time since exposure (*p* = 0.034), and with age at exposure (*p* = 0.012) with relative risk particularly large for the period 10–14 years after UVR exposure and for exposures under the age of 25 (Table 4). However, there are problems of parameter convergence using all models (Tables 3 and 4). Additional file 1: Table S3 demonstrates that there are no strong modifications of the effect of cumulative UVR radiant exposure effect by attained age, whether for absolute (*p* = 0.185) or relative risk (*p* = 0.089). There are no significant differences between the sexes in excess absolute risk (*p* = 0.152, results not shown), although there are stronger indications that the excess relative risk for males is higher (by about 25%) than for females (*p* = 0.008, results not shown).

**Table 3:** Excess absolute risk of basal cell carcinoma (BCC) in relation to AVGLO-derived cumulative ultraviolet radiation (UVR) radiant exposure in intervals of age at exposure and time since exposure^a^

Group	Excess absolute risk per cumulative UVR radiant exposure (MJ cm^− 2^) per 10^4^ person year	*p*-value^b^
Age at exposure (years)		
< 25	8.05^c^ (4.78, 11.1)	0.085^c^
≥ 25	19.4^c^ (8.37, 28.0)	
Time since exposure (years)^d^		
5–9	-36.9^e^ (-339.0^f^, 265.2^f^)	0.279^e^
10–14	50.7^e^ (-261.5^f^, 362.9^f^)	
≥ 15	11.8^e^ (-0.41^f^, 23.9^f^)	

^a^All analysis used models (S5) and (S7) with adjustment to the baseline BCC rate for baseline questionnaire, ln [age], ln [age]^2^, birth year, [birth year]^2^, [birth year]^3^, [birth year]^4^, [birth year]^5^

^b^*p*-value for heterogeneity by age at exposure/time since exposure

^c^weak indications of lack of convergence

^d^time since exposure is the difference in years between the age at which UVR exposure is determined and the age of the individual at a particular instant of follow-up, varying instantaneously with continuing follow-up for that individual

^e^indications of lack of convergence

^f^Wald-based confidence interval

**Table 4:** Excess relative risk of basal cell carcinoma (BCC) in relation to AVGLO-derived cumulative ultraviolet radiation (UVR) radiant exposure in intervals of age at exposure and time since exposure^a^

Group	Excess relative risk per UVR cumulative radiant exposure (10^6^ J cm^− 2^)	*p*-value^b^
Age at exposure (years)		
< 25	2.37^c^ (-4.11^d^, 8.85^d^)	0.012^c^
≥ 25	1.07^c^ (-3.54^d^, 5.68^d^)	
Time since exposure (years)^e^		
5–9	− 3.59^c^ (− 45.0^d^, 37.8^d^)	0.034^c^
10–14	7.11^c^ (-38.5^d^, 52.8^d^)	
≥ 15	1.58^c^ (-3.10^d^, 6.25^d^)	

^a^All analysis used models (S4) and (S6) with adjustment to the baseline BCC rate for baseline questionnaire, ln [age], ln [age]^2^, birth year, [birth year]^2^, [birth year]^3^, [birth year]^4^, [birth year]^5^

^b^*p*-value for heterogeneity by age at exposure/time since exposure

^c^indications of lack of convergence

^d^Wald-based confidence interval

^e^time since exposure is the difference in years between the age at which UVR exposure is determined and the age of the individual at a particular instant of follow-up, varying instantaneously with continuing follow-up for that individual

## Discussion

In this nationwide US study of radiologic technologists, we observed that there were highly significant increasing trends of BCC risk with increasing UVR cumulative radiant exposure, using two independent measures of UVR, one ground based and the other derived from satellite data. Both measures of UVR revealed similar excess absolute risk and demonstrated upward curvature in the UVR absolute risk exposure response, so that the excess absolute BCC risk per unit of cumulative UVR radiant exposure is greater for those with higher levels of UVR exposure (Table 2, Fig. 1); there is less strong evidence for curvature using the relative risk models (Additional file 1: Table S2), but there are problems of convergence with some of these models which render interpretation problematic. There is substantial and significant variation of relative risk with time after exposure and age at exposure, so that risk is highest for the period 10–14 years after UVR exposure and for those exposed under the age of 25; the problems of convergence with the relative risk model complicate the interpretation of this finding (Table 4). However, there is much less strong (and at best borderline significant) variation of excess absolute risk with time after exposure or age at exposure (Table 3).

Previous relevant literature on cumulative UVR and BCC risk is limited. Wei-Passanese et al. studied the correlation of multiple non-melanoma skin cancers (NMSC) per person with UVR index by state of residence at various ages (birth, age 15, age 30) in the US NHS [30]. The authors document an increase in risk of such multiple tumors with increasing level of the UV index [30]. The UVR index that Wei-Passanese et al. used was developed by the US National Weather Service and the US Environmental Protection Agency [31, 32], and correlates with UVR irradiance. However, as the index is based on the burning effect of UVR on human skin [31], it is not directly related to impact on BCC risk. Xiang et al. [33] performed a meta-analysis of age- and sex-specific incidence of NMSC in 40 published studies, correlating it with ambient UVR weighted to the action spectrum for sunburn [34] derived from NASA TOMS satellite data [35, 36]. Neither study estimated individual cumulative UVR radiant exposures.

The present study has several strengths. The study is of a nationwide US cohort of both sexes (75% female technologists), with residences located at a wide range of latitudes, with long term follow-up and lifetime residential history. Additionally, our study used two quantitative measurements for ambient UVR, ground-based and satellite-based, something that has, to our knowledge, only been done in one other study, a cross-sectional study of melanoma in a number of US states [37]. Uniquely, we assess risk of BCC in relation to residentially-determined cumulative radiant UVR exposure. As documented in the Methods, a large proportion of the BCCs were validated by medical records. Since BCC is not captured in most US cancer registries, there are few longitudinal studies with information on incident BCC, among them specialized occupational cohorts such as the US NHS [3] or the Nambour Skin Cancer Study in Queensland, Australia [4, 38]. The USRT cohort includes detailed lifestyle and occupational information, in particular on occupational radiation exposure. There were only weak indications that occupational radiation exposure modified BCC risk [18], and there were no indications of interaction with UVR. In addition to ambient UVR exposure, education, income, cigarette smoking, alcohol consumption, body mass index, hours exercise per week, eye color, skin complexion, ever sunburnt, number of blistering sunburns before age 15, skin reaction to strong sunlight and number of dental X rays were all significantly associated with BCC risk [18]. Interestingly, male radiologic technologists had slightly lower underlying BCC risk (by about 10%) than females, although there was little evidence of difference between the sexes in the UVR-associated absolute excess risk (*p* = 0.152, results not shown). This contrasts with results of some other studies, which show a rather higher risk of BCC for men than women [3]. A strength of this cohort is that the technologists worked largely indoors, so that work-related variations in UVR at a given location are unlikely.

Limitations of our study include the fact that BCC was self-reported, although a large proportion (> 85%) of those BCC for which medical records could be obtained were confirmed from these (see Methods). This makes under-ascertainment more likely than over-ascertainment. The members of the USRT cohort are medically trained and so would be expected to have heightened awareness and access to means of early detection of BCC [19], both factors making it unlikely that there would be substantial under-ascertainment either. However, the possible under- or over-ascertainment is unlikely to affect the shape of the dose-response strongly, because the exposure metric is not self-reported, but based on residential history. Our assessment of ambient UVR was based on self-reported residential history locations for five age periods and does not account for UVR exposures in non-residential locations such as during vacations or other residential location not recorded in these five age periods. However, for most technologists it would not be expected that non-residential location would contribute substantially to UVR exposure, nor would one expect that recall of residential location would be appreciably in error. If there were such errors they are likely be of classical form, and as such would tend to bias the trends of BCC with UVR towards the null [39]. We also do not take account of any behavioral modifications of UVR exposure, for example the number of hours spent outdoors. Although information of this sort was recorded in the questionnaires returned by the technologists, it was not done so prospectively, so that there is potential for recall bias in using it. The underlying cohort is unlikely to be representative of the general US population, not least because as with all occupational cohorts it is selected. The selection will likely entail that the technologists, at least while they remain in work, are fitter than the general population, and there may be differences in mortality and disease rates associated with being employed [40]. Cohort members had additionally to survive to answer the third questionnaire. However, this degree of selection will not necessarily bias our analysis, since everyone had to survive to answer this questionnaire, and BCC risk was assessed conditional on that. Follow-up was censored at the date of self-reported BCC or the third questionnaire. The plausible assumption was made that such censoring was uninformative with respect to BCC. There are other reason why this cohort is unlikely to be representative of the US population, specifically their somewhat higher socioeconomic status, with many of the technologists having attended or completed an associate or four-year college degree. In Scotland there are increasing trends of BCC risk with decreasing socioeconomic status [41], but the opposite pattern has been seen in Denmark and the Netherlands [42, 43]. To the best of our knowledge this has not been studied in the US. It is clear from a previous analysis of this data [20] that although the technologists moved a substantial distance over life, there is comparatively little movement over life in latitude. As such the UVR exposure an individual receives is likely to be a nearly constant level of radiant exposure. Therefore the variation between individuals is largely dictated by the latitude in which they primarily live. This will also limit the information that can be derived on variation of risk by age at exposure or time since exposure.

The present analysis uses total UVR as the exposure metric, which is the sum of UVA (320-400 nm) and UVB (280-320 nm) radiation, derived by scaling from total solar exposure (AVGLO) or UVB (NASA TOMS) [20]. UVA and UVB can all induce all major types of skin cancer, specifically melanoma, BCC and squamous cell carcinoma [44]. The mechanism for induction of BCC is likely cumulative mutational DNA damage to cells in the basal cell layer, specifically the interfollicular layer of epidermis [44–46]. There is no human exposure to ultraviolet C in sunlight, and UVA is markedly less efficient than UVB at causing DNA damage [44]. The ratio of solar UVA to UVB varies slightly with latitude [47], and there is more substantial variation over the course of a day, although not by much in the middle part of the day, when most exposure occurs [48]. As noted elsewhere, therefore cumulative UVR radiant exposure will be highly correlated with cumulative UVA or cumulative UVB, and so will be a natural measure to use in assessing the effects of UVR on skin cancer [20]. For these reasons we believe that cumulative UVB or cumulative UVR radiant exposure are the best currently available environmental exposures to use in assessing UVR-associated BCC risk.

We summarize the strengths and weaknesses of the two sets of UVR measurements in Table 5. As noted there, the main strengths of AVGLO is the fine geographical resolution, and that it is based on full-day recording of solar exposure on the ground, taking direct account of variations in cloud cover over the day. The NASA TOMS data, unlike AVGLO, is available worldwide, whereas AVGLO is only available for the continental US (Table 5). Both UVR measures rely on a simple scaling extrapolation, from total solar exposure (AVGLO) or from a single frequency in the UVB spectrum (TOMS). We found these two measures of UVR to be highly correlated [20] (see also Additional file 1: Figure S1), and associated risks remarkably similar, but as shown by the comparative AIC statistics, the linear-quadratic model of absolute risk fits better for AVGLO- than TOMS-derived UVR measures (Table 2).

**Table 5:** Strengths and weaknesses of AVGLO and NASA TOMS measures of ultraviolet radiation (UVR)

Measure	Strengths	Weaknesses
AVGLO	Fine geographical resolution (1 km^2^ grid).	Limited to continental USA
	Full day average of solar exposure.	Scaling required to get from total solar exposure to UVR.
	Ongoing data collection from 1960 to present.	
	Non-erythemally weighted UVR	
	Ground based (skyward facing)	
	Measured irradiances	
	Ground-based data collection, taking account of cloud cover throughout the day.	
NASA TOMS	Worldwide coverage.	Limited geographical resolution (1 degree longitude × 1 degree latitude).
	Space based (earth facing)	Measurements are based on inferred peak (midday) irradiance.
		Erythemally weighted UVR.
		Scaling required to get from UVB to UVR
		Modeled irradiances based on satellite O_3_ atmospheric absorption measures, taking account of elevation, solar zenith angle and time of sunrise/sunset
		Limited account taken of cloud cover (only as affects midday measurement).
		UVB measurements from 1978 to 1994 and 1996–2006. The TOMS Earth-Probe satellite failed in 2006, replaced by the Ozone Monitoring Instrument since then.

## Conclusions

To our knowledge this is the first analysis comparing risk of BCC in relation to the most recently used ground- and satellite-based metrics for ambient UVR. It is also the first analysis to report that BCC absolute risk is approximately proportional to cumulative radiant exposure, and that excess risks per unit of cumulative radiant exposure increase with increasing level of ambient UVR exposure, although the evidence is not completely clearcut on the second point because of the absence of curvature in the relative risk model. The considerations outlined in Table 5 suggest that the AVGLO measure is preferable, at least for analysis of the present US data. However, the fact that NASA TOMS UVR measures are available worldwide would add to its attractiveness were an international pooling study to be conducted. If confirmed in other datasets, our results suggests that interventions aimed at reducing risk of basal cell carcinoma should concentrate on those with the highest levels of ambient UVR exposure.

## Supplementary information

**Additional file 1 Table S1.** Categories for variables used to define person-year table. Supplementary statistical methods. **Table S2.** Curvature in exposure response of relative risk of basal cell carcinoma (BCC) with UVR cumulative radiant exposure, using AVGLO and NASA TOMS measures of UVR among 63,912 white technologists. **Table S3.** Modification by age of excess absolute risk and excess relative risk of basal cell carcinoma (BCC) in relation to ultraviolet radiation (UVR) cumulative radiant exposure. **Table S4.** Excess absolute risk (+ 95% CI) for AVGLO and NASA TOMS data. **Figure S1.** Cumulative potential UVR radiant exposure, derived from AVGLO and from NASA TOMS data, by age: (a) age 20 years, (b) age 40 years, (c) age 60 years, and (d) age 80 years. Reproduced from Little et al. [20].

**Additional file 2.** LQ1 questionnaire, LQ2 questionnaire, LQ3 questionnaire and supplementary skin cancer questionnaire used in USRT

## Data Availability

The datasets used and/or analyzed during the current study are available from the corresponding author (MPL) on reasonable request.

## Abbreviations

AIC: Akaike information criterion
ARRT: American Registry of Radiologic Technologists
AVGLO: Average daily total global
BCC: Basal cell carcinoma
CI: Confidence intervals
CIE: Commission Internationale de l’Eclairage
DOE: Department of Energy
GAM: Generalized additive model
NASA: National Aeronautics and Space Administration
NHS: Nurses’ Health Studies
NMSC: Non-melanoma skin cancers
NREL: National Renewable Energy Laboratory
NSRAD: National Solar Radiation Database
RB: Robertson-Berger
TOMS: Total Ozone Mapping Spectrometer
USRT: US radiologic technologists
UVA: Ultraviolet A
UVB: Ultraviolet B
UVR: Ultraviolet radiation
