# Supplementary material for: Cumulative solar ultraviolet radiation exposure and basal cell carcinoma of the skin in a nationwide US cohort using satellite and ground-based measures
Source: Environ Health. 2019 Dec 27;18:114. doi: 10.1186/s12940-019-0536-9 (PMC6935112; doi:10.1186/s12940-019-0536-9)

Table S1. Categories for variables used to define person-year table.

Description	Categories
Questionnaire at cohort first entry	Q1 vs Q2
Sex	Male vs female
Attained age, years	Categorised as 0 / 5/ 10 / … / 95 / 100 / 999999
Calendar year of follow-up	Categorised as <1980 / 1985 / 1990 / 1995 / 2000 / 2005 / 2009
Birth year	Categorised as <1900 / 1910 / 1920 / 1930 / 1940 / 1950 / 1960
Hormone replacement therapy (at Q1)	Yes vs no/unknown
Diuretic use (at Q2)	Yes vs no vs unknown
Strenuous exercise (at Q2)	Unknown vs <1 vs 1-3 vs 4+ hours per week
Baseline body mass index (BMI) in kg m^{-2}	Unknown vs < 18.5 vs 18.5-24.9 vs 25-29.9 vs 30.0+
Baseline cumulative head/neck dose (Gy)	0 / 0.001 / 0.002 / 0.005 / 0.010 / 0.020 / 0.050 / 0.100 / 0.200 / 0.500 / 1.000
Racial group (at baseline)	Categorised as white vs other
5-year lagged cumulative AVGLO UVR ambient radiant exposure (in kJ /cm^2)	Categorised as missing / 0 / 200 / 400 / 600 / 800 / 1000 / 1200 / 1400 / 1600 / 1800 / 2000 / 999999
5-year lagged cumulative TOMS UVR ambient radiant exposure (in kJ /cm^2)	Categorised as missing / 0 / 200 / 400 / 600 / 800 / 1000 / 1200 / 1400 / 1600 / 1800 / 2000 / 999999
Years from first exposure to point of follow-up	Categorised as -999999 [=NA] / -100 / 0 / 1 / 2 / 5 / 10 / 15 / 20 / 30 / 40 / 50 / 999999
Years from last exposure to point of follow-up	Categorised as -999999 [=NA] / -100 / 0 / 1 / 2 / 5 / 10 / 15 / 20 / 30 / 40 / 50 / 999999

Supplementary statistical methods.

In order to model the absolute risk associated with UVR we fitted a Poisson GAM [24], in which the expected number of cases in the stratum with person years PY , after cumulative UVR radiant exposure, $H(t)$ (in kJ cm^{-2}), at age t, and with various other explanatory covariates, $Z=(Z_i)$, was given by:

$$PY\left[\lambda\left(t,(Z_i)\,|\,(\phi_i),(\beta_i)\right)+\alpha H(t)\right] \qquad \text{(S1)}$$

The background model $\lambda\left(t,(Z_i)\,|\,(\phi_i),(\beta_i)\right)=\exp\left[\sum_{i=0}^{2}\phi_i\ln[t]^i+\sum_i\beta_i Z_i\right]$ for all BCCs was chosen by a forward-stepwise procedure [27]. It comprises loglinear terms in the baseline questionnaire, sex, ln[t], birth year, [birth year]^2, [birth year]^3, [birth year]^4, and [birth year]^5.

For most analyses we lag cumulative UVR radiant exposure by t_{lag} years, to reflect the likely induction period between cumulative UVR radiant exposure and induction of BCC, so that absolute risk is modelled by a linear-quadratic function of cumulative UVR radiant exposure:

$$PY\left[\lambda\left(t,(Z_i)\,|\,(\phi_i),(\beta_i)\right)+\alpha H(t-t_{lag})+\beta H(t-t_{lag})^2\right] \qquad \text{(S1')}$$

We also fitted linear-quadratic relative risk models, and exponential-linear-quadratic models, in which for example:

$$PY\lambda\left(t,(Z_i)\,|\,(\phi_i),(\beta_i)\right)\left[1+\alpha[H(t-t_{lag})-600]+\beta[H(t-t_{lag})-600]^2\right] \qquad \text{(S2)}$$

or:

$$PY\lambda\left(t,(Z_i)\,|\,(\phi_i),(\beta_i)\right)\exp\left[\alpha[H(t-t_{lag})-600]+\beta[H(t-t_{lag})-600]^2\right] \qquad \text{(S3)}$$

The approximate centering of the cumulative radiant exposure at 600 kJ cm^{-2} was done in order to facilitate model convergence; without this the relative risk models did not converge. However, analogous centering in the absolute risk model was not possible – when this was done the models generally failed to converge. A combined person year table was constructed for the AVGLO and TOMS data using Epicure [26]. Details of the stratification used to construct the person year table are given in Supplementary Table S1. For most analyses we assume $t_{lag}=5$

years. Model fitting was performed using R [25] and Epicure [26]. Confidence intervals (CI) were estimated from the profile likelihood [27], or if this did not converge using Wald-based CI.

We are also interested in exploring the effects of UVR accumulated in various intervals of exposure age, so that, for example, we fitted a relative risk model in which (ignoring the quadratic coefficients) risk associated with various levels of cumulative UVR radiant exposure before age 25, exposure at ages 25-34, 35-49, and 50+ was assumed to be given by:

$$PY\lambda\left(t,(Z_i)\,|\,(\phi_i),(\beta_i)\right)\begin{bmatrix}1+\alpha_1 H(\min[t-t_{lag},25])+\\ \alpha_2[H(\min[t-t_{lag},35])-H(\min[t-t_{lag},25])]+\\ \alpha_3[H(\min[t-t_{lag},50])-H(\min[t-t_{lag},35])]+\\ \alpha_4[H(t-t_{lag})-H(\min[t-t_{lag},50])]\end{bmatrix} \quad \text{(S4)}$$

and using also a similar model for absolute risk:

$$PY\begin{bmatrix}\lambda\left(t,(Z_i)\,|\,(\phi_i),(\beta_i)\right)+\alpha_1 H(\min[t-t_{lag},25])+\\ \alpha_2[H(\min[t-t_{lag},35])-H(\min[t-t_{lag},25])]+\\ \alpha_3[H(\min[t-t_{lag},50])-H(\min[t-t_{lag},35])]+\\ \alpha_4[H(t-t_{lag})-H(\min[t-t_{lag},50])]\end{bmatrix} \quad \text{(S5)}$$

Here $H(\min[t-t_{lag},25])$ measures the cumulative UVR radiant exposure before age 25, $[H(\min[t-t_{lag},35])-H(\min[t-t_{lag},25])]$ measures cumulative UVR radiant exposure between the ages of 25 and 35, $[H(\min[t-t_{lag},50])-H(\min[t-t_{lag},35])]$ measures cumulative UVR radiant exposure between the ages of 35 and 50, and $[H(t-t_{lag})-H(\min[t-t_{lag},50])]$ measures cumulative UVR radiant exposure above the age of 50. The cutpoints of 25, 35, and 50 years of age were chosen somewhat arbitrarily, reflecting possible effects in early, middle and older ages.

Likewise, to explore the effects of cumulative UVR radiant exposure in various intervals before time at risk, we assumed, for example, that risk for cumulative UVR radiant exposure in the intervals 5-9, 10-14 and 15 years or more before the time at risk is given by:

$$PY\lambda\left(t,(Z_i)\,|\,(\phi_i),(\beta_i)\right)\begin{bmatrix}1+\alpha_1[H(t-5)-H(t-10)]+\\ \alpha_2[H(t-10)-H(t-15)]+\\ \alpha_3 H(t-15)\end{bmatrix} \tag{S6}$$

and using also a similar model for absolute risk:

$$PY\begin{bmatrix}\lambda\left(t,(Z_i)\,|\,(\phi_i),(\beta_i)\right)+\alpha_1[H(t-5)-H(t-10)]+\\ \alpha_2[H(t-10)-H(t-15)]+\\ \alpha_3 H(t-15)\end{bmatrix} \tag{S7}$$

Here $[H(t-5)-H(t-10)]$ measures the cumulative UVR radiant exposure between 5 and 10 years before the time at risk, $[H(t-10)-H(t-15)]$ measures cumulative UVR radiant exposure between 10 and 15 years before the time at risk, and $H(t-15)$ measures cumulative UVR radiant exposure 15 or more years before the time at risk. The cutpoints of 5, 10 and 15 years were chosen somewhat arbitrarily, in the light of knowledge of the period of follow-up.

Table S2. Curvature in exposure response of relative risk of basal cell carcinoma (BCC) with UVR cumulative radiant exposure, using AVGLO and NASA TOMS measures of UVR among 63,912 white technologists. [a]

Model	Linear excess relative risk per MJ/cm^2 [centered at 600 kJ/ cm^2] (+95% CI)	Quadratic excess relative risk per [MJ/cm^2]^2 [centered at 600/ kJ cm^2] (+95% CI)	*p*-value	AIC
		AVGLO		
Linear	3.84 (2.13, 5.77[b])		<0.001[c]	11,903.6
Linear-quadratic	3.85 (2.01, 5.85[b])	-0.04 (-1.82[b], 2.30)	0.964[d]	11,905.6
		NASA TOMS		
Linear	1.60 (0.92, 2.29[b])		<0.001[c]	11,913.6
Linear-quadratic	1.98[e] (1.19, 2.85[b])	-0.68[e] (-1.61[b], 0.13)	0.089[d e]	11,912.7[e]
**Model**	**Log-linear excess relative risk per MJ/cm^2 [centered at 600 kJ/ cm^2] (+95% CI)**		***p*-value**	**AIC**
		AVGLO		
Log-linear	1.16 (0.75, 1.57)		<0.001[f]	11,915.7
		NASA TOMS		
Log-linear	0.92 (0.62, 1.23)		<0.001[f]	11,920.3

[a]All analysis used linear-quadratic model (S1') with adjustment to the baseline BCC rate for baseline questionnaire, ln[age], birth year, [birth year]^2, [birth year]^3, [birth year]^4, [birth year]^5.
[b]Wald-based CI.
[c]test for departure of UVR linear exposure-response from null.
[d]test for departure of UVR linear-quadratic exposure-response from linearity.
[e]indications of non-convergence.
[f]test for departure of UVR exponential exposure-response from null.

Table S3. Modification by age of excess absolute risk and excess relative risk of basal cell carcinoma (BCC) in relation to AVGLO-derived ultraviolet radiation (UVR) cumulative radiant exposure.[a]

Absolute risk models		
Excess absolute risk per cumulative UVR radiant exposure (MJ cm^{-2}) per 10^4 person year, adjusted for age, centered at 46.77 y	Excess absolute risk change per 5 years of age	*p*-value
11.4 (8.08, 13.9)	1.06 (0.96, 1.14)	0.185
Relative risk models		
Excess relative risk per cumulative UVR radiant exposure (MJ cm^{-2}), adjusted for age, centered at 46.77 y	Excess relative risk change per 5 years of age	*p*-value
2.16[b] (-2.90[c], 7.22[c])	0.78[b] (0.39[c], 1.55[c])	0.089[b]

[a]All analysis used a model with adjustment to the baseline BCC rate for baseline questionnaire, ln[age], $ln[age]^2$, birth year, $[birth year]^2$, $[birth year]^3$, $[birth year]^4$, $[birth year]^5$.
[b]indications of lack of convergence.
[c]Wald-based confidence interval.

Table S4. Excess absolute risk (+95% CI) for AVGLO and NASA TOMS data

Cumulative UVR radiant exposure range (kJ cm^{-2})	AVGLO			NASA TOMS		
	Mean cumulative UVR radiant exposure (kJ cm^{-2})	Person years	EAR (/10^4 PY) (+95% CI)	Mean cumulative UVR radiant exposure (kJ cm^{-2})	Person years	EAR (/10^4 PY) (+95% CI)
0-599	539.52	80,259	0.00	513.50	241,103	0.00
600-799	711.18	293,956	0.36 (0.12, 0.71)	699.18	403,617	0.16 (0.02, 0.32)
800-999	896.05	361,480	0.66 (0.29, 1.24)	888.79	268,795	0.30 (0.09, 0.57)
1000-1199	1087.59	218,161	1.28 (0.66, 2.26)	1083.42	117,671	0.85 (0.48, 1.30)
1200-1399	1282.94	90,284	2.24 (1.29, 3.74)	1279.61	37,841	1.01 (0.43, 1.71)
1400-1599	1479.79	28,746	2.22 (0.98, 4.04)	1478.85	10,728	1.45 (0.44, 2.71)
1600+	1724.59	9867	2.90 (1.01, 5.55)	1702.13	2997	1.95 (0.07, 4.50)

Figure S1. Cumulative potential UVR radiant exposure, derived from AVGLO and from NASA TOMS data, by age: (a) age 20 years, (b) age 40 years, (c) age 60 years, and (d) age 80 years. Reproduced from Little *et al.* [20]

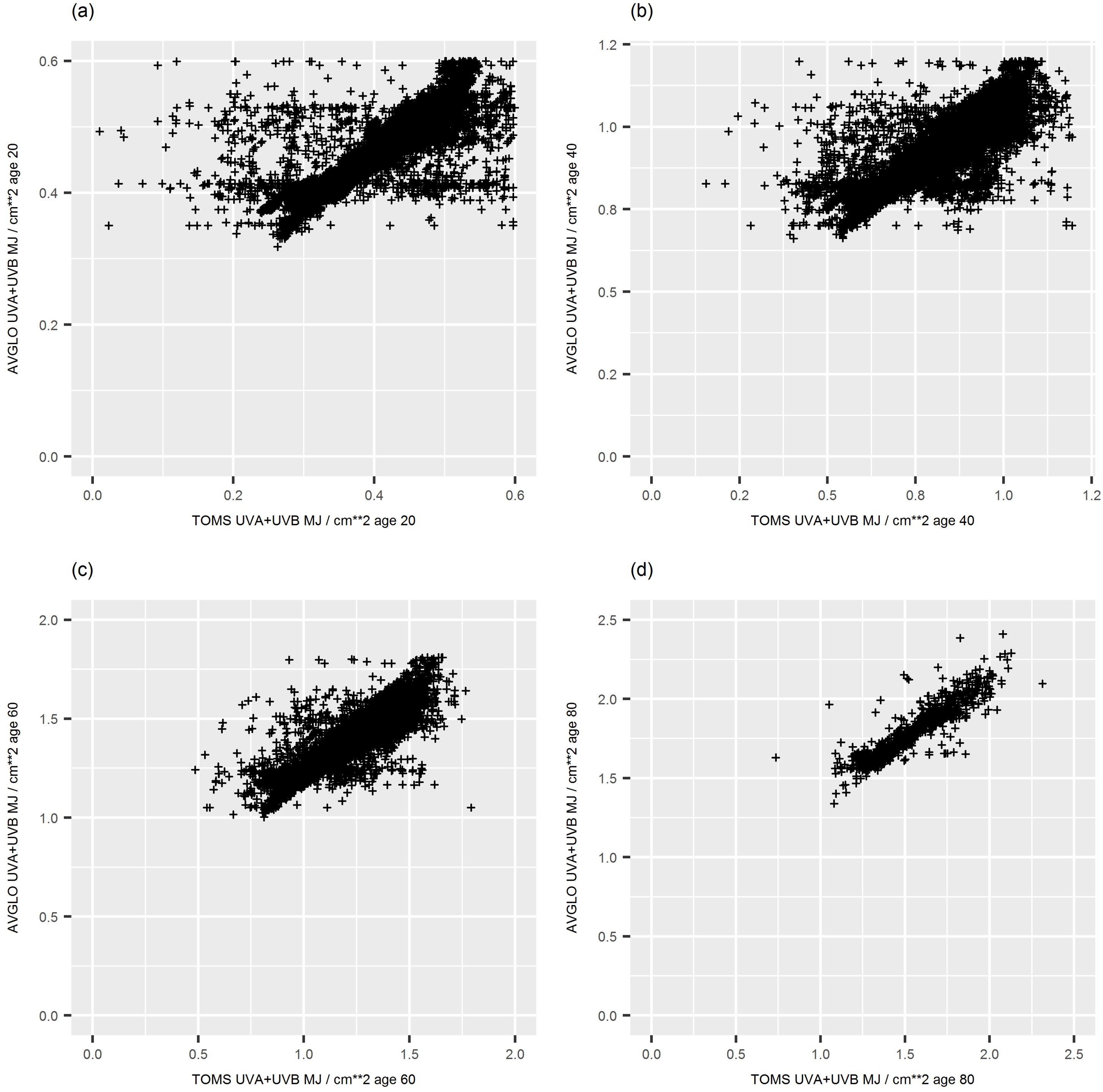

Supplement: Supplementary file 1 — Additional file 1 Table S1. Categories for variables used to define person-year table. Supplementary statistical methods. Table S2. Curvature in exposure response of relative risk of basal cell carcinoma (BCC) with UVR cumulative radiant exposure, using AVGLO and NASA TOMS measures of UVR among 63,912 white technologists. Table S3. Modification by age of excess absolute risk and excess relative risk of basal cell carcinoma (BCC) in relation to ultraviolet radiation (UVR) cumulative radiant exposure. Table S4. Excess absolute risk (+ 95% CI) for AVGLO and NASA TOMS data. Figure S1. Cumulative potential UVR radiant exposure, derived from AVGLO and from NASA TOMS data, by age: (a) age 20 years, (b) age 40 years, (c) age 60 years, and (d) age 80 years. Reproduced from Little et al. [20]. [file 12940_2019_536_MOESM1_ESM.pdf]
