# Supplementary material for: Cumulative solar ultraviolet radiation exposure and basal cell carcinoma of the skin in a nationwide US cohort using satellite and ground-based measures
Source: Environ Health. 2019 Dec 27;18:114. doi: 10.1186/s12940-019-0536-9 (PMC6935112; doi:10.1186/s12940-019-0536-9)
Supplement: Supplementary file 2 — Additional file 2. LQ1 questionnaire, LQ2 questionnaire, LQ3 questionnaire and supplementary skin cancer questionnaire used in USRT [file 12940_2019_536_MOESM2_ESM.pdf]

OMB NO. 0925-0164 1.31.86

University of Minnesota
American Registry of Radiologic Technologists
National Institutes of Health Collaborative Health Study

Division of Epidemiology
University of Minnesota
Minneapolis, Minnesota 55455

8 4 2 1

8 4 2 1

8 4 2 1

8 4 2 1

8 4 2 1

8 4 2 1

8 4 2 1

Directions: Your responses will be read by an optical reader. By carefully observing the few simple rules below, the accurate recording of your responses will be ensured.

- Use black lead pencil only (No. 2½ or softer).
- Do NOT use ink or ballpoint pens.
- Make heavy black marks that fill the circle completely.
- Erase cleanly any answer you wish to change.
- Make no stray marks on the answer sheet.

EXAMPLES

Proper Mark ◯⬤◯◯ Improper Marks ✓ ✗ ⊙ ○

This survey contains several questions which ask you to write a response. Please PRINT your response and confine it to the box provided. Other questions ask you to record number answers on a two digit grid. First write the number in the space provided, then mark the circles of the number in the grid below. For example:

A. To record a year (Example: 1916) — 1916

B. To record a two digit number (Example: 27) — 27

C. To record a number less than 10: fill in "0", then the number (Example: 3) — 03

It is important not to write in the booklet except where indicated. Space has been provided on page 15 for any additional information or comments you may have.

I. GENERAL INFORMATION

1. What is your birth date?

MONTH: ◯ Jan ◯ Feb ◯ Mar ◯ Apr ◯ May ◯ Jun ◯ Jul ◯ Aug ◯ Sep ◯ Oct ◯ Nov ◯ Dec

DAY: [ ][ ]

YEAR: 1[ ][ ][ ]

2. What is your sex?
   - ◯ Male
   - ◯ Female

3. Which of the following groups best describes your racial background?
   - ◯ White, not of Hispanic origin
   - ◯ Black, not of Hispanic origin
   - ◯ Hispanic
   - ◯ American Indian or Alaskan Native
   - ◯ Asian or Pacific Islander
   - ◯ Other (describe below)

PLEASE PRINT — STAY WITHIN BOX

4. About how tall are you without shoes?

FEET [ ] INCHES [ ][ ]

5. About how much do you weigh without clothes or shoes?

POUNDS [ ][ ][ ]

6. How many years of schooling have you completed?
   - ◯ 1–8 years (grade school)
   - ◯ 9–12 years (high school)
   - ◯ 2 years radiologic technology program (hospital)
   - ◯ 1–4 years college
   - ◯ Graduate school
   - ◯ 1–3 years vocational education beyond high school
   - ◯ Something else

PLEASE PRINT — STAY WITHIN BOX

7. Are you currently married, widowed, divorced or separated, or have you never been married?
   - ◯ Currently married
   - ◯ Widowed
   - ◯ Divorced or separated
   - ◯ Never married

8. Have you smoked at least 100 cigarettes during your entire life?

◯ Yes ◯ No

IF YOU MARKED NO, SKIP TO QUESTION 13.

9. How old were you when you started smoking?

YEARS OLD

⓪	⓪
①	①
②	②
③	③
④	④
⑤	⑤
⑥	⑥
⑦	⑦
⑧	⑧
⑨	⑨

10. Do you smoke cigarettes now?

◯ Yes ◯ No

IF YOU MARKED YES, SKIP TO QUESTION 12.

11. How old were you when you stopped smoking?

YEARS OLD

⓪	⓪
①	①
②	②
③	③
④	④
⑤	⑤
⑥	⑥
⑦	⑦
⑧	⑧
⑨	⑨

12. On the average, how much do you or did you smoke each day?

◯ Less than ½ pack a day
◯ ½ to 1 pack a day
◯ Between 1 and 2 packs a day
◯ More than 2 packs a day

13. How many drinks of alcoholic beverage (beer, wine, or liquor) do you usually have in a typical week?

◯ Never drink
◯ Less than once a week
◯ 1–2 drinks a week
◯ 3–6 drinks a week
◯ 7–10 drinks a week
◯ 11–12 drinks a week
◯ 13–14 drinks a week
◯ More than 14 drinks a week

14. Have you ever used permanent hair dye regularly in your hair? By regularly we mean at least twice a year for 2 consecutive years. (Please do not include temporary rinses.)

◯ Yes ◯ No

IF YOU MARKED NO, SKIP TO QUESTION 17.

15. About how often did you or do you use permanent hair dyes?

About every [ ] [ ] week(s)

⓪	⓪
①	①
②	②
③	③
④	④
⑤	⑤
⑥	⑥
⑦	⑦
⑧	⑧
⑨	⑨

16. For about how many years have you used permanent hair dye regularly?

About [ ] [ ] year(s)

⓪	⓪
①	①
②	②
③	③
④	④
⑤	⑤
⑥	⑥
⑦	⑦
⑧	⑧
⑨	⑨

17. Are you currently working as a radiologic technologist or radiologic technician?

◯ Yes
◯ No

IF YOU MARKED YES, SKIP TO QUESTION 19.

18. Have you ever worked as a radiologic technologist or radiologic technician?

◯ Yes
◯ No

IF YOU MARKED NO, SKIP TO QUESTION 25.

19. Please complete the following for all the jobs where you were employed as a radiologic technologist or radiologic technician. Start with your most recent job and end with your first job. Fill in (a) place of employment, (b) name and address of employment, (c) whether or not you wore a dosimeter most of the time, (d) year employment began and the length of employment in years and months. Please include your hospital training program.

1a. Place of employment
- ◯ Hospital
- ◯ Physician's office
- ◯ Other (please specify)

c. DOSIMETER ◯ Yes ◯ No

d. Year began 19 ☐☐ (0 0 – 9 9)

Length of Employment: Yrs. ☐☐ (0 0 – 9 9) Mos. (1 – 11)

b. Name of place of employment ____ Address ____ City ____ State ____ ZIP ____

2a. Place of employment
- ◯ Hospital
- ◯ Physician's office
- ◯ Other (please specify)

c. DOSIMETER ◯ Yes ◯ No

d. Year began 19 ☐☐ (0 0 – 9 9)

Length of Employment: Yrs. ☐☐ (0 0 – 9 9) Mos. (1 – 11)

b. Name of place of employment ____ Address ____ City ____ State ____ ZIP ____

3a. Place of employment
- ◯ Hospital
- ◯ Physician's office
- ◯ Other (please specify)

c. DOSIMETER ◯ Yes ◯ No

d. Year began 19 ☐☐ (0 0 – 9 9)

Length of Employment: Yrs. ☐☐ (0 0 – 9 9) Mos. (1 – 11)

b. Name of place of employment ____ Address ____ City ____ State ____ ZIP ____

4a. Place of employment
- ◯ Hospital
- ◯ Physician's office
- ◯ Other (please specify)

c. DOSIMETER ◯ Yes ◯ No

d. Year began 19 ☐☐ (0 0 – 9 9)

Length of Employment: Yrs. ☐☐ (0 0 – 9 9) Mos. (1 – 11)

b. Name of place of employment ____ Address ____ City ____ State ____ ZIP ____

5a. Place of employment
- ◯ Hospital
- ◯ Physician's office
- ◯ Other (please specify)

c. DOSIMETER ◯ Yes ◯ No

d. Year began 19 ☐☐ (0 0 – 9 9)

Length of Employment: Yrs. ☐☐ (0 0 – 9 9) Mos. (1 – 11)

b. Name of place of employment ____ Address ____ City ____ State ____ ZIP ____

6a. Place of employment
- ◯ Hospital
- ◯ Physician's office
- ◯ Other (please specify)

c. DOSIMETER ◯ Yes ◯ No

d. Year began 19 ☐☐ (0 0 – 9 9)

Length of Employment: Yrs. ☐☐ (0 0 – 9 9) Mos. (1 – 11)

b. Name of place of employment ____ Address ____ City ____ State ____ ZIP ____

7a. Place of employment
- ◯ Hospital
- ◯ Physician's office
- ◯ Other (please specify)

c. DOSIMETER ◯ Yes ◯ No

d. Year began 19 ☐☐ (0 0 – 9 9)

Length of Employment: Yrs. ☐☐ (0 0 – 9 9) Mos. (1 – 11)

b. Name of place of employment ____ Address ____ City ____ State ____ ZIP ____

8a. Place of employment
- ◯ Hospital
- ◯ Physician's office
- ◯ Other (please specify)

c. DOSIMETER ◯ Yes ◯ No

d. Year began 19 ☐☐ (0 0 – 9 9)

Length of Employment: Yrs. ☐☐ (0 0 – 9 9) Mos. (1 – 11)

b. Name of place of employment ____ Address ____ City ____ State ____ ZIP ____

20. While employed as a radiologic technologist or radiologic technician have you ever worked or assisted with any of the following procedures? Please mark "yes" or "no" for each procedure. For each "yes" fill in the year you began work with that procedure and the length of time in years and months you worked with the procedure. Please include your hospital training program.

IF YOU ARE UNABLE TO PROVIDE THE EXACT YEAR YOU BEGAN WORK WITH THAT PROCEDURE, OR THE LENGTH OF TIME, PLEASE PROVIDE YOUR BEST ESTIMATE.

1–8 Diagnostic

1. Fluoroscopy

Ever used? ◯ Yes ◯ No ◯ Don't know

Year began: 19 ☐☐ (0–9) (0–9)
Total Years ☐☐ (0–9) (0–9) Mos. (1–11)

2. Dental X-ray

Ever used? ◯ Yes ◯ No ◯ Don't know

Year began: 19 ☐☐ (0–9) (0–9)
Total Years ☐☐ (0–9) (0–9) Mos. (1–11)

3. Routine X-ray other than fluoroscopic film & dental

Ever used? ◯ Yes ◯ No ◯ Don't know

Year began: 19 ☐☐ (0–9) (0–9)
Total Years ☐☐ (0–9) (0–9) Mos. (1–11)

4. Multi-film procedures (e.g. IVP)

Ever used? ◯ Yes ◯ No ◯ Don't know

Year began: 19 ☐☐ (0–9) (0–9)
Total Years ☐☐ (0–9) (0–9) Mos. (1–11)

5. CAT Scan

Ever used? ◯ Yes ◯ No ◯ Don't know

Year began: 19 ☐☐ (0–9) (0–9)
Total Years ☐☐ (0–9) (0–9) Mos. (1–11)

6. Portable X-ray

Number of times
◯ Never
◯ 1–9
◯ 10–24
◯ 25–49
◯ 50+

7. Diagnostic radioisotopes (e.g. I-131 uptakes)

Ever used? ◯ Yes ◯ No ◯ Don't know

Year began: 19 ☐☐ (0–9) (0–9)
Total Years ☐☐ (0–9) (0–9) Mos. (1–11)

8. Diagnostic ultrasound

Ever used? ◯ Yes ◯ No ◯ Don't know

Year began: 19 ☐☐ (0–9) (0–9)
Total Years ☐☐ (0–9) (0–9) Mos. (1–11)

9–15 Therapy

9. Orthovoltage (200-400 kVp)

Ever used? ◯ Yes ◯ No ◯ Don't know

Year began: 19 ☐☐ (0–9) (0–9)
Total Years ☐☐ (0–9) (0–9) Mos. (1–11)

10. Cobalt 60

Ever used? ◯ Yes ◯ No ◯ Don't know

Year began: 19 ☐☐ (0–9) (0–9)
Total Years ☐☐ (0–9) (0–9) Mos. (1–11)

11. Betatron

Ever used? ◯ Yes ◯ No ◯ Don't know

Year began: 19 ☐☐ (0–9) (0–9)
Total Years ☐☐ (0–9) (0–9) Mos. (1–11)

12. Other X-ray teletherapy (LINAC)

Ever used? ◯ Yes ◯ No ◯ Don't know

Year began: 19 ☐☐ (0–9) (0–9)
Total Years ☐☐ (0–9) (0–9) Mos. (1–11)

13. Radium therapy

Ever used? ◯ Yes ◯ No ◯ Don't know

Year began: 19 ☐☐ (0–9) (0–9)
Total Years ☐☐ (0–9) (0–9) Mos. (1–11)

14. Other radio-isotope therapy

Ever used? ◯ Yes ◯ No ◯ Don't know

Year began: 19 ☐☐ (0–9) (0–9)
Total Years ☐☐ (0–9) (0–9) Mos. (1–11)

15. Microwave or Ultrasound diathermy

Ever used? ◯ Yes ◯ No ◯ Don't know

Year began: 19 ☐☐ (0–9) (0–9)
Total Years ☐☐ (0–9) (0–9) Mos. (1–11)

21a. If you wore a dosimetry badge (film badge, TLD, pocket chamber, etc.) where did you usually wear it? (Mark only the one that is used most often.)

◯ Never wore a dosimeter

◯ Belt loop, waist or side pocket

◯ Breast pocket

◯ Lapel

◯ Other (describe below)

PLEASE PRINT —
STAY WITHIN BOX

[ ]

21b. Did you also usually wear a hand or wrist dosimeter?

◯ Yes ◯ No

22. When you were first working as a radiologic technologist or radiologic technician, did you usually wear a lead apron or stand behind a protective shield?

◯ Yes ◯ No

IF YOU ARE NOT CURRENTLY WORKING AS A RADIOLOGIC TECHNOLOGIST OR RADIOLOGIC TECHNICIAN, SKIP TO QUESTION 25.

23. Do you usually wear a lead apron or stand behind a shield now?

◯ Yes ◯ No

IF YOU MARKED NO, SKIP TO QUESTION 25.

24. When you wear an apron, where do you usually wear your dosimetry badge?

◯ Don't usually wear a badge.
◯ Under the apron
◯ Outside the apron
◯ Varies, sometimes under sometimes outside
◯ Badge is not worn but is located in X-ray room
(Describe below)

[ ]

25. Were you ever employed in a position other than as a radiologic technologist or radiologic technician for more than one year?

◯ Yes
◯ No

IF YOU MARKED NO, SKIP TO QUESTION 28.

26. Other than as a radiologic technologist or radiologic technician, what was the occupation or job in which you were employed for the longest time? (please specify)

[ ]

NAME OF OCCUPATION

27. In what year did you first work in a position other than as a radiologic technologist or radiologic technician?

19 [ ][ ]

0	0
1	1
2	2
3	3
4	4
5	5
6	6
7	7
8	8
9	9

27a. Altogether, approximately how many years did you work in a position or positions other than as a radiologic technologist or radiologic technician?

[ ][ ] YEARS

0	0
1	1
2	2
3	3
4	4
5	5
6	6
7	7
8	8
9	9

28. Approximately how many times have you held a person for an X-ray?

◯ Never
◯ 1–9 times
◯ 10–24 times
◯ 25–49 times
◯ 50+ times

II. MEDICAL HISTORY

29. Have you ever been told by a doctor that you had a thyroid condition, for example, thyroid nodules, thyroid cancer, hyperthyroidism, hypothyroidism, goiter, etc.?

◯ Yes ◯ No ◯ Don't know

IF YOU MARKED NO OR DON'T KNOW, SKIP TO QUESTION 33.

30. **What was the specific medical name for the thyroid condition(s)?** (Mark all that apply)

◯ Hyperthyroidism ◯ Thyroid cancer
◯ Hypothyroidism ◯ Goiter
◯ Thyroiditis ◯ Other (please specify →)

PLEASE PRINT — STAY WITHIN BOX

◯ Don't know exact medical name

31. **When was the thyroid condition first diagnosed?**

MONTH

◯ Jan ◯ May ◯ Sep
◯ Feb ◯ Jun ◯ Oct
◯ Mar ◯ Jul ◯ Nov
◯ Apr ◯ Aug ◯ Dec

YEAR

19 __ __

0 0
1 1
2 2
3 3
4 4
5 5
6 6
7 7
8 8
9 9

32. **What was the name and address of the doctor or hospital that diagnosed the thyroid condition?**

Name: ____________________

Address: ____________________
Street

City State ZIP

33. **Have you ever been told by a doctor that you had leukemia, Hodgkin's disease, multiple myeloma, or any other type of cancer?**

◯ Yes ◯ No ◯ Don't know

IF YOU MARKED NO, SKIP TO QUESTION 35.

34. Please fill in the information asked below for each type of cancer you had.
(a) What was the type of cancer?
(b) In what month and year did the doctor tell you about the cancer?
(c) What was the name and address of the doctor or hospital that first treated you for this cancer?
(Space is provided for up to 3 cancer types.)

a. Type of cancer (Mark only one)

◯ Lung ◯ Multiple Myeloma ◯ Cervix
◯ Stomach ◯ Uterus
◯ Colon ◯ Skin ◯ Ovary
◯ Rectum ◯ Prostate ◯ Other (please specify the site of the cancer)
◯ Leukemia ◯ Breast
◯ Hodgkin's disease

c. ____________________
Name of doctor or hospital

d. ____________________
Street

City State ZIP

b. Date when diagnosed

Mo. Year

◯ Jan
◯ Feb 0 0
◯ Mar 1 1
◯ Apr 2 2
◯ May 3 3
◯ Jun 4 4
◯ Jul 5 5
◯ Aug 6 6
◯ Sep 7 7
◯ Oct 8 8
◯ Nov 9 9
◯ Dec

a. Type of cancer (Mark only one)

◯ Lung ◯ Multiple Myeloma ◯ Cervix
◯ Stomach ◯ Uterus
◯ Colon ◯ Skin ◯ Ovary
◯ Rectum ◯ Prostate ◯ Other (please specify the site of the cancer)
◯ Leukemia ◯ Breast
◯ Hodgkin's disease

c. ____________________
Name of doctor or hospital

d. ____________________
Street

City State ZIP

b. Date when diagnosed

Mo. Year

◯ Jan
◯ Feb 0 0
◯ Mar 1 1
◯ Apr 2 2
◯ May 3 3
◯ Jun 4 4
◯ Jul 5 5
◯ Aug 6 6
◯ Sep 7 7
◯ Oct 8 8
◯ Nov 9 9
◯ Dec

a. Type of cancer (Mark only one)

◯ Lung ◯ Multiple Myeloma ◯ Cervix
◯ Stomach ◯ Uterus
◯ Colon ◯ Skin ◯ Ovary
◯ Rectum ◯ Prostate ◯ Other (please specify the site of the cancer)
◯ Leukemia ◯ Breast
◯ Hodgkin's disease

c. ____________________
Name of doctor or hospital

d. ____________________
Street

City State ZIP

b. Date when diagnosed

Mo. Year

◯ Jan
◯ Feb 0 0
◯ Mar 1 1
◯ Apr 2 2
◯ May 3 3
◯ Jun 4 4
◯ Jul 5 5
◯ Aug 6 6
◯ Sep 7 7
◯ Oct 8 8
◯ Nov 9 9
◯ Dec

35. Have you ever been told by a doctor that you have had a myocardial infarction (heart attack)?

- ◯ Yes
- ◯ No
- ◯ Don't know

IF YOU MARKED NO, SKIP TO QUESTION 37.

36. How old were you the first time you had a myocardial infarction?

AGE
0	0
1	1
2	2
3	3
4	4
5	5
6	6
7	7
8	8
9	9

37. Have you ever had any children? (Please only include children born live. Don't include adopted or foster children.)

- ◯ Yes
- ◯ No

IF YOU MARKED NO, SKIP TO QUESTION 43 NEXT.

38. What is the date of birth of your first child?

MONTH	DAY		YEAR	
			19	
JAN ◯	0	0	0	0
FEB ◯	1	1	1	1
MAR ◯	2	2	2	2
APR ◯	3	3	3	3
MAY ◯	4	4	4	4
JUN ◯	5	5	5	5
JUL ◯	6	6	6	6
AUG ◯	7	7	7	7
SEP ◯	8	8	8	8
OCT ◯	9	9	9	9
NOV ◯				
DEC ◯				

39. Were any of your children born with a birth defect?

- ◯ Yes
- ◯ No

IF YOU MARKED NO, SKIP TO QUESTION 41

40. Please complete the following information about each child born to you with a birth defect. (a) Date of birth (b) Sex (c) Nature of birth defect(s) (d) Hospital where diagnosed

Child 1

a. Date of birth

Month	Year 19	
JAN ◯	0	0
FEB ◯	1	1
MAR ◯	2	2
APR ◯	3	3
MAY ◯	4	4
JUN ◯	5	5
JUL ◯	6	6
AUG ◯	7	7
SEP ◯	8	8
OCT ◯	9	9
NOV ◯		
DEC ◯		

b. Sex ◯ Male ◯ Female

c. Nature of defect(s)

d. Hospital where diagnosed

Name of hospital

Street

City State ZIP

Child 2

a. Date of birth

Month	Year 19	
JAN ◯	0	0
FEB ◯	1	1
MAR ◯	2	2
APR ◯	3	3
MAY ◯	4	4
JUN ◯	5	5
JUL ◯	6	6
AUG ◯	7	7
SEP ◯	8	8
OCT ◯	9	9
NOV ◯		
DEC ◯		

b. Sex ◯ Male ◯ Female

c. Nature of defect(s)

d. Hospital where diagnosed

Name of hospital

Street

City State ZIP

Child 3

a. Date of birth

Month	Year 19	
JAN ◯	0	0
FEB ◯	1	1
MAR ◯	2	2
APR ◯	3	3
MAY ◯	4	4
JUN ◯	5	5
JUL ◯	6	6
AUG ◯	7	7
SEP ◯	8	8
OCT ◯	9	9
NOV ◯		
DEC ◯		

b. Sex ◯ Male ◯ Female

c. Nature of defect(s)

d. Hospital where diagnosed

Name of hospital

Street

City State ZIP

41. Have any of your children died?

◯ Yes ◯ No

IF YOU MARKED NO, SKIP TO QUESTION 43.

42. Complete the following information about each child that died. (a) Sex (b) Date of birth (c) Date of death and (d) Cause of death.

Child 1 a. Sex: ◯ Male ◯ Female

b. Date of birth MONTH	YEAR	c. Date of death MONTH	YEAR	d. Cause of death (please specify)
◯ Jan	19 [ ][ ]	◯ Jan	19 [ ][ ]	
◯ Feb		◯ Feb		
◯ Mar	⓪⓪	◯ Mar	⓪⓪	
◯ Apr	①①	◯ Apr	①①	
◯ May	②②	◯ May	②②	PLEASE WRITE IN BOX ONLY
◯ Jun	③③	◯ Jun	③③	
◯ Jul	④④	◯ Jul	④④	
◯ Aug	⑤⑤	◯ Aug	⑤⑤	
◯ Sep	⑥⑥	◯ Sep	⑥⑥	
◯ Oct	⑦⑦	◯ Oct	⑦⑦	
◯ Nov	⑧⑧	◯ Nov	⑧⑧	
◯ Dec	⑨⑨	◯ Dec	⑨⑨	

Child 2 a. Sex ◯ Male ◯ Female

b. Date of birth MONTH	YEAR	c. Date of death MONTH	YEAR	d. Cause of death (please specify)
◯ Jan	19 [ ][ ]	◯ Jan	19 [ ][ ]	
◯ Feb		◯ Feb		
◯ Mar	⓪⓪	◯ Mar	⓪⓪	
◯ Apr	①①	◯ Apr	①①	
◯ May	②②	◯ May	②②	PLEASE WRITE IN BOX ONLY
◯ Jun	③③	◯ Jun	③③	
◯ Jul	④④	◯ Jul	④④	
◯ Aug	⑤⑤	◯ Aug	⑤⑤	
◯ Sep	⑥⑥	◯ Sep	⑥⑥	
◯ Oct	⑦⑦	◯ Oct	⑦⑦	
◯ Nov	⑧⑧	◯ Nov	⑧⑧	
◯ Dec	⑨⑨	◯ Dec	⑨⑨	

Child 3 a. Sex ◯ Male ◯ Female

b. Date of birth MONTH	YEAR	c. Date of death MONTH	YEAR	d. Cause of death (please specify)
◯ Jan	19 [ ][ ]	◯ Jan	19 [ ][ ]	
◯ Feb		◯ Feb		
◯ Mar	⓪⓪	◯ Mar	⓪⓪	
◯ Apr	①①	◯ Apr	①①	
◯ May	②②	◯ May	②②	PLEASE WRITE IN BOX ONLY
◯ Jun	③③	◯ Jun	③③	
◯ Jul	④④	◯ Jul	④④	
◯ Aug	⑤⑤	◯ Aug	⑤⑤	
◯ Sep	⑥⑥	◯ Sep	⑥⑥	
◯ Oct	⑦⑦	◯ Oct	⑦⑦	
◯ Nov	⑧⑧	◯ Nov	⑧⑧	
◯ Dec	⑨⑨	◯ Dec	⑨⑨	

43. For each of the following SPECIAL X-RAY PROCEDURES, APPROXIMATE (a) whether you ever had the procedure. For each procedure you had, APPROXIMATE (b) the number of times you had the procedure, and (c) the year you had the procedure for the first time.

1. Have you ever had a Barium Enema?

◯ Yes ◯ No ◯ Don't Know

Approximate No. of times	Approximate Yr. first done
[ ][ ]	19 [ ][ ]
⓪⓪ ①① ②② ③③ ④④ ⑤⑤ ⑥⑥ ⑦⑦ ⑧⑧ ⑨⑨	⓪⓪ ①① ②② ③③ ④④ ⑤⑤ ⑥⑥ ⑦⑦ ⑧⑧ ⑨⑨

2. Have you ever had a Cholecystogram or a Cholangiogram?

◯ Yes ◯ No ◯ Don't Know

Approximate No. of times	Approximate Yr. first done
[ ][ ]	19 [ ][ ]
⓪⓪ ①① ②② ③③ ④④ ⑤⑤ ⑥⑥ ⑦⑦ ⑧⑧ ⑨⑨	⓪⓪ ①① ②② ③③ ④④ ⑤⑤ ⑥⑥ ⑦⑦ ⑧⑧ ⑨⑨

3. Have you ever had an Intravenous or Retrograde Pyelogram?

◯ Yes ◯ No ◯ Don't Know

Approximate No. of times	Approximate Yr. first done
[ ][ ]	19 [ ][ ]
⓪⓪ ①① ②② ③③ ④④ ⑤⑤ ⑥⑥ ⑦⑦ ⑧⑧ ⑨⑨	⓪⓪ ①① ②② ③③ ④④ ⑤⑤ ⑥⑥ ⑦⑦ ⑧⑧ ⑨⑨

4. Have you ever had a Renal Arteriogram?

◯ Yes ◯ No ◯ Don't Know

Approximate No. of times	Approximate Yr. first done
[ ][ ]	19 [ ][ ]
⓪⓪ ①① ②② ③③ ④④ ⑤⑤ ⑥⑥ ⑦⑦ ⑧⑧ ⑨⑨	⓪⓪ ①① ②② ③③ ④④ ⑤⑤ ⑥⑥ ⑦⑦ ⑧⑧ ⑨⑨

5. Have you ever had a Kidney, Ureter, Bladder (KUB) X-ray?

◯ Yes ◯ No ◯ Don't Know

Approximate No. of times	Approximate Yr. first done
[ ][ ]	19 [ ][ ]
⓪⓪ ①① ②② ③③ ④④ ⑤⑤ ⑥⑥ ⑦⑦ ⑧⑧ ⑨⑨	⓪⓪ ①① ②② ③③ ④④ ⑤⑤ ⑥⑥ ⑦⑦ ⑧⑧ ⑨⑨

6. Have you ever had a Urethrogram?

◯ Yes ◯ No ◯ Don't Know

Approximate No. of times	Approximate Yr. first done
[ ][ ]	19 [ ][ ]
⓪⓪ ①① ②② ③③ ④④ ⑤⑤ ⑥⑥ ⑦⑦ ⑧⑧ ⑨⑨	⓪⓪ ①① ②② ③③ ④④ ⑤⑤ ⑥⑥ ⑦⑦ ⑧⑧ ⑨⑨

43. Continued

7. Have you ever undergone Cystography?

○ Yes ○ No ○ Don't Know

Approximate No. of times		Approximate Yr. first done	
		19	
0	0	0	0
1	1	1	1
2	2	2	2
3	3	3	3
4	4	4	4
5	5	5	5
6	6	6	6
7	7	7	7
8	8	8	8
9	9	9	9

8. Have you ever had an Upper Gastro-Intestinal Tract series?

○ Yes ○ No ○ Don't Know

Approximate No. of times		Approximate Yr. first done	
		19	
0	0	0	0
1	1	1	1
2	2	2	2
3	3	3	3
4	4	4	4
5	5	5	5
6	6	6	6
7	7	7	7
8	8	8	8
9	9	9	9

9. Have you ever had a Barium Swallow?

○ Yes ○ No ○ Don't Know

Approximate No. of times		Approximate Yr. first done	
		19	
0	0	0	0
1	1	1	1
2	2	2	2
3	3	3	3
4	4	4	4
5	5	5	5
6	6	6	6
7	7	7	7
8	8	8	8
9	9	9	9

10. Have you ever had a Mammogram of the Breast?

○ Yes ○ No ○ Don't Know

Approximate No. of times		Approximate Yr. first done	
		19	
0	0	0	0
1	1	1	1
2	2	2	2
3	3	3	3
4	4	4	4
5	5	5	5
6	6	6	6
7	7	7	7
8	8	8	8
9	9	9	9

11. Have you ever undergone Angiography?

○ Yes ○ No ○ Don't Know

Approximate No. of times		Approximate Yr. first done	
		19	
0	0	0	0
1	1	1	1
2	2	2	2
3	3	3	3
4	4	4	4
5	5	5	5
6	6	6	6
7	7	7	7
8	8	8	8
9	9	9	9

12. Other special X-ray procedures (please specify →)

Approximate No. of times		Approximate Yr. first done	
		19	
0	0	0	0
1	1	1	1
2	2	2	2
3	3	3	3
4	4	4	4
5	5	5	5
6	6	6	6
7	7	7	7
8	8	8	8
9	9	9	9

44a. We are also interested in DIAGNOSTIC X-RAYS you have had, other than those special X-rays listed previously. Please indicate whether or not each part of the body listed has ever been x-rayed. For each "Yes" you mark, please indicate (b) the APPROXIMATE number of times you had that part of the body x-rayed and (c) the APPROXIMATE year you first had that part of your body x-rayed.

Part of body — Head and Neck

1. Skull — ever x-rayed?

○ Yes ○ No ○ Don't Know

Approximate No. of times		Approximate Yr. first done	
		19	
0	0	0	0
1	1	1	1
2	2	2	2
3	3	3	3
4	4	4	4
5	5	5	5
6	6	6	6
7	7	7	7
8	8	8	8
9	9	9	9

2. Dental — ever x-rayed?

○ Yes ○ No ○ Don't Know

Approximate No. of times		Approximate Yr. first done	
		19	
0	0	0	0
1	1	1	1
2	2	2	2
3	3	3	3
4	4	4	4
5	5	5	5
6	6	6	6
7	7	7	7
8	8	8	8
9	9	9	9

3. Cervical spine — ever x-rayed?

○ Yes ○ No ○ Don't Know

Approximate No. of times		Approximate Yr. first done	
		19	
0	0	0	0
1	1	1	1
2	2	2	2
3	3	3	3
4	4	4	4
5	5	5	5
6	6	6	6
7	7	7	7
8	8	8	8
9	9	9	9

4. Other head and neck — ever x-rayed?

○ Yes ○ No ○ Don't Know

Approximate No. of times		Approximate Yr. first done	
		19	
0	0	0	0
1	1	1	1
2	2	2	2
3	3	3	3
4	4	4	4
5	5	5	5
6	6	6	6
7	7	7	7
8	8	8	8
9	9	9	9

Part of body — Trunk

5. Chest — ever x-rayed?

○ Yes ○ No ○ Don't Know

Approximate No. of times		Approximate Yr. first done	
		19	
0	0	0	0
1	1	1	1
2	2	2	2
3	3	3	3
4	4	4	4
5	5	5	5
6	6	6	6
7	7	7	7
8	8	8	8
9	9	9	9

6. Collar bone — ever x-rayed?

○ Yes ○ No ○ Don't Know

Approximate No. of times		Approximate Yr. first done	
		19	
0	0	0	0
1	1	1	1
2	2	2	2
3	3	3	3
4	4	4	4
5	5	5	5
6	6	6	6
7	7	7	7
8	8	8	8
9	9	9	9

44. Continued

7. Shoulder — ever x-rayed?

- ◯ Yes
- ◯ No
- ◯ Don't Know

Approximate No. of times			Approximate Yr. first done	
		19		
0	0		0	0
1	1		1	1
2	2		2	2
3	3		3	3
4	4		4	4
5	5		5	5
6	6		6	6
7	7		7	7
8	8		8	8
9	9		9	9

8. Ribs — ever x-rayed?

- ◯ Yes
- ◯ No
- ◯ Don't Know

Approximate No. of times			Approximate Yr. first done	
		19		
0	0		0	0
1	1		1	1
2	2		2	2
3	3		3	3
4	4		4	4
5	5		5	5
6	6		6	6
7	7		7	7
8	8		8	8
9	9		9	9

13. Pelvis — ever x-rayed?

- ◯ Yes
- ◯ No
- ◯ Don't Know

Approximate No. of times			Approximate Yr. first done	
		19		
0	0		0	0
1	1		1	1
2	2		2	2
3	3		3	3
4	4		4	4
5	5		5	5
6	6		6	6
7	7		7	7
8	8		8	8
9	9		9	9

14. Other (please specify)

Approximate No. of times			Approximate Yr. first done	
		19		
0	0		0	0
1	1		1	1
2	2		2	2
3	3		3	3
4	4		4	4
5	5		5	5
6	6		6	6
7	7		7	7
8	8		8	8
9	9		9	9

9. Abdomen — ever x-rayed?

- ◯ Yes
- ◯ No
- ◯ Don't Know

Approximate No. of times			Approximate Yr. first done	
		19		
0	0		0	0
1	1		1	1
2	2		2	2
3	3		3	3
4	4		4	4
5	5		5	5
6	6		6	6
7	7		7	7
8	8		8	8
9	9		9	9

10. Thoracic spine — ever x-rayed?

- ◯ Yes
- ◯ No
- ◯ Don't Know

Approximate No. of times			Approximate Yr. first done	
		19		
0	0		0	0
1	1		1	1
2	2		2	2
3	3		3	3
4	4		4	4
5	5		5	5
6	6		6	6
7	7		7	7
8	8		8	8
9	9		9	9

44b. Have you ever personally undergone any THERAPEUTIC X-RAY PROCEDURES? Here we are interested in procedures performed on you, not those performed by you.

- ◯ Yes
- ◯ No
- ◯ Don't Know

IF YOU MARKED NO OR DON'T KNOW, SKIP TO QUESTION 45.

If you marked yes, please mark the body site(s) treated with X-rays and the year treated, and list the reasons for the therapy on page 12.

11. Lumbar spine — ever x-rayed?

- ◯ Yes
- ◯ No
- ◯ Don't Know

Approximate No. of times			Approximate Yr. first done	
		19		
0	0		0	0
1	1		1	1
2	2		2	2
3	3		3	3
4	4		4	4
5	5		5	5
6	6		6	6
7	7		7	7
8	8		8	8
9	9		9	9

12. Lumbosacral spine — ever x-rayed?

- ◯ Yes
- ◯ No
- ◯ Don't Know

Approximate No. of times			Approximate Yr. first done	
		19		
0	0		0	0
1	1		1	1
2	2		2	2
3	3		3	3
4	4		4	4
5	5		5	5
6	6		6	6
7	7		7	7
8	8		8	8
9	9		9	9

1. Head and neck

Year first treated

19		
	0	0
	1	1
	2	2
	3	3
	4	4
	5	5
	6	6
	7	7
	8	8
	9	9

2. Pelvis

Year first treated

19		
	0	0
	1	1
	2	2
	3	3
	4	4
	5	5
	6	6
	7	7
	8	8
	9	9

44b. (Continued) Continue to mark the body site(s) treated with X-rays and the year treated.

3. Extremities

Year first treated

19 ☐☐

⓪⓪ ①① ②② ③③ ④④ ⑤⑤ ⑥⑥ ⑦⑦ ⑧⑧ ⑨⑨

4. Chest

Year first treated

19 ☐☐

⓪⓪ ①① ②② ③③ ④④ ⑤⑤ ⑥⑥ ⑦⑦ ⑧⑧ ⑨⑨

5. Other body sites (please specify sites)

Please Print — Stay within box

Year first treated

19 ☐☐

⓪⓪ ①① ②② ③③ ④④ ⑤⑤ ⑥⑥ ⑦⑦ ⑧⑧ ⑨⑨

Reason for therapeutic X-rays listed above in questions 1-5.

Please print — Stay within box

45. Have you personally ever undergone any diagnostic or therapeutic radioisotope procedures? Here we are interested in procedures performed on you, not those performed by you.

◯ Yes ◯ No ◯ Don't know

IF YOU MARKED NO OR DON'T KNOW, SKIP TO QUESTION 47.

46. Has any part of your body ever been treated or diagnosed with radioisotopes? Please mark (a) "Yes" or "No" for each part of the body indicating whether or not you had been treated or diagnosed with radioisotopes. For each "Yes" indicate (b) the purpose of the radioisotope procedure, (c) the type of isotope used, (d) the number of times you had the procedure and (e) the year the procedure was done for the first time.

1. Thyroid — treated or diagnosed with radioisotopes?

a. ◯ Yes ◯ No

b. ◯ Therapeutic ◯ Diagnostic ◯ Both

c. Type of isotope

◯ ^{131}I

◯ ^{99m}Tc

◯ Other (please specify)

d. No. of times ☐☐

⓪⓪ ①① ②② ③③ ④④ ⑤⑤ ⑥⑥ ⑦⑦ ⑧⑧ ⑨⑨

e. Yr. first done 19 ☐☐

⓪⓪ ①① ②② ③③ ④④ ⑤⑤ ⑥⑥ ⑦⑦ ⑧⑧ ⑨⑨

2. Sites other than the thyroid treated or diagnosed with radioisotopes?

a. ◯ Yes ◯ No

If yes, please specify site:

b. ◯ Therapeutic ◯ Diagnostic ◯ Both

c. Type of isotope

◯ ^{99m}Tc

◯ ^{131}I

◯ ^{198}Au

◯ ^{197}Hg

◯ ^{203}Hg

◯ Other (please specify)

d. No. of times ☐☐

⓪⓪ ①① ②② ③③ ④④ ⑤⑤ ⑥⑥ ⑦⑦ ⑧⑧ ⑨⑨

e. Yr. first done 19 ☐☐

⓪⓪ ①① ②② ③③ ④④ ⑤⑤ ⑥⑥ ⑦⑦ ⑧⑧ ⑨⑨

47. When you were training to be a radiologic technologist or radiologic technician, did other students ever practice taking X-rays on you?

◯ Yes ◯ No

48. If yes, how many times?

◯ 1-9 ◯ 10-24 ◯ 25-49 ◯ 50+

49. Have you or your wife (wives) ever been pregnant? (Count live births, stillbirths, miscarriages, and abortions.)

◯ Yes
◯ No

IF YOU MARKED NO, MALES SKIP TO QUESTION 73, FEMALES SKIP TO QUESTION 54.

50. How many times have you or your wife (wives) been pregnant?

TIMES

0 1 2 3 4 5 6 7 8 9 | 0 1 2 3 4 5 6 7 8 9

51. How many live births have you or your wife (wives) had (count twins as 1 birth)?

LIVE BIRTHS

0 1 2 3 4 5 6 7 8 9 | 0 1 2 3 4 5 6 7 8 9

52. How many miscarriages have you or your wife (wives) had?

0 1 2 3 4 5 6 7 8 9 | 0 1 2 3 4 5 6 7 8 9

53. How many children were carried to term but stillborn?

0 1 2 3 4 5 6 7 8 9 | 0 1 2 3 4 5 6 7 8 9

III. GYNECOLOGICAL HISTORY FEMALES ONLY Males Skip to Question 73

54. Did you ever or do you now take birth control pills?

◯ Yes ◯ No

IF YOU MARKED NO, SKIP TO QUESTION 58.

55. Are you currently taking birth control pills?

◯ Yes ◯ No

56. How old were you when you first took birth control pills?

0 1 2 3 4 5 6 7 8 9 | 0 1 2 3 4 5 6 7 8 9

57. Altogether, what was the total number of years you used birth control pills?

0 1 2 3 4 5 6 7 8 9 | 0 1 2 3 4 5 6 7 8 9

58. How old were you when your first menstrual period started?

0 1 2 3 4 5 6 7 8 9 | 0 1 2 3 4 5 6 7 8 9

59. Have your menstrual periods stopped completely?

◯ Yes
◯ No

IF YOU MARKED NO, SKIP TO QUESTION 63.

60. How old were you when your periods stopped completely?

0 1 2 3 4 5 6 7 8 9 | 0 1 2 3 4 5 6 7 8 9

61. What was the reason your periods stopped?

- ◯ Because of surgery (hysterectomy)
- ◯ Natural menopause (change of life)
- ◯ Because of radiation treatment I received
- ◯ Another reason (describe below)

IF YOU MARKED SURGERY, PLEASE ANSWER QUESTION 62. ALL OTHERS, SKIP TO QUESTION 63.

62. If surgery, how many ovaries were removed?

- ◯ Both
- ◯ One
- ◯ None
- ◯ Don't know

63. Some people take hormone pills for hot flashes or mood changes during menopause or because periods have stopped due to an operation. Did you ever take hormone pills for any reasons related to menopause?

- ◯ Yes
- ◯ No

IF YOU MARKED NO, SKIP TO QUESTION 68.

64. If you took hormone pills for a reason related to menopause, please mark the reason below.

- ◯ Hot flashes
- ◯ Mood changes
- ◯ Because periods had stopped
- ◯ Don't know reason

65. How old were you when you first took hormone pills for a reason related to menopause?

YEARS OLD

0	0
1	1
2	2
3	3
4	4
5	5
6	6
7	7
8	8
9	9

66. Altogether, how long did you take hormone pills for this reason?

YEARS

0	0
1	1
2	2
3	3
4	4
5	5
6	6
7	7
8	8
9	9

MONTHS

- ◯ 1
- ◯ 2
- ◯ 3
- ◯ 4
- ◯ 5
- ◯ 6
- ◯ 7
- ◯ 8
- ◯ 9
- ◯ 10
- ◯ 11

67. Are you currently taking hormone pills for this reason?

- ◯ Yes
- ◯ No

68. Have you ever had a breast biopsy?

- ◯ Yes
- ◯ No

IF YOU MARKED NO, SKIP TO QUESTION 71.

69. What year was your first breast biopsy?

19

0	0
1	1
2	2
3	3
4	4
5	5
6	6
7	7
8	8
9	9

70. How many breast biopsies have you had?

NUMBER

0	0
1	1
2	2
3	3
4	4
5	5
6	6
7	7
8	8
9	9

71. Have any members of your immediate family, that is, grandmother, mother, aunt, sister, or daughter, had breast cancer?

- ◯ Yes
- ◯ No

IF YOU MARKED NO, SKIP TO QUESTION 73.

72. Mark all those relatives who have had breast cancer.

- ◯ Grandmother
- ◯ Mother
- ◯ Sister
- ◯ Daughter
- ◯ Maternal Aunt
- ◯ Paternal Aunt

IV. CURRENT ADDRESS INFORMATION

73. We are requesting your Social Security Number because it would be helpful in locating your whereabouts if we wished to contact you in a few years. Disclosure of your Social Security Number is voluntary. Refusing to provide your Social Security Number will in no way affect any rights, benefits or privileges which you may now or in the future be receiving.

My Social Security Number is: ___ ___ ___ – ___ ___ – ___ ___ ___ ___

0	0	0	0	0	0	0	0	0
1	1	1	1	1	1	1	1	1
2	2	2	2	2	2	2	2	2
3	3	3	3	3	3	3	3	3
4	4	4	4	4	4	4	4	4
5	5	5	5	5	5	5	5	5
6	6	6	6	6	6	6	6	6
7	7	7	7	7	7	7	7	7
8	8	8	8	8	8	8	8	8
9	9	9	9	9	9	9	9	9

○ I don't have a Social Security Number.

74. It would also be helpful if you could provide us with the name and address of someone who could give us your new address should you move. We would contact this person only if we are unable to reach you at your home address.

Name: ____
First | Last | Relationship to you

Address: ____
Street

____
City | State | ZIP Code

75. Please sign the consent form on the back of this questionnaire.

Please use this space for any additional comments or information.

____

____

____

____

____

____

____

____

University of Minnesota
American Registry of Radiologic Technologists
National Institutes of Health Collaborative Health Study

AUTHORIZATION TO OBTAIN MEDICAL and/or DOSIMETRY RECORDS

I hereby voluntarily authorize the study investigators at the University of Minnesota to request and obtain my medical records from my physican or from a hospital where I have been seen and to request and obtain dosimetry information from previous or current employers.

I understand that:

1) the information obtained from medical records will be used to evaluate the possible health effects of occupational radiation exposures.

2) the survey is authorized by the general provisions of the Public Health Service Act.

3) the information obtained from any of my records will be kept strictly confidential.

4) neither my name nor any other identifying information will ever appear in any report of this survey.

5) I may withdraw this consent at any time without prejudice.

This authorization is valid for a period of one year from the date of signature.

____
Signature

____
Date

Please check each page carefully to make certain you have answered all questions that apply to you. Pay particular attention to the tables, then mail back the questionnaire in the enclosed envelope.

THANK YOU VERY MUCH FOR YOUR COOPERATION.

OMB 0925-0405
Expires: 08/31/96

University of Minnesota
American Registry of Radiologic Technologists
National Institutes of Health

If you have any questions, please call or write:

Health Studies Section
University of Minnesota
School of Public Health
Box 807 UMHC
420 Delaware St. SE
Minneapolis, MN 55455
(612) 625-1151

Please use a No. 2. lead pencil when completing this form.

0	0	0	0	0	0		0
1	1	1	1	1	1		1
2	2	2	2	2	2		2
3	3	3	3	3	3		3
4	4	4	4	4	4		4
5	5	5	5	5	5		5
6	6	6	6	6	6		6
7	7	7	7	7	7		7
8	8	8	8	8	8		8
9	9	9	9	9	9		9

Public reporting burden for this collection of information is estimated to average 20 minutes per response, including the time for reviewing instructions, searching existing data sources, gathering and maintaining the data needed, and completing and reviewing the collection of information. Send comments regarding this burden estimate or any other aspect of this collection of information to: PHS Reports Clearance Officers, Rm. 721-B, Humphrey Building, 200 Independence Ave. SW, Washington, D.C. 20201. ATTN: PRA; and to Office of Management and Budget Paperwork Reduction Project (0925-0405), Washington, D.C. 20503.

Instructions for completing questionnaire:

Please be sure to mark a response for every question, unless you have been instructed to skip a question.

Your responses will be read by an optical reader. It is important to follow the instructions below when recording your answers.

- Use black lead pencil only (No. 2 or softer).
- Do NOT use ink or ballpoint pens.
- Make heavy black marks that fill the circle completely.
- Erase cleanly any answer you wish to change.
- Make no stray marks on the answer sheet.

EXAMPLES

Proper Mark ○●○ | Improper Marks

EXAMPLES

To record a year: (example 1916) — 19 | 1 | 6

To record a two digit number: (example 27) — 2 | 7

To record a one digit number: (example 3) — 0 | 3

A. GENERAL INFORMATION

1. Please record TODAY'S MONTH AND YEAR.

Month	
○ Jan	○ Jul
○ Feb	○ Aug
○ Mar	○ Sep
○ Apr	○ Oct
○ May	○ Nov
○ Jun	○ Dec

Year: 199_ (4, 5, 6, 7, 8, 9)

2. What is your BIRTH DATE?

Month
○ Jan
○ Feb
○ Mar
○ Apr
○ May
○ Jun
○ Jul
○ Aug
○ Sep
○ Oct
○ Nov
○ Dec

Day: __ __

Year: 1_ _ _

3. What is your SEX?
- ○ Male
- ○ Female

4. What is your CURRENT MARITAL STATUS?
- ○ Never married
- ○ Married
- ○ Living together but not married
- ○ Divorced
- ○ Widowed
- ○ Separated

5. Were you or are you now married to a medical radiation worker?
- ○ No
- ○ Yes

6. Which of these categories best describes you?
- ○ White
- ○ Black
- ○ Asian or Pacific Islander
- ○ American Indian or Alaskan Native
- ○ Other, specify ______

7. Are you of Hispanic origin?
- ○ No
- ○ Yes

8. In what RELIGION were you raised?
- ◯ None
- ◯ Assembly of God
- ◯ Baptist
- ◯ Christian
- ◯ Christian Science
- ◯ Church of Christ
- ◯ Congregational
- ◯ Episcopal
- ◯ Jehovah's Witness
- ◯ Jewish
- ◯ Lutheran
- ◯ Methodist
- ◯ Mormon or Latter Day Saint
- ◯ Pentecostal
- ◯ Presbyterian
- ◯ Roman Catholic
- ◯ Seventh Day Adventist
- ◯ Unitarian Universalist
- ◯ Other, specify____________________

9. About how TALL are you without shoes?

FEET: ⓪ ① ② ③ ④ ⑤ ⑥ ⑦

INCHES: ⓪ ① / ⓪ ① ② ③ ④ ⑤ ⑥ ⑦ ⑧ ⑨

10. About how much do you usually WEIGH without clothes or shoes?

POUNDS: ⓪–⑥ / ⓪–⑨ / ⓪–⑨

11. Have you ever SMOKED cigarettes regularly for a period of one year or more?
- ◯ No (GO TO QUESTION 16, PAGE 4)
- ◯ Yes

12. Excluding any periods of time during which you did not smoke, how many total years have you smoked regularly?

TOTAL YEARS SMOKED: ⓪–⑨ / ⓪–⑨

13. During the time you smoked regularly, about how many cigarettes per day did you smoke?

NO. OF CIGARETTES PER DAY: ⓪–⑨ / ⓪–⑨

14. How old were you when you started smoking regularly?

AGE STARTED: ⓪–⑨ / ⓪–⑨

15. How old were you when you stopped smoking?

◯ Currently smoking (GO TO QUESTION 16, PAGE 4)

AGE STOPPED: ⓪–⑨ / ⓪–⑨

B. WORKING EXPERIENCE

In this section we are interested in your exposure to radiation while TRAINING and/or WORKING with medical radiation, such as x-ray, radionuclide, radiotherapy, ultra sound or MRI procedures.

16. In what years did you begin TRAINING and WORKING with medical radiation procedures?

◯ Never trained ◯ Never worked

YEAR BEGAN TRAINING	YEAR BEGAN WORKING
19__	19__

(0 1 2 3 4 5 6 7 8 9 bubbles for each of the last two digits)

17. In total, how many years have you TRAINED and/or WORKED with medical radiation procedures? Exclude years when you were not personally working or assisting with medical radiation procedures for patient care. If none, record "00" and go to Question 28 on Page 6.

(Record information about other occupational radiation exposures in comments section on back page of questionnaire).

NUMBER OF YEARS: __ __ (0 1 2 3 4 5 6 7 8 9 bubbles for each digit)

18. For each of the following time periods, please indicate how many years you TRAINED and/or WORKED with medical radiation procedures. If none, record "00".

NUMBER OF YEARS

Before 1950	1950's	1960's	1970's	1980's	1990 or later
					0

(Bubbles: Before 1950 — first digit 0–4, second digit 0–9; 1950's to 1980's — first digit 0–1, second digit 0–9; 1990 or later — first digit 0 (filled), second digit 0–9)

19. In what year did you stop TRAINING and/or WORKING with medical radiation procedures?

◯ Currently working (GO TO QUESTION 20)

YEAR STOPPED: 19__ (0 1 2 3 4 5 6 7 8 9 bubbles for each of the last two digits)

20. What is your LIFETIME TOTAL RADIATION EXPOSURE received while working in the field of medical radiation? (1000 mrad = 1 rad = 1 rem = 1 cGy = 10 mSv)

- ◯ None (GO TO QUESTION 22)
- ◯ < 1,000 mrad
- ◯ 1,000 - 4,999 mrad
- ◯ 5,000 - 9,999 mrad
- ◯ 10,000 - 24,999 mrad
- ◯ 25,000 - 49,999 mrad
- ◯ ≥ 50,000 mrad
- ◯ Don't know (GO TO QUESTION 22)

21. Is your answer estimated or taken from your dosimetry reports?

- ◯ Estimated
- ◯ From dosimetry reports
- ◯ Combination of both

22. While working in the field of medical radiation, about how many times were you removed from receiving additional radiation exposure for any length of time because you reached your exposure limit?

- ◯ None
- ◯ 1
- ◯ 2 - 4
- ◯ 5 - 9
- ◯ 10+
- ◯ Don't know

23. About how many times has your WHITE BLOOD CELL COUNT been depressed below normal as a result of working in the field of medical radiation?

- ◯ None
- ◯ 1
- ◯ 2 - 4
- ◯ 5 - 9
- ◯ 10+
- ◯ Don't know
- ◯ Never tested

24. Please indicate how frequently you worked or assisted on a regular basis with each of the following procedures during the specified calendar years. If you never worked with a particular procedure, mark the circle for "Never worked with" and leave all other columns blank for that procedure.

PROCEDURES YOU WORKED WITH	Never worked with	BEFORE 1980 FREQUENCY				1980 - 1989 FREQUENCY				1990 OR LATER FREQUENCY			
		Never or rarely	Monthly	Weekly	Daily	Never or rarely	Monthly	Weekly	Daily	Never or rarely	Monthly	Weekly	Daily
Fluoroscopy	○	○	○	○	○	○	○	○	○	○	○	○	○
Dental X-ray	○	○	○	○	○	○	○	○	○	○	○	○	○
Routine Diagnostic X-rays	○	○	○	○	○	○	○	○	○	○	○	○	○
Multi-film Procedures (e.g., IVP)	○	○	○	○	○	○	○	○	○	○	○	○	○
Other Angiography	○	○	○	○	○	○	○	○	○	○	○	○	○
Portable X-ray	○	○	○	○	○	○	○	○	○	○	○	○	○
CAT or CT Scan	○	○	○	○	○	○	○	○	○	○	○	○	○
Interventional Radiography	○	○	○	○	○	○	○	○	○	○	○	○	○
Diagnostic Radionuclide	○	○	○	○	○	○	○	○	○	○	○	○	○
Diagnostic Ultrasound	○	○	○	○	○	○	○	○	○	○	○	○	○
External Beam Therapy (MeV)	○	○	○	○	○	○	○	○	○	○	○	○	○
Orthovoltage Therapy	○	○	○	○	○	○	○	○	○	○	○	○	○
Brachytherapy (radium or other)	○	○	○	○	○	○	○	○	○	○	○	○	○
Radioactive Iodine Therapy	○	○	○	○	○	○	○	○	○	○	○	○	○
Other Radionuclide Therapy	○	○	○	○	○	○	○	○	○	○	○	○	○
Microwave or Ultrasound Diathermy	○	○	○	○	○	○	○	○	○	○	○	○	○
Mammography	○	○	○	○	○	○	○	○	○	○	○	○	○
MRI, Magnetic Reasonance Imaging	○	○	○	○	○	○	○	○	○	○	○	○	○
Any other procedures, specify: ____________	○	○	○	○	○	○	○	○	○	○	○	○	○

25. Please indicate how frequently you HELD PATIENTS during any x-ray, radionuclide, or radiotherapy procedures listed above during the specified calendar years. If you never held a patient during any procedure, mark the circle for "Never held" and leave all other columns blank.

HELD PATIENTS	Never held	BEFORE 1980 FREQUENCY				1980 - 1989 FREQUENCY				1990 OR LATER FREQUENCY			
		Never or rarely	Monthly	Weekly	Daily	Never or rarely	Monthly	Weekly	Daily	Never or rarely	Monthly	Weekly	Daily
	○	○	○	○	○	○	○	○	○	○	○	○	○

26. Please indicate how often you wore a LEAD APRON or stood behind a LEAD SHIELD while working or assisting with any procedure listed above during the specified calendar years. If you never used a lead apron or shield, mark the circle for "Never used" and leave all other columns blank.

USED LEAD APRON OR SHIELD	Never used	BEFORE 1980 FREQUENCY				1980 - 1989 FREQUENCY				1990 OR LATER FREQUENCY			
		Never or rarely	Monthly	Weekly	Daily	Never or rarely	Monthly	Weekly	Daily	Never or rarely	Monthly	Weekly	Daily
	○	○	○	○	○	○	○	○	○	○	○	○	○

27. Please indicate how often you developed or processed X-RAY FILM during the specified calendar periods. If you never developed or processed x-ray film, mark the circle for "Never processed" and leave all other columns blank.

X-RAY FILM PROCESSING	Never processed	BEFORE 1980 FREQUENCY				1980 - 1989 FREQUENCY				1990 OR LATER FREQUENCY			
		Never or rarely	Monthly	Weekly	Daily	Never or rarely	Monthly	Weekly	Daily	Never or rarely	Monthly	Weekly	Daily
	○	○	○	○	○	○	○	○	○	○	○	○	○

C. PERSONAL MEDICAL EXPOSURES

In this section we are interested in radiation exposure YOU RECEIVED AS A PATIENT, NOT procedures performed BY YOU.

28. Please indicate how frequently you had any of the following DIAGNOSTIC PROCEDURES during the specified calendar years. If you never had a particular procedure, mark the circle for "Never had" and leave all other columns blank for that procedure. Please count the number of times you had a procedure, NOT the number of individual films taken.

○ I have not had any of the procedures listed below (GO TO QUESTION 29)

PROCEDURES PERFORMED ON YOU	NEVER HAD	BEFORE 1980 NUMBER TIMES				1980 - 1989 NUMBER TIMES				1990 OR LATER NUMBER TIMES			
		0	1	2 - 4	5+	0	1	2 - 4	5+	0	1	2 - 4	5+
Upper gastrointestinal series	○	○	○	○	○	○	○	○	○	○	○	○	○
Barium swallow	○	○	○	○	○	○	○	○	○	○	○	○	○
Barium enema	○	○	○	○	○	○	○	○	○	○	○	○	○
Cholecystogram or cholangiogram	○	○	○	○	○	○	○	○	○	○	○	○	○
Retrograde or intravenous pyelogram	○	○	○	○	○	○	○	○	○	○	○	○	○
Renal arteriogram	○	○	○	○	○	○	○	○	○	○	○	○	○
Urethrogram or cystogram	○	○	○	○	○	○	○	○	○	○	○	○	○
Multi-film procedure, other than above	○	○	○	○	○	○	○	○	○	○	○	○	○
Angiogram, other than above	○	○	○	○	○	○	○	○	○	○	○	○	○
Fluoroscopic procedure, other than above	○	○	○	○	○	○	○	○	○	○	○	○	○
CAT or CT scan	○	○	○	○	○	○	○	○	○	○	○	○	○
MRI procedure	○	○	○	○	○	○	○	○	○	○	○	○	○
Skull x-ray	○	○	○	○	○	○	○	○	○	○	○	○	○
Cervical spine x-ray	○	○	○	○	○	○	○	○	○	○	○	○	○
Sinus x-ray	○	○	○	○	○	○	○	○	○	○	○	○	○
Head/neck x-ray, other than above	○	○	○	○	○	○	○	○	○	○	○	○	○
Collar bone x-ray	○	○	○	○	○	○	○	○	○	○	○	○	○
Shoulder x-ray	○	○	○	○	○	○	○	○	○	○	○	○	○
Rib x-ray	○	○	○	○	○	○	○	○	○	○	○	○	○
Thoracic spine x-ray	○	○	○	○	○	○	○	○	○	○	○	○	○
Lumbar spine x-ray	○	○	○	○	○	○	○	○	○	○	○	○	○
Lumbosacral spine x-ray	○	○	○	○	○	○	○	○	○	○	○	○	○
Abdomen x-ray	○	○	○	○	○	○	○	○	○	○	○	○	○
Kidney/ureter/bladder x-ray	○	○	○	○	○	○	○	○	○	○	○	○	○
Pelvis x-ray	○	○	○	○	○	○	○	○	○	○	○	○	○
Extremities (arms, legs) x-ray	○	○	○	○	○	○	○	○	○	○	○	○	○
Mammography x-ray	○	○	○	○	○	○	○	○	○	○	○	○	○

29. Please indicate how frequently you had DENTAL OR CHEST X-RAYS during the specified calendar years. If you never had a particular procedure, mark the circle for "Never had" and leave all other columns blank for that procedure. Please count the number of times you had a procedure, NOT the number of individual films taken.

PROCEDURES PERFORMED ON YOU	NEVER HAD	BEFORE 1980 NUMBER TIMES				1980 - 1989 NUMBER TIMES				1990 OR LATER NUMBER TIMES			
		0	1 - 9	10 - 24	25+	0	1 - 9	10 - 24	25+	0	1 - 9	10 - 24	25+
Dental x-ray	○	○	○	○	○	○	○	○	○	○	○	○	○
Chest x-ray	○	○	○	○	○	○	○	○	○	○	○	○	○

30. **As a PATIENT, have you undergone any RADIOTHERAPY procedures, including radium implants or other brachytherapy? Here we are interested in procedures performed ON YOU, not those performed BY YOU.**

○ No (GO TO QUESTION 31)

○ Yes → Please indicate whether you received radiotherapy to any of the following body areas for cancer or any other condition during the specified calendar years. If you did not receive radiotherapy during a specific time period, leave items under that column blank. If you never received radiotherapy to a particular body area, mark the circle for "Never received" and leave all other columns blank for that body area.

BODY AREA TREATED WITH RADIOTHERAPY	Never Received	Before 1980		1980 - 1989		1990+	
		For Cancer	Not for Cancer	For Cancer	Not for Cancer	For Cancer	Not for Cancer
Head or neck	○	○	○	○	○	○	○
Shoulder	○	○	○	○	○	○	○
Chest or spine	○	○	○	○	○	○	○
Abdomen	○	○	○	○	○	○	○
Pelvis	○	○	○	○	○	○	○
Extremities	○	○	○	○	○	○	○
Other, specify: ______	○	○	○	○	○	○	○

31. **As a PATIENT, have you undergone any NUCLEAR MEDICINE procedures? Here we are interested in procedures performed ON YOU, not those performed BY YOU.**

○ No (GO TO QUESTION 32)

○ Yes → Please indicate whether you received any of the following radionuclides for a diagnostic or therapeutic reason during the specified calendar years. If you did not receive radionuclides during a specific time period, leave items under that column blank. If you never received a particular radionuclide, mark the circle for "never received" and leave all other columns blank for that radionuclide.

TYPE OF RADIONUCLIDE	Never Received	Before 1980		1980 - 1989		1990+	
		For Diagnosis	For Therapy	For Diagnosis	For Therapy	For Diagnosis	For Therapy
^{131} Iodine	○	○	○	○	○	○	○
^{125} Iodine	○	○	○	○	○	○	○
^{99m} Technecium	○	○	○	○	○	○	○
^{201} Thallium	○	○	○	○	○	○	○
Other, specify: ______	○	○	○	○	○	○	○
Type unknown	○	○	○	○	○	○	○

D. HISTORY OF ILLNESS

32. **Have you ever been told by a doctor that you had any type of CANCER?**

○ No (GO TO QUESTION 33, PAGE 8)

○ Yes → Please mark "YES" for each type of cancer you have had diagnosed by a doctor and specify the year of first diagnosis. Include only primary cancers, not those that originated from a different site (i.e., do not list metastases).

PRIMARY CANCER	Mark here for "Yes"	YEAR OF FIRST DIAGNOSIS
Bladder	○ →	19____
Bone	○ →	19____
Brain or Central Nervous System	○ →	19____
Breast	○ →	19____
Cervix (invasive)	○ →	19____
Colon	○ →	19____
ConnectiveTissue (Soft Tissue Sarcoma)	○ →	19____
Esophagus	○ →	19____
Hodgkin's Disease	○ →	19____
Kaposi's Sarcoma	○ →	19____
Kidney	○ →	19____
Larynx	○ →	19____
Leukemia, Acute Lymphocytic	○ →	19____
Leukemia, Chronic Lymphocytic	○ →	19____
Leukemia, Acute Myeloid or Granulocytic	○ →	19____
Leukemia, Chronic Myeloid or Granulocytic	○ →	19____
Leukemia, other than above or type unknown	○ →	19____
Liver	○ →	19____
Lung or Bronchus	○ →	19____
Lymphoma, Non-Hodgkin's	○ →	19____
Lymphoma, type unknown	○ →	19____
Melanoma	○ →	19____
Multiple Myeloma	○ →	19____
Oral Cavity or Pharynx	○ →	19____
Ovary	○ →	19____
Pancreas	○ →	19____
Prostate	○ →	19____
Rectum	○ →	19____
Stomach	○ →	19____
Testis	○ →	19____
Thyroid	○ →	19____
Uterus (endometrium)	○ →	19____
Skin, Basal Cell Carcinoma	○ →	19____
Skin, Squamous Cell Carcinoma	○ →	19____
Other cancer, specify below	○ →	19____

____

33. Have you had any of the following conditions or procedures listed below diagnosed by a physician?

◯ No (FEMALES GO TO QUESTION 34; MALES GO TO QUESTION 53, PAGE 9)

◯ Yes → Please mark "YES" for each condition you've had diagnosed by a physician and indicate the time period when you were first diagnosed with that condition.

MEDICAL CONDITION	Mark here for "Yes"	YEAR OF FIRST DIAGNOSIS: <1980	1980 - 1984	1985 - 1989	1990+
Angina Pectoris	◯ →	◯	◯	◯	◯
Asthma	◯ →	◯	◯	◯	◯
Arthritis, Rheumatoid	◯ →	◯	◯	◯	◯
Arthritis, Other	◯ →	◯	◯	◯	◯
Breast Disease (Fibrocystic or Other Benign)	◯ →	◯	◯	◯	◯
Breast Implant, Silicone	◯ →	◯	◯	◯	◯
Bronchitis, Chronic	◯ →	◯	◯	◯	◯
Cataracts	◯ →	◯	◯	◯	◯
Cataract Extraction	◯ →	◯	◯	◯	◯
Cholecystectomy	◯ →	◯	◯	◯	◯
Cholesterol, Elevated (240 or greater)	◯ →	◯	◯	◯	◯
Coronary Bypass	◯ →	◯	◯	◯	◯
Diabetes Mellitus	◯ →	◯	◯	◯	◯
Emphysema	◯ →	◯	◯	◯	◯
Fracture of Hip or Forearm	◯ →	◯	◯	◯	◯
Glaucoma	◯ →	◯	◯	◯	◯
Hypertension (High Blood Pressure)	◯ →	◯	◯	◯	◯
Hip Replacement	◯ →	◯	◯	◯	◯
Macular Degeneration of Retina	◯ →	◯	◯	◯	◯
Myocardial Infarction (Heart Attack)	◯ →	◯	◯	◯	◯
Osteoporosis	◯ →	◯	◯	◯	◯
Pulmonary Embolus	◯ →	◯	◯	◯	◯
Scleroderma	◯ →	◯	◯	◯	◯
Stroke (CVA)	◯ →	◯	◯	◯	◯
Thyroid Conditions:					
Adenoma	◯ →	◯	◯	◯	◯
Goiter	◯ →	◯	◯	◯	◯
Hyperthyroidism	◯ →	◯	◯	◯	◯
Hypothyroidism	◯ →	◯	◯	◯	◯
Nodule	◯ →	◯	◯	◯	◯
Other benign thyroid condition, specify below ________	◯ →	◯	◯	◯	◯
Ulcer, Gastric or Duodenal	◯ →	◯	◯	◯	◯

E. FEMALE GYNECOLOGICAL HISTORY

(MALES GO TO QUESTION 53, PAGE 9)

34. How old were you when you first started having MENSTRUAL PERIODS?

AGE PERIODS STARTED: [ ][ ]
First digit: ⓪ ① ②
Second digit: ⓪ ① ② ③ ④ ⑤ ⑥ ⑦ ⑧ ⑨

35. Have your MENSTRUAL PERIODS stopped permanently?

◯ Yes, menstrual periods stopped.

◯ Had menopause, but now have periods due to hormone replacement therapy.

◯ No, still menstruating (GO TO QUESTION 39)

◯ Not sure (GO TO QUESTION 39)

36. How old were you when your natural MENSTRUAL PERIODS stopped?

AGE NATURAL PERIODS STOPPED: [ ][ ]
First digit: ⓪ ① ② ③ ④ ⑤ ⑥
Second digit: ⓪ ① ② ③ ④ ⑤ ⑥ ⑦ ⑧ ⑨

37. What is the reason your natural MENSTRUAL PERIODS stopped?

◯ Surgery → 38. How many ovaries were removed?
- ◯ Both
- ◯ One
- ◯ None
- ◯ Don't Know

◯ Natural menopause (change of life)

◯ Undernourishment

◯ Excessive exercise

◯ Other, specify ________

39. Have you ever taken BIRTH CONTROL PILLS (oral contraceptives) on a regular basis?

◯ No (GO TO QUESTION 41) ◯ Yes

40. Altogether, what was the total number of years you took BIRTH CONTROL PILLS (oral contraceptives) on a regular basis?

◯ <1 years
◯ 1 - 2 years
◯ 3 - 4 years
◯ 5 - 9 years
◯ 10+ years

41. Have you ever taken oral or other ESTROGENS for symptoms or conditions related to MENOPAUSE?

◯ No (GO TO QUESTION 46, PAGE 9) ◯ Yes

42. How old were you when you first took ESTROGENS for a reason related to MENOPAUSE?

AGE STARTED

0	0
1	1
2	2
3	3
4	4
5	5
6	6
7	7
8	8
9	9

43. Altogether, what was the total number of years you regularly took ESTROGENS for symptoms related to MENOPAUSE?

- ◯ <1 years
- ◯ 1- 2 years
- ◯ 3 - 4 years
- ◯ 5 - 9 years
- ◯ 10+ years

44. Are you currently taking ESTROGENS for symptoms related to MENOPAUSE?

◯ No ◯ Yes

45. Have you ever taken oral PROGESTINS (such as Provera) in combination with estrogens for symptoms or conditions associated with MENOPAUSE?

◯ No ◯ Yes

46. Did you ever take DES (diethylstilbestrol) to prevent miscarriage during any of your pregnancies?

◯ No ◯ Don't know
◯ Yes

47. Did your mother take DES while she was pregnant with you?

◯ No ◯ Don't know
◯ Yes

48. Have you ever had a BREAST BIOPSY or aspiration (needle inserted to remove fluid)?

- ◯ No (GO TO QUESTION 53)
- ◯ Yes, biopsy and aspiration
- ◯ Yes, biopsy only
- ◯ Yes, aspiration only

49. How old were you when you had your first BREAST BIOPSY or aspiration?

AGE

0	0
1	1
2	2
3	3
4	4
5	5
6	6
7	7
8	8
9	9

50. How many BREAST BIOPSIES or aspirations resulted in a diagnosis of breast cancer?

◯ None ◯ 1 or more

51. How many BREAST BIOPSIES and/or aspirations DID NOT result in a diagnosis of breast cancer?

- ◯ None
- ◯ 1
- ◯ 2
- ◯ 3 - 4
- ◯ 5 - 6
- ◯ 7+

52. Please indicate the results of all breast biopsies and/or aspirations. For each result marked, record the year the first biopsy or aspiration was done for that condition. MARK ALL THAT APPLY.

Results of Biopsies/Aspirations	Mark here for "yes"	Year of first biopsy or aspiration for condition
No abnormality detected	◯ →	19 ____
Cyst	◯ →	19 ____
Fibrocystic disease	◯ →	19 ____
Fibroadenoma	◯ →	19 ____
Hyperplasia	◯ →	19 ____
Atypical hyperplasia	◯ →	19 ____
Lobular carcinoma in situ	◯ →	19 ____
Ductal carcinoma in situ	◯ →	19 ____
Breast cancer (invasive)	◯ →	19 ____
Don't know	◯ →	19 ____
Other, specify: ________	◯ →	19 ____

F. MALE AND FEMALE REPRODUCTIVE HISTORY

(Both MALES and FEMALES should complete this section.)

53. Have you and your spouse (or partner) ever tried to become PREGNANT for more than two years without success?

◯ No (GO TO QUESTION 55)
◯ Yes → 54. What was the cause? MARK ALL THAT APPLY

- ◯ Ovulatory/hormonal problem
- ◯ Tubal obstruction or scarring
- ◯ Male infertility
- ◯ Endometriosis
- ◯ Other reason, specify: ________
- ◯ Cause not investigated
- ◯ Cause not found

55. For each time you (or your spouse or partner) became pregnant, please mark the outcome of the PREGNANCY. Male technologists, include only those pregnancies for which you were the biologic father.

◯ No pregnancies (GO TO QUESTION 58)

PREGNANCY OUTCOME

	Live birth	Stillbirth	Miscarriage	Abortion
1st Pregnancy	◯	◯	◯	◯
2nd Pregnancy	◯	◯	◯	◯
3rd Pregnancy	◯	◯	◯	◯
4th Pregnancy	◯	◯	◯	◯
5th Pregnancy	◯	◯	◯	◯
6th Pregnancy	◯	◯	◯	◯
7th Pregnancy	◯	◯	◯	◯
8th Pregnancy	◯	◯	◯	◯
9th Pregnancy	◯	◯	◯	◯
10th Pregnancy	◯	◯	◯	◯
11th Pregnancy	◯	◯	◯	◯
12th Pregnancy	◯	◯	◯	◯

◯ Currently pregnant

56. For each of your LIVE BORN CHILDREN, please record (a) the sex and year of birth, (b) whether the child ever had cancer, year of diagnosis, and type of cancer, and (c) whether the child is deceased, year of death, and cause of death. Do not include adopted, foster, step-children or those who were stillborn. Start with the oldest live born child and record in birth order for all live born children.

◯ I have not had any live born children (GO TO QUESTION 58)

FIRST CHILD (born alive)

a. Sex of child	b. Was this child ever diagnosed with cancer?	c. Is this child deceased?
◯ Male<br>◯ Female	◯ No ◯ Don't know<br>◯ Yes	◯ No (next child)<br>◯ Yes<br>◯ Don't know
Year of birth<br>19 __ __<br>⓪⓪ ①① ②② ③③ ④④ ⑤⑤ ⑥⑥ ⑦⑦ ⑧⑧ ⑨⑨	Year cancer diagnosed<br>19 __ __<br>⓪⓪ ①① ②② ③③ ④④ ⑤⑤ ⑥⑥ ⑦⑦ ⑧⑧ ⑨⑨	Year of death<br>19 __ __<br>⓪⓪ ①① ②② ③③ ④④ ⑤⑤ ⑥⑥ ⑦⑦ ⑧⑧ ⑨⑨
	Type of cancer:<br>◯ Leukemia<br>◯ Lymphoma<br>◯ Brain<br>◯ Other, specify<br>________	Cause of death:<br>________<br>________

THIRD CHILD (born alive)

a. Sex of child	b. Was this child ever diagnosed with cancer?	c. Is this child deceased?
◯ Male<br>◯ Female	◯ No ◯ Don't know<br>◯ Yes	◯ No (next child)<br>◯ Yes<br>◯ Don't know
Year of birth<br>19 __ __<br>⓪⓪ ①① ②② ③③ ④④ ⑤⑤ ⑥⑥ ⑦⑦ ⑧⑧ ⑨⑨	Year cancer diagnosed<br>19 __ __<br>⓪⓪ ①① ②② ③③ ④④ ⑤⑤ ⑥⑥ ⑦⑦ ⑧⑧ ⑨⑨	Year of death<br>19 __ __<br>⓪⓪ ①① ②② ③③ ④④ ⑤⑤ ⑥⑥ ⑦⑦ ⑧⑧ ⑨⑨
	Type of cancer:<br>◯ Leukemia<br>◯ Lymphoma<br>◯ Brain<br>◯ Other, specify<br>________	Cause of death:<br>________<br>________

SECOND CHILD (born alive)

a. Sex of child	b. Was this child ever diagnosed with cancer?	c. Is this child deceased?
◯ Male<br>◯ Female	◯ No ◯ Don't know<br>◯ Yes	◯ No (next child)<br>◯ Yes<br>◯ Don't know
Year of birth<br>19 __ __<br>⓪⓪ ①① ②② ③③ ④④ ⑤⑤ ⑥⑥ ⑦⑦ ⑧⑧ ⑨⑨	Year cancer diagnosed<br>19 __ __<br>⓪⓪ ①① ②② ③③ ④④ ⑤⑤ ⑥⑥ ⑦⑦ ⑧⑧ ⑨⑨	Year of death<br>19 __ __<br>⓪⓪ ①① ②② ③③ ④④ ⑤⑤ ⑥⑥ ⑦⑦ ⑧⑧ ⑨⑨
	Type of cancer:<br>◯ Leukemia<br>◯ Lymphoma<br>◯ Brain<br>◯ Other, specify<br>________	Cause of death:<br>________<br>________

FOURTH CHILD (born alive)

a. Sex of child	b. Was this child ever diagnosed with cancer?	c. Is this child deceased?
◯ Male<br>◯ Female	◯ No ◯ Don't know<br>◯ Yes	◯ No (next child)<br>◯ Yes<br>◯ Don't know
Year of birth<br>19 __ __<br>⓪⓪ ①① ②② ③③ ④④ ⑤⑤ ⑥⑥ ⑦⑦ ⑧⑧ ⑨⑨	Year cancer diagnosed<br>19 __ __<br>⓪⓪ ①① ②② ③③ ④④ ⑤⑤ ⑥⑥ ⑦⑦ ⑧⑧ ⑨⑨	Year of death<br>19 __ __<br>⓪⓪ ①① ②② ③③ ④④ ⑤⑤ ⑥⑥ ⑦⑦ ⑧⑧ ⑨⑨
	Type of cancer:<br>◯ Leukemia<br>◯ Lymphoma<br>◯ Brain<br>◯ Other, specify<br>________	Cause of death:<br>________<br>________

FIFTH CHILD (born alive)

a. Sex of child
◯ Male
◯ Female

Year of birth
19 ☐☐
(0 0, 1 1, 2 2, 3 3, 4 4, 5 5, 6 6, 7 7, 8 8, 9 9)

b. Was this child ever diagnosed with cancer?
◯ No ◯ Don't know
◯ Yes

Year cancer diagnosed
19 ☐☐
(0 0, 1 1, 2 2, 3 3, 4 4, 5 5, 6 6, 7 7, 8 8, 9 9)

Type of cancer:
◯ Leukemia
◯ Lymphoma
◯ Brain
◯ Other, specify ________

c. Is this child deceased?
◯ No (next child)
◯ Yes
◯ Don't know

Year of death
19 ☐☐
(0 0, 1 1, 2 2, 3 3, 4 4, 5 5, 6 6, 7 7, 8 8, 9 9)

Cause of death:
____
____

SIXTH CHILD (born alive)

a. Sex of child
◯ Male
◯ Female

Year of birth
19 ☐☐
(0 0, 1 1, 2 2, 3 3, 4 4, 5 5, 6 6, 7 7, 8 8, 9 9)

b. Was this child ever diagnosed with cancer?
◯ No ◯ Don't know
◯ Yes

Year cancer diagnosed
19 ☐☐
(0 0, 1 1, 2 2, 3 3, 4 4, 5 5, 6 6, 7 7, 8 8, 9 9)

Type of cancer:
◯ Leukemia
◯ Lymphoma
◯ Brain
◯ Other, specify ________

c. Is this child deceased?
◯ No (next child)
◯ Yes
◯ Don't know

Year of death
19 ☐☐
(0 0, 1 1, 2 2, 3 3, 4 4, 5 5, 6 6, 7 7, 8 8, 9 9)

Cause of death:
____
____

SEVENTH CHILD (born alive)

a. Sex of child
◯ Male
◯ Female

Year of birth
19 ☐☐
(0 0, 1 1, 2 2, 3 3, 4 4, 5 5, 6 6, 7 7, 8 8, 9 9)

b. Was this child ever diagnosed with cancer?
◯ No ◯ Don't know
◯ Yes

Year cancer diagnosed
19 ☐☐
(0 0, 1 1, 2 2, 3 3, 4 4, 5 5, 6 6, 7 7, 8 8, 9 9)

Type of cancer:
◯ Leukemia
◯ Lymphoma
◯ Brain
◯ Other, specify ________

c. Is this child deceased?
◯ No (next child)
◯ Yes
◯ Don't know

Year of death
19 ☐☐
(0 0, 1 1, 2 2, 3 3, 4 4, 5 5, 6 6, 7 7, 8 8, 9 9)

Cause of death:
____
____

EIGHTH CHILD (born alive)

a. Sex of child
◯ Male
◯ Female

Year of birth
19 ☐☐
(0 0, 1 1, 2 2, 3 3, 4 4, 5 5, 6 6, 7 7, 8 8, 9 9)

b. Was this child ever diagnosed with cancer?
◯ No ◯ Don't know
◯ Yes

Year cancer diagnosed
19 ☐☐
(0 0, 1 1, 2 2, 3 3, 4 4, 5 5, 6 6, 7 7, 8 8, 9 9)

Type of cancer:
◯ Leukemia
◯ Lymphoma
◯ Brain
◯ Other, specify ________

c. Is this child deceased?
◯ No (next child)
◯ Yes
◯ Don't know

Year of death
19 ☐☐
(0 0, 1 1, 2 2, 3 3, 4 4, 5 5, 6 6, 7 7, 8 8, 9 9)

Cause of death:
____
____

If you have had more than eight live born children, please record answers to Question 56 for the additional children on a separate sheet of paper and return it with your completed questionnaire.

57. If any of your live born children had a BIRTH DEFECT, please record the type of defect in the appropriate column for that child. Mark all that apply for each child. Leave the columns blank for those children who had no birth defects.

◯ None of my children have had a birth defect (GO TO QUESTION 58)

BIRTH DEFECT	BIRTH ORDER OF LIVE BORN CHILDREN							
	1st	2nd	3rd	4th	5th	6th	7th	8th
Cataracts	◯	◯	◯	◯	◯	◯	◯	◯
Cleft lip or palate	◯	◯	◯	◯	◯	◯	◯	◯
Club foot	◯	◯	◯	◯	◯	◯	◯	◯
Down's Syndrome	◯	◯	◯	◯	◯	◯	◯	◯
Extra fingers, shortened limbs, or any other skeletal abnormality	◯	◯	◯	◯	◯	◯	◯	◯
Hole in the heart or other congenital heart defect	◯	◯	◯	◯	◯	◯	◯	◯
Hydrocephalus (excess water around or within the brain)	◯	◯	◯	◯	◯	◯	◯	◯
Small head size (microcephaly)	◯	◯	◯	◯	◯	◯	◯	◯
Spina bifida or other neural tube defect	◯	◯	◯	◯	◯	◯	◯	◯
Undescended testicle	◯	◯	◯	◯	◯	◯	◯	◯
Other birth defects Specify type of defect(s) →	◯	◯	◯	◯	◯	◯	◯	◯

If you have had more than eight live born children with a birth defect, please record answers to Question 57 for the additional children on a separate sheet of paper and return it with your completed questionnaire.

G. FAMILY HISTORY

58. Please indicate whether any of the following blood related FAMILY MEMBERS have had CANCER. If yes, please specify the primary site where the first cancer started and the age at diagnosis. Do not list metastases, or basal or squamous cell skin cancer.

FAMILY MEMBER	No	Don't Know	Yes	PRIMARY CANCER SITE	AGE AT DIAGNOSIS
Father	◯	◯	◯→	________	____
Mother	◯	◯	◯→	________	____
Maternal Grandfather	◯	◯	◯→	________	____
Maternal Grandmother	◯	◯	◯→	________	____
Paternal Grandfather	◯	◯	◯→	________	____
Paternal Grandmother	◯	◯	◯→	________	____

59. Have any of your blood related BROTHERS OR SISTERS had CANCER?

◯ No DON'T KNOW (GO TO QUESTION 60)

◯ Yes → If yes, please specify the primary site where the first cancer started. Do not list metastases, or basal or squamous cell skin cancer.

SIBLING				PRIMARY CANCER SITE	AGE AT DIAGNOSIS
Sister	◯	Brother	◯	________	____
Sister	◯	Brother	◯	________	____
Sister	◯	Brother	◯	________	____

60. Please indicate below if any of your female blood relatives, including those previously reported, have had BREAST CANCER? Mark all that apply.

◯ None
◯ Mother
◯ Sister
◯ Daughter
◯ Grandmother, maternal
◯ Grandmother, paternal
◯ Aunt, maternal
◯ Aunt, paternal
◯ Don't know

61. Are you a TWIN?

◯ No
◯ Don't know
◯ Yes, identical
◯ Yes, fraternal, same sex
◯ Yes, fraternal, opposite sex
◯ Yes, type unknown, same sex

62. How many blood related SISTERS and BROTHERS, living and dead, do you have? If none or don't know, record "00."

NO. OF SISTERS		NO. OF BROTHERS	
⓪	⓪	⓪	⓪
①	①	①	①
②	②	②	②
	③		③
	④		④
	⑤		⑤
	⑥		⑥
	⑦		⑦
	⑧		⑧
	⑨		⑨

H. VITAMIN AND MEDICATION USE

63. Since the age of 25, have you at any time taken any MULTIVITAMINS or other VITAMIN OR MINERAL SUPPLEMENTS on a regular basis, that is, at least once a week for 6 months or more?
    - ○ No (FEMALES GO TO QUESTION 65, PAGE 14; MALES GO TO QUESTION 66, PAGE 14)
    - ○ Yes

For each dietary supplement listed below that you have taken regularly since the age of 25, specify 1) how many years, 2) during which time periods, and 3) the number of pills or dose, usually taken. Even if you're not sure about a response, we'd prefer a good guess to a "don't know." For each supplement that you have not taken regularly, mark the circle for "Never or not taken regularly" and leave all other columns blank for that supplement.

MULTIVITAMINS	NEVER OR NOT TAKEN REGULARLY	HOW MANY YEARS TAKEN?							TIME PERIODS TAKEN (Mark all that apply)				HOW MANY PILLS HAVE YOU USUALLY TAKEN?				
		1	2-4	5-9	10-14	15-19	20-24	25+	<1980	1980-1984	1985-1989	1990+	<2 week	2-4/ week	5-6/ week	1/ day	2+/ day
One-a-Day type (100% RDA)	○	○	○	○	○	○	○	○	○	○	○	○	○	○	○	○	○
High Potency type (More than 100% RDA such as Theragran)	○	○	○	○	○	○	○	○	○	○	○	○	○	○	○	○	○
B-Complex	○	○	○	○	○	○	○	○	○	○	○	○	○	○	○	○	○
Stresstabs (B-Complex + Vitamin C)	○	○	○	○	○	○	○	○	○	○	○	○	○	○	○	○	○
Other Multivitamins	○	○	○	○	○	○	○	○	○	○	○	○	○	○	○	○	○

For any multivitamin you are now taking, please copy the exact type and brand from the label:

○ Not currently taking a multivitamin

INDIVIDUAL SUPPLEMENTS	NEVER OR NOT TAKEN REGULARLY	HOW MANY YEARS TAKEN?							TIME PERIODS TAKEN (Mark all that apply)				WHAT DOSE PER DAY HAVE YOU USUALLY TAKEN? (Indicate teaspoons or capsules)				
		1	2-4	5-9	10-14	15-19	20-24	25+	<1980	1980-1984	1985-1989	1990+	<1	1	2	>2	
Cod Liver Oil or Fish Liver Oil (I.U.)	○	○	○	○	○	○	○	○	○	○	○	○	○	○	○	○	○ teaspoons<br>○ capsules

	NEVER OR NOT TAKEN REGULARLY	HOW MANY YEARS TAKEN?							TIME PERIODS TAKEN (Mark all that apply)				WHAT DOSE PER DAY HAVE YOU USUALLY TAKEN? (Select the closest amount)					
		1	2-4	5-9	10-14	15-19	20-24	25+	<1980	1980-1984	1985-1989	1990+						
													5000	10000	15000	20000	25000+	Don't Know
Vitamin A (I.U.)	○	○	○	○	○	○	○	○	○	○	○	○	○	○	○	○	○	○
													5000	10000	15000	20000	25000+	Don't Know
Beta-Carotene (I.U.)	○	○	○	○	○	○	○	○	○	○	○	○	○	○	○	○	○	○
													100	250	500	1000	1500+	Don't Know
Vitamin C (mg)	○	○	○	○	○	○	○	○	○	○	○	○	○	○	○	○	○	○
													100	200	400	600	1000+	Don't Know
Vitamin E (I.U.)	○	○	○	○	○	○	○	○	○	○	○	○	○	○	○	○	○	○
													100	250	500	800	1200+	Don't Know
Calcium, Dolomite, Tums, etc. (mg. of elemental calcium)	○	○	○	○	○	○	○	○	○	○	○	○	○	○	○	○	○	○
Vitamin D (alone or with calcium or Vit. A)	○	○	○	○	○	○	○	○	○	○	○	○						

64. Which of the following INDIVIDUAL SUPPLEMENTS are you currently taking on a regular basis? Multivitamins contain many of these vitamins or minerals, but here we are interested in supplements that are taken in addition to a multivitamin. MARK ALL THAT APPLY.
- ◯ None
- ◯ Brewer's yeast
- ◯ Thiamine
- ◯ Riboflavin
- ◯ Niacin
- ◯ Vitamin B-6
- ◯ Folic acid or folate
- ◯ Iron
- ◯ Magnesium
- ◯ Selenium
- ◯ Zinc
- ◯ Omega fatty acids

MALES GO TO QUESTION 66)

65. During how many pregnancies did you take prescription prenatal vitamins for at least 3 months?
- ◯ None or never pregnant
- ◯ 1 pregnancy
- ◯ 2 pregnancies
- ◯ 3 pregnancies
- ◯ 4 pregnancies
- ◯ 5 OR MORE pregnancies

66. During the past year, on average, how many days each month did you take the following MEDICATIONS? Please mark one column for each medication.

AVERAGE DAYS PER MONTH

MEDICATION	None	<1	1 - 4	5 - 14	15 - 21	22+
Acetaminophen (e.g., Tylenol)	◯	◯	◯	◯	◯	◯
Aspirin (e.g., Anacin, Bufferin, Midol, Alka-Seltzer)	◯	◯	◯	◯	◯	◯
Other anti-inflammatory (e.g., Ibuprofen, Motrin, Naprosyn, Advil)	◯	◯	◯	◯	◯	◯
Thyroid hormones (e.g., Synthroid)	◯	◯	◯	◯	◯	◯
Tranquilizers (e.g., Valium)	◯	◯	◯	◯	◯	◯

67. Have you ever taken prescription DIURETICS on a regular basis?
- ◯ No (GO TO QUESTION 69)
- ◯ Yes

68. On average, how long have you taken prescription DIURETICS on a regular basis?
- ◯ <6 months
- ◯ 6 - 11 months
- ◯ 1 - 2 years
- ◯ More than 2 years

I. OTHER FACTORS

69. What is the color of your EYES?
- ◯ Blue
- ◯ Brown
- ◯ Green
- ◯ Hazel
- ◯ Grey
- ◯ Black
- ◯ Other, specify below ____________

70. What do you consider your SKIN complexion to be?
- ◯ Fair
- ◯ Medium
- ◯ Dark

71. What was your natural HAIR COLOR when you were 15 years old?
- ◯ Blonde
- ◯ Light brown
- ◯ Dark brown/brunette
- ◯ Red or Auburn
- ◯ Black
- ◯ Other, specify below ____________

72. Have you ever used permanent or semi-permanent HAIR DYE regularly? By regular use we mean at least twice a year for 2 consecutive years. Please do not include temporary rinses or bleach/highlights.
- ◯ No (GO TO QUESTION 76, PAGE 15)
- ◯ Yes

73. About how many times per year did you use permanent or semi-permanent HAIR DYES?

TIMES PER YEAR
[ ][ ]
First digit: ⓪ ① ②
Second digit: ⓪ ① ② ③ ④ ⑤ ⑥ ⑦ ⑧ ⑨

74. For how many years have you used permanent or semi-permanent HAIR DYES regularly?

YEARS
[ ][ ]
First digit: ⓪ ① ② ③ ④ ⑤
Second digit: ⓪ ① ② ③ ④ ⑤ ⑥ ⑦ ⑧ ⑨

75. What color permanent or semi-permanent HAIR DYES did you use the most?
- ◯ Blonde
- ◯ Brown/brunette
- ◯ Black
- ◯ Red or red/brown
- ◯ Other, specify ____________

76. During the past year, how often did you drink the following BEVERAGES?

BEVERAGE (AVERAGE SERVING)	AVERAGE NUMBER OF SERVINGS CONSUMED DURING PAST YEAR												
	Never	<1/ month	1/ month	2 - 3/ month	1/ week	2/ week	3 - 4/ week	5 - 6/ week	1/ day	2/ day	3/ day	4/ day	5+/ day
100% fruit juice (including fortified) (4 - 6 oz.)	○	○	○	○	○	○	○	○	○	○	○	○	○
Fruit drinks fortified with Vitamin C (4 - 6 oz.)	○	○	○	○	○	○	○	○	○	○	○	○	○
Orange juice fortified with calcium (4 - 6 oz.)	○	○	○	○	○	○	○	○	○	○	○	○	○
Whole milk (4%) (8 oz.)	○	○	○	○	○	○	○	○	○	○	○	○	○
Lowfat milk (1 - 2%) (8 oz.)	○	○	○	○	○	○	○	○	○	○	○	○	○
Skim or nonfat milk (0 - 1/2%) (8 oz.)	○	○	○	○	○	○	○	○	○	○	○	○	○
Meal-replacement beverages, such as Instant Breakfast	○	○	○	○	○	○	○	○	○	○	○	○	○
Low-calorie meal-replacement beverages, such as Slim Fast	○	○	○	○	○	○	○	○	○	○	○	○	○
Cola, regular (12 oz.)	○	○	○	○	○	○	○	○	○	○	○	○	○
Cola, decaffeinated (12 oz.)	○	○	○	○	○	○	○	○	○	○	○	○	○
Coffee, regular (6 oz.)	○	○	○	○	○	○	○	○	○	○	○	○	○
Coffee, decaffeinated (6 oz.)	○	○	○	○	○	○	○	○	○	○	○	○	○
Tea (6 oz.)	○	○	○	○	○	○	○	○	○	○	○	○	○
Beer (12 oz.)	○	○	○	○	○	○	○	○	○	○	○	○	○
Wine (4 oz.)	○	○	○	○	○	○	○	○	○	○	○	○	○
Liquor (1 shot)	○	○	○	○	○	○	○	○	○	○	○	○	○
Water (8 oz.)	○	○	○	○	○	○	○	○	○	○	○	○	○

77. During the past year, how often did you eat the following FOODS?

TYPE OF FOOD	AVERAGE NUMBER OF SERVINGS CONSUMED DURING PAST YEAR												
	Never	<1/ month	1/ month	2 - 3/ month	1/ week	2/ week	3 - 4/ week	5 - 6/ week	1/ day	2/ day	3/ day	4/ day	5+/ day
Fresh fruit	○	○	○	○	○	○	○	○	○	○	○	○	○
Canned fruit	○	○	○	○	○	○	○	○	○	○	○	○	○
Baked, boiled or mashed potatoes (excluding french fries)	○	○	○	○	○	○	○	○	○	○	○	○	○
Cooked vegetables (excluding potatoes, rice or beans)	○	○	○	○	○	○	○	○	○	○	○	○	○
Beans, such as kidney, pinto, baked or refried	○	○	○	○	○	○	○	○	○	○	○	○	○
Lettuce salad	○	○	○	○	○	○	○	○	○	○	○	○	○
Raw vegetables (excluding lettuce)	○	○	○	○	○	○	○	○	○	○	○	○	○
Beef	○	○	○	○	○	○	○	○	○	○	○	○	○
Pork	○	○	○	○	○	○	○	○	○	○	○	○	○
Chicken or turkey	○	○	○	○	○	○	○	○	○	○	○	○	○
Fish (excluding canned fish, such as tuna)	○	○	○	○	○	○	○	○	○	○	○	○	○
100% fortified cold cereal, such as Product 19, Total or Just Right	○	○	○	○	○	○	○	○	○	○	○	○	○
High-fiber cereal, such as All-Bran, Grapenuts, Wheaties, Granola	○	○	○	○	○	○	○	○	○	○	○	○	○
Any other cold cereal	○	○	○	○	○	○	○	○	○	○	○	○	○
Whole grain breads or rolls, such as rye, pumpernickel, whole wheat	○	○	○	○	○	○	○	○	○	○	○	○	○
White bread or rolls	○	○	○	○	○	○	○	○	○	○	○	○	○
Cottage cheese or yogurt	○	○	○	○	○	○	○	○	○	○	○	○	○
Hard or soft cheese (excluding cottage cheese)	○	○	○	○	○	○	○	○	○	○	○	○	○
Fried chicken, fried fish or fried potatoes	○	○	○	○	○	○	○	○	○	○	○	○	○
Cookies, cake or pie	○	○	○	○	○	○	○	○	○	○	○	○	○

78. During the past year, on average how many hours per week did you spend EXERCISING OR WALKING?

HOURS PER WEEK	NONE	<1 hr.	1-3 hrs.	4-9 hrs.	10-19 hrs.	20-39 hrs.	Over 40 hrs.
Exercising strenuously (e.g., aerobics, jogging, swimming)	◯	◯	◯	◯	◯	◯	◯
Walking or hiking for exercise	◯	◯	◯	◯	◯	◯	◯
Walking at home or at work	◯	◯	◯	◯	◯	◯	◯

79. During the past year, on average how many FLIGHTS OF STAIRS (not individual steps) did you climb daily?
- ◯ None
- ◯ 1 - 2
- ◯ 3 - 4
- ◯ 5 - 9
- ◯ 10 - 14
- ◯ 15 or more

80. How would you rate your overall health?
- ◯ Excellent
- ◯ Good
- ◯ Fair
- ◯ Poor

81. Are you of Celtic or Gaelic ancestry?
- ◯ No
- ◯ Yes
- ◯ Don't know

82. Sometime in the future, would you be willing to donate a small venous blood sample if we sent you a convenient collection kit? This would involve having someone draw your blood, but would not require any centrifugation or other processing. This would allow researchers to investigate the long-term effects of low-level fractionated radiation exposures.
- ◯ No, I would not be willing to donate blood
- ◯ Yes, I would be willing to donate blood

83. What is your SOCIAL SECURITY NUMBER?

SOCIAL SECURITY NUMBER

			–			–				
0	0	0		0	0		0	0	0	0
1	1	1		1	1		1	1	1	1
2	2	2		2	2		2	2	2	2
3	3	3		3	3		3	3	3	3
4	4	4		4	4		4	4	4	4
5	5	5		5	5		5	5	5	5
6	6	6		6	6		6	6	6	6
7	7	7		7	7		7	7	7	7
8	8	8		8	8		8	8	8	8
9	9	9		9	9		9	9	9	9

Your social security number is being requested under Section 411, Public Health Service Act [42 USC 285a]. The primary use of this information is for researchers to locate you in the future and to search vital records in a follow-up study conducted. Additional disclosures of information may be: to the Department of Health and Human Services contractors, grantees and collaborating researchers and their staff in order to accomplish the research purpose for which the records are collected; to a congressional office in response to a request made by you; and as otherwise required by Law. Furnishing your Social Security Number is voluntary, and you will not be denied any Federal right, benefit, or privilege by your refusal to disclose it.

THANK YOU VERY MUCH FOR YOUR HELP.

Please take a minute or two to recheck your questionnaire to be sure you have not missed any questions or skipped any items on the lists or tables.

Please use this space for any additional comments or information.

FOR OFFICE USE ONLY

A	K
B	L
C	M
D	N
E	O
F	P
G	Q
H	R
I	S
J	T

R9290-Data Recognition Corp.-54321

OMB 0925-0405
Expires: 2/28/2007

USRT

U.S. Radiologic Technologists Study

THIRD SURVEY

A collaborative effort between the University of Minnesota School of Public Health, National Cancer Institute, and American Registry of Radiologic Technologists.

CONFIDENTIALITY:
Please be assured that all information you provide will be kept confidential and will not be disclosed to anyone but the researchers conducting this study, except as otherwise required by law. Any published results from this survey will be reported in statistical summaries only and will never include a participant's name. Your participation in this study is completely voluntary and failure to answer any particular question or the information collection as a whole will not affect your future contacts with the University of Minnesota, the American Registry of Radiologic Technologists, or the National Institutes of Health.

OFFICE USE ONLY

☐ A	☐ B	☐ C	☐ D	☐ E
☐ F	☐ G	☐ H	☐ I	☐ J

INSTRUCTIONS:
You may use a blue or black pen when completing this form. Please return completed questionnaire in the postage-paid envelope provided.

Please answer each question to the best of your recollection. Even if you are unable to recall answers exactly, your best estimates will help us better understand exposure levels during different time periods.

MARKING INSTRUCTIONS

RIGHT ☒ **WRONG** ✓ ⊠ ■

Please use blue or black pen.

PARTICIPANT INFORMATION

1. What is today's date?

M M – D D – Y Y Y Y

2. Are you male or female?

☐ Male

☐ Female

3. What is your date of birth?

M M – D D – Y Y Y Y

MEDICAL HISTORY

The first part of this questionnaire includes questions to update the health information you provided in the last survey.

4. Did a doctor ever tell you that you had any of the following types of CANCER or malignant tumors?
For each type of cancer that you mark YES, please provide the year it was first diagnosed.

Type of Cancer	Yes	Year 1st Diagnosed
Bladder	☐ →	Y Y Y Y
Bone	☐ →	Y Y Y Y
Brain or nervous system	☐ →	Y Y Y Y
Breast	☐	

If YES:

Which Breast? Left	Right	What type was it? Invasive Cancer	Ductal Carcinoma *In Situ*	Other Or Type Unknown	Year 1st Diagnosed
☐	☐	☐	☐	☐	Y Y Y Y
☐	☐	☐	☐	☐	Y Y Y Y

Type of Cancer	Yes	Year 1st Diagnosed
Cervix (invasive, not *in situ*)	☐ →	Y Y Y Y
Colon	☐ →	Y Y Y Y
Connective or other soft tissue	☐ →	Y Y Y Y
Esophagus	☐ →	Y Y Y Y
Hodgkin's Disease	☐ →	Y Y Y Y
Kidney	☐ →	Y Y Y Y
Larynx	☐ →	Y Y Y Y
Leukemia, Acute Lymphocytic	☐ →	Y Y Y Y
Leukemia, Chronic Lymphocytic	☐ →	Y Y Y Y
Leukemia, Acute Myeloid	☐ →	Y Y Y Y
Leukemia, Chronic Myeloid	☐ →	Y Y Y Y
Leukemia, other or type unknown	☐ →	Y Y Y Y
Liver	☐ →	Y Y Y Y
Lung, trachea or bronchus	☐ →	Y Y Y Y
Lymphoma, Non-Hodgkin's	☐ →	Y Y Y Y
Lymphoma, other or type unknown	☐ →	Y Y Y Y
Melanoma of the Skin	☐ →	Y Y Y Y
Melanoma of the Eye	☐ →	Y Y Y Y

Type of Cancer	Yes	Year 1st Diagnosed
Melanoma, Other	☐	Y Y Y Y
Multiple myeloma	☐	Y Y Y Y
Ovary	☐	Y Y Y Y
Pancreas	☐	Y Y Y Y
Pharynx	☐	Y Y Y Y
Prostate	☐	Y Y Y Y
Rectum	☐	Y Y Y Y
Salivary gland	☐	Y Y Y Y
Skin cancer other than melanoma	☐	

If YES:

What type?	Yes	Year 1st Diagnosed
Basal cell	☐	Y Y Y Y
Squamous cell	☐	Y Y Y Y
Other or type unknown	☐	Y Y Y Y

	Yes	
Stomach	☐	Y Y Y Y
Testis	☐	Y Y Y Y
Thyroid	☐	Y Y Y Y
Uterus (endometrium)	☐	Y Y Y Y
Any other type of cancer (including unknown types)	☐	

If YES:

Type: ______________________ → Y Y Y Y

Type: ______________________ → Y Y Y Y

4a. Mark here if you were <u>never diagnosed</u> with any cancer ☐

5. Did a doctor ever tell you that you had any of the following <u>BENIGN tumors</u> or <u>other medical conditions</u>? *For each condition you mark YES, please provide the year it was first diagnosed.*

Medical Condition	Yes	Year 1st Diagnosed
<u>Benign</u> tumor of the brain or nervous system	☐	Y Y Y Y

If YES: what type of tumor?

☐ Meningioma?
☐ Schwannoma?
☐ Acoustic neuroma?
☐ Other or type unknown. Specify: ______________________

Thyroid conditions:

	Yes	
Benign thyroid tumor (adenoma)	☐	Y Y Y Y
Thyroid nodule	☐	Y Y Y Y
Goiter (enlarged thyroid)	☐	Y Y Y Y
Thyroiditis (Hashimoto's Disease)	☐	Y Y Y Y
Hyp<u>er</u>thyroidism (overactive thyroid)	☐	Y Y Y Y
Hyp<u>o</u>thyroidism (underactive thyroid)	☐	Y Y Y Y
Any other thyroid conditions	☐	Y Y Y Y
Pituitary tumor	☐	Y Y Y Y
Hyperparathyroidism	☐	Y Y Y Y

[WOMEN ONLY]	Yes	Year 1st Diagnosed
Uterine fibroids (myoma)?	☐	Y Y Y Y
Fibrocystic or other benign breast disease such as fibroadenoma or hyperplasia?	☐	

If YES, was it confirmed by breast biopsy or aspiration? No ☐ Yes ☐ → Y Y Y Y

5a. Mark here if you were <u>never diagnosed</u> with any of the above benign tumors or medical conditions ☐

6. **Did a doctor ever tell you that you had any of the following cardiovascular conditions?** *For each condition you mark YES, please provide the year it was first diagnosed.*

Medical Condition	Yes	Year 1st Diagnosed
**High blood pressure**	☐	YYYY
**Stroke (cerebrovascular accident)**	☐	YYYY
**TIA (transient ischemic attack)**	☐	YYYY
**Heart attack, myocardial infarction (MI)**	☐	YYYY
**Angina pectoris**	☐	YYYY

If YES (high blood pressure), have you ever taken medicine for high blood pressure? No ☐ Yes ☐

If YES (angina pectoris), was this confirmed by angiography? No ☐ Yes ☐

6a. **Mark here if you were never diagnosed with any of the above cardiovascular conditions** ☐

7. **Did a doctor ever tell you that you had any of the following eye conditions?** *For each condition you mark YES, please provide the year it was first diagnosed.*

Medical Condition	Yes	Year 1st Diagnosed
**Macular degeneration**	☐	YYYY
**Cataracts**	☐	YYYY
**Glaucoma**	☐	YYYY

If YES (cataracts), did you have any cataracts removed? No ☐ Yes ☐

Year 1st Removed: YYYY

7a. **Mark here if you were never diagnosed with any of the above eye conditions** ☐

8. **Did a doctor ever tell you that you had any of the following medical conditions?** *For each condition you mark YES, please provide the year it was first diagnosed.*

Medical Condition	Yes	Year 1st Diagnosed
**Cirrhosis of the liver**	☐	YYYY
**Diabetes**	☐	YYYY
**Osteoporosis**	☐	YYYY
**Multiple sclerosis**	☐	YYYY
**Parkinson's Disease**	☐	YYYY
**Attention-deficit disorder (with or without hyperactivity)**	☐	YYYY
**Systemic Lupus Erythematosus**	☐	YYYY
**Osteoarthritis**	☐	YYYY
**Rheumatoid arthritis**	☐	YYYY
**Scleroderma**	☐	YYYY

If YES (diabetes), do you currently take insulin for diabetes? No ☐ Yes ☐

If YES (attention-deficit disorder), at what age, did you first take Ritalin or other stimulant medication for this condition? Age ☐☐ Never Took ☐

8a. **Mark here if you were never diagnosed with any of the above other conditions** ☐

9. **(Without shoes or clothes), about how much did you weigh when you were . . .**

18-22 years of age	☐☐☐	POUNDS
in your 30s	☐☐☐	POUNDS
in your 40s	☐☐☐	POUNDS
in your 50s	☐☐☐	POUNDS
currently	☐☐☐	POUNDS

10. If you gained weight when you were between 40 and 59 years old, where on your body did you mainly tend to add the weight at this time? (SELECT THE ONE BEST ANSWER.)

- ☐ Did not gain weight between 40 and 59 years old.
- ☐ Around the chest or shoulders
- ☐ Around the waist or stomach
- ☐ Around the hips or thighs
- ☐ Equally all over

SMOKING HISTORY

11. Do you smoke cigarettes currently?

- ☐ Yes → **[GO TO 12]**
- ☐ No

11a. If you smoked in the past, how old were you when you stopped smoking? . . . ☐☐ YEARS

12. How many cigarettes per day do you or did you usually smoke? (SELECT THE ONE BEST ANSWER)

- ☐ 1-10
- ☐ 11-20
- ☐ 21-30
- ☐ 31-40
- ☐ 41-60
- ☐ 61 or more

PHYSICAL ACTIVITY

The following questions are about recreational or leisure time physical activities during four periods of your life. When answering these questions, do not include physical activity or exercise that is part of a job.

13. On average, about how many hours per week did you participate in strenuous physical activities during each of the four ages listed below? (Leave blank if age group is not applicable to you.)

Strenuous activity means something that increases your heart rate and/or causes you to perspire *(for example, running, jogging, cross-country skiing, vigorous swimming or bicycling).*

Between ages 18-22 . . . . ☐☐ HRs/WK   In your 50s . . . . ☐☐ HRs/WK

In your 30s . . . . ☐☐ HRs/WK   Currently . . . . ☐☐ HRs/WK

14. On average, about how many hours per week did you participate in moderate physical activities during each of the four ages listed below? (Leave blank if age group is not applicable to you.)

Moderate activity means something that requires effort but is not exhausting *(for example, walking for exercise, easy swimming, bicycling or golfing).*

Between ages 18-22 . . . . ☐☐ HRs/WK   In your 50s . . . . ☐☐ HRs/WK

In your 30s . . . . ☐☐ HRs/WK   Currently . . . . ☐☐ HRs/WK

The next section is about your work experience as a radiologic technologist.

For purposes of this survey, the term "RADIOLOGIC TECHNOLOGIST" will refer to any job in which you performed or assisted with diagnostic or therapeutic radiation procedures in a health care setting. This information will improve our ability to properly estimate radiation dose over time. Because practices and standards in the field changed over time, we are asking about four time periods.

	TIME PERIODS			
	**1950 to 1959**	**1960 to 1969**	**1970 to 1979**	**1980 to the present**
**15. How many years did you work as a radiologic technologist during each time period?**	☐☐ YEARS<br>If ZERO, skip questions 16 to 19 for this time period.	☐☐ YEARS<br>If ZERO, skip questions 16 to 19 for this time period.	☐☐ YEARS<br>If ZERO, skip questions 16 to 19 for this time period.	☐☐ YEARS<br>If ZERO, skip questions 16 to 19 for this time period.

Questions 16-19 are about the job you held the longest in each time period when you worked as a radiologic technologist.

	TIME PERIODS			
	**1950 to 1959**	**1960 to 1969**	**1970 to 1979**	**1980 to the present**
**16. For your longest job in each time period, which of the following best describes the kind of facility you worked in?**	☐ Hospital<br>☐ Physician(s) office or clinic<br>☐ Dentist's office<br>☐ Other facility	☐ Hospital<br>☐ Physician(s) office or clinic<br>☐ Dentist's office<br>☐ Other facility	☐ Hospital<br>☐ Physician(s) office or clinic<br>☐ Dentist's office<br>☐ Other facility	☐ Hospital<br>☐ Physician(s) office or clinic<br>☐ Dentist's office<br>☐ Other facility
**17. About how many hours per week did you usually work as a radiologic technologist at this longest job during each time period?**	☐☐ HOURS PER WEEK	☐☐ HOURS PER WEEK	☐☐ HOURS PER WEEK	☐☐ HOURS PER WEEK
**18. Did you ever wear a dosimetry badge while working at this longest job in each time period?**	☐ No<br>☐ Yes<br>No → Go to question 16 for next time period.	☐ No<br>☐ Yes<br>No → Go to question 16 for next time period.	☐ No<br>☐ Yes<br>No → Go to question 16 for next time period.	☐ No<br>☐ Yes<br>No → Go to question 20, page 8.
**19. When you wore an apron at this longest job in each time period, did you usually wear the dosimetry badge on the inside or outside of the apron?**	☐ inside<br>☐ outside<br>☐ never wore apron	☐ inside<br>☐ outside<br>☐ never wore apron	☐ inside<br>☐ outside<br>☐ never wore apron	☐ inside<br>☐ outside<br>☐ never wore apron

20. Did you work as a radiologic technologist between 1950 through 1959?
- ☐ Yes
- ☐ No → **[GO TO 44 ON PAGE 10]**

The following questions are about *some types* of radiologic procedures you may have worked or assisted with <u>on the job you held the longest between 1950 and 1959</u>. You don't need to know exact answers. Just choose the answers that best fit your experience.

ROUTINE DIAGNOSTIC X-RAY
(such as chest x-rays, portable x-rays, mammograms, etc.)

21. How many ROUTINE DIAGNOSTIC X-RAY procedures did you perform or assist with <u>during a typical week at this longest job in the 1950s</u>?
- ☐ zero **[GO TO 27]**
- ☐ less than 10 per week
- ☐ 10-24 per week
- ☐ 25-49 per week
- ☐ 50-99 per week
- ☐ 100 or more per week

22. When performing ROUTINE DIAGNOSTIC X-RAY procedures, what percentage of the time were you <u>inside</u> the room?
[*Inside* the room includes standing behind a shield or partial wall open to the patient. *Outside* means separated from the patient by the room walls and a door.]
- ☐ zero **[GO TO 27]**
- ☐ less than 25%
- ☐ 25-74%
- ☐ 75% or more

23. When performing ROUTINE DIAGNOSTIC X-RAY procedures, what percentage of the time did you wear a protective apron?
- ☐ zero
- ☐ less than 25%
- ☐ 25-74%
- ☐ 75% or more

24. When performing ROUTINE DIAGNOSTIC X-RAY procedures, what percentage of the time did you stand behind a screen, shield or other protective enclosure?
- ☐ zero **[GO TO 26]**
- ☐ less than 25%
- ☐ 25-74%
- ☐ 75% or more

25. How high did the screen, shield or other protective enclosure extend?
- ☐ above the head
- ☐ up to the shoulders
- ☐ up to the waist

26. When performing ROUTINE DIAGNOSTIC X-RAY procedures, what percentage of patients being x-rayed did you hold during the procedure?
- ☐ zero
- ☐ less than 10%
- ☐ 10-24%
- ☐ 25% or more

FLUOROSCOPY
(such as angiograms, barium swallows, etc.)

27. How many FLUOROSCOPY procedures did you perform or assist with <u>during a typical week at this longest job in the 1950s</u>?
- ☐ zero **[GO TO 33]**
- ☐ less than 10 per week
- ☐ 10-24 per week
- ☐ 25-49 per week
- ☐ 50-99 per week
- ☐ 100 or more per week

28. When performing FLUOROSCOPY procedures, what percentage of the time were you <u>inside</u> the room?
- ☐ zero **[GO TO 32]**
- ☐ less than 25%
- ☐ 25-74%
- ☐ 75% or more

29. When performing FLUOROSCOPY procedures, what percentage of the time did you wear a protective apron?
- ☐ zero
- ☐ less than 25%
- ☐ 25-74%
- ☐ 75% or more

30. When performing FLUOROSCOPY procedures, what percentage of the time did you stand behind a screen, shield or other protective enclosure?
- ☐ zero **[GO TO 32]**
- ☐ less than 25%
- ☐ 25-74%
- ☐ 75% or more

31. How high did the screen, shield or other protective enclosure extend?
- ☐ above the head
- ☐ up to the shoulders
- ☐ up to the waist

32. Excluding set-up time, what percentage of the FLUOROSCOPY procedures that you performed or assisted with were more than 15 minutes long?
- ☐ zero
- ☐ less than 25%
- ☐ 25-49%
- ☐ 50% or more

DIAGNOSTIC RADIOISOTOPE
(such as using I-131 or technecium-99M, etc.)

33. How many DIAGNOSTIC RADIOISOTOPE procedures (injections or oral procedures) did you perform or assist with <u>during a typical week at this longest job in the 1950s</u>?

- ☐ zero **[GO TO 38]**
- ☐ less than 10 per week
- ☐ 10-24 per week
- ☐ 25-49 per week
- ☐ 50-99 per week
- ☐ 100 or more per week

34. When working with DIAGNOSTIC RADIOISOTOPES, how many times did you prepare the radiopharmaceutical kit and/or elute generators *(i.e., separate and measure a concentrated amount ofisotope from its source for use in a radiopharmaceutical kit)* **<u>during a typical week at this longest job</u>?**

- ☐ zero
- ☐ 1-9 per week
- ☐ 10-24 per week
- ☐ 25-49 per week
- ☐ 50+ per week

35. When performing injections, or administrations, was the isotope usually shielded?

- ☐ Yes
- ☐ No

36. During the duration of a procedure, how far from the patient were you usually standing?

- ☐ less than 3 feet
- ☐ 3-9 feet
- ☐ 10 feet or more

37. When performing DIAGNOSTIC RADIOISOTOPE procedures, what percentage of the time did you wear a protective apron?

- ☐ zero
- ☐ less than 25%
- ☐ 25-74%
- ☐ 75% or more

INTERNAL THERAPEUTIC RADIOISOTOPES
(such as radium, cesium 137, iridium 192, Cobalt-60 or radioactive iodine, etc.)

38. How many times did you perform, administer or assist with INTERNAL THERAPEUTIC RADIOISOTOPE procedures <u>during a typical week at this longest job in the 1950s</u>?

- ☐ zero **[GO TO 43]**
- ☐ 1 time per week
- ☐ 2 times per week
- ☐ 3 times per week
- ☐ more than 3 times per week

39. When performing INTERNAL THERAPEUTIC RADIOISOTOPE procedures, what percentage of the time did you wear a protective apron?

- ☐ zero
- ☐ less than 25%
- ☐ 25-74%
- ☐ 75% or more

40. When performing INTERNAL THERAPEUTIC RADIOISOTOPE procedures, what percentage of the time did you stand behind a shield or use a protective shield around the radioactive source?

- ☐ zero
- ☐ less than 25%
- ☐ 25-74%
- ☐ 75% or more

41. What percentage of the time were procedures done using afterloading *(that is, when the radioactive source was remotely withdrawn from the safe and introduced into the patient through cables, etc.)***?**

- ☐ zero
- ☐ less than 25%
- ☐ 25-74%
- ☐ 75% or more

42. What percentage of the time were you responsible for maintaining the radioisotope sources used for implants in the safe, or transporting them to the patient?

- ☐ zero
- ☐ less than 25%
- ☐ 25-74%
- ☐ 75% or more

EXTERNAL RADIATION THERAPY
(such as a betatron, Cobalt-60 teletherapy, Orthovoltage machine or linear accelerator, deep therapy, etc.)

43. How many EXTERNAL BEAM THERAPY procedures did you perform or assist with <u>during a typical week at this longest job in the 1950s</u>?

- ☐ zero
- ☐ less than 25 per week
- ☐ 25-49 per week
- ☐ 50 or more per week

44. Did you work as a radiologic technologist between 1960 through 1969?
- ☐ Yes
- ☐ No → **[GO TO 68 ON PAGE 12]**

The following questions are about *some types* of radiologic procedures you may have worked or assisted with <u>on the job you held the longest between 1960 and 1969</u>. You don't need to know exact answers. Just choose the answers that best fit your experience.

ROUTINE DIAGNOSTIC X-RAY
(such as chest x-rays, portable x-rays, mammograms, etc.)

45. How many ROUTINE DIAGNOSTIC X-RAY procedures did you perform or assist with <u>during a typical week at this longest job in the 1960s</u>?
- ☐ zero **[GO TO 51]**
- ☐ less than 10 per week
- ☐ 10-24 per week
- ☐ 25-49 per week
- ☐ 50-99 per week
- ☐ 100 or more per week

46. When performing ROUTINE DIAGNOSTIC X-RAY procedures, what percentage of the time were you <u>inside</u> the room?
[*Inside* the room includes standing behind a shield or partial wall open to the patient. *Outside* means separated from the patient by the room walls and a door.]
- ☐ zero **[GO TO 51]**
- ☐ less than 25%
- ☐ 25-74%
- ☐ 75% or more

47. When performing ROUTINE DIAGNOSTIC X-RAY procedures, what percentage of the time did you wear a protective apron?
- ☐ zero
- ☐ less than 25%
- ☐ 25-74%
- ☐ 75% or more

48. When performing ROUTINE DIAGNOSTIC X-RAY procedures, what percentage of the time did you stand behind a screen, shield or other protective enclosure?
- ☐ zero **[GO TO 50]**
- ☐ less than 25%
- ☐ 25-74%
- ☐ 75% or more

49. How high did the screen, shield or other protective enclosure extend?
- ☐ above the head
- ☐ up to the shoulders
- ☐ up to the waist

50. When performing ROUTINE DIAGNOSTIC X-RAY procedures, what percentage of patients being x-rayed did you hold during the procedure?
- ☐ zero
- ☐ less than 10%
- ☐ 10-24%
- ☐ 25% or more

FLUOROSCOPY
(such as angiograms, barium swallows, etc.)

51. How many FLUOROSCOPY procedures did you perform or assist with <u>during a typical week at this longest job in the 1960s</u>?
- ☐ zero **[GO TO 57]**
- ☐ less than 10 per week
- ☐ 10-24 per week
- ☐ 25-49 per week
- ☐ 50-99 per week
- ☐ 100 or more per week

52. When performing FLUOROSCOPY procedures, what percentage of the time were you <u>inside</u> the room?
- ☐ zero **[GO TO 56]**
- ☐ less than 25%
- ☐ 25-74%
- ☐ 75% or more

53. When performing FLUOROSCOPY procedures, what percentage of the time did you wear a protective apron?
- ☐ zero
- ☐ less than 25%
- ☐ 25-74%
- ☐ 75% or more

54. When performing FLUOROSCOPY procedures, what percentage of the time did you stand behind a screen, shield or other protective enclosure?
- ☐ zero **[GO TO 56]**
- ☐ less than 25%
- ☐ 25-74%
- ☐ 75% or more

55. How high did the screen, shield or other protective enclosure extend?
- ☐ above the head
- ☐ up to the shoulders
- ☐ up to the waist

56. Excluding set-up time, what percentage of the FLUOROSCOPY procedures that you performed or assisted with were more than 15 minutes long?
- ☐ zero
- ☐ less than 25%
- ☐ 25-49%
- ☐ 50% or more

DIAGNOSTIC RADIOISOTOPE
(such as using I-131 or technecium-99M, etc.)

57. How many DIAGNOSTIC RADIOISOTOPE procedures (injections or oral procedures) did you perform or assist with during a typical week at this longest job in the 1960s?

- ☐ zero **[GO TO 62]**
- ☐ less than 10 per week
- ☐ 10-24 per week
- ☐ 25-49 per week
- ☐ 50-99 per week
- ☐ 100 or more per week

58. When working with DIAGNOSTIC RADIOISOTOPES, how many times did you prepare the radiopharmaceutical kit and/or elute generators *(i.e., separate and measure a concentrated amount ofisotope from its source for use in a radiopharmaceutical kit)* **during a typical week at this longest job?**

- ☐ zero
- ☐ 1-9 per week
- ☐ 10-24 per week
- ☐ 25-49 per week
- ☐ 50+ per week

59. When performing injections, or administrations, was the isotope usually shielded?

- ☐ Yes
- ☐ No

60. During the duration of a procedure, how far from the patient were you usually standing?

- ☐ less than 3 feet
- ☐ 3-9 feet
- ☐ 10 feet or more

61. When performing DIAGNOSTIC RADIOISOTOPE procedures, what percentage of the time did you wear a protective apron?

- ☐ zero
- ☐ less than 25%
- ☐ 25-74%
- ☐ 75% or more

INTERNAL THERAPEUTIC RADIOISOTOPES
(such as radium, cesium 137, iridium 192, Cobalt-60 or radioactive iodine, etc.)

62. How many times did you perform, administer or assist with INTERNAL THERAPEUTIC RADIOISOTOPE procedures during a typical week at this longest job in the 1960s?

- ☐ zero **[GO TO 67]**
- ☐ 1 time per week
- ☐ 2 times per week
- ☐ 3 times per week
- ☐ more than 3 times per week

63. When performing INTERNAL THERAPEUTIC RADIOISOTOPE procedures, what percentage of the time did you wear a protective apron?

- ☐ zero
- ☐ less than 25%
- ☐ 25-74%
- ☐ 75% or more

64. When performing INTERNAL THERAPEUTIC RADIOISOTOPE procedures, what percentage of the time did you stand behind a shield or use a protective shield around the radioactive source?

- ☐ zero
- ☐ less than 25%
- ☐ 25-74%
- ☐ 75% or more

65. What percentage of the time were procedures done using afterloading *(that is, when the radioactive source was remotely withdrawn from the safe and introduced into the patient through cables, etc.)***?**

- ☐ zero
- ☐ less than 25%
- ☐ 25-74%
- ☐ 75% or more

66. What percentage of the time were you responsible for maintaining the radioisotope sources used for implants in the safe, or transporting them to the patient?

- ☐ zero
- ☐ less than 25%
- ☐ 25-74%
- ☐ 75% or more

EXTERNAL RADIATION THERAPY
(such as a betatron, Cobalt-60 teletherapy, Orthovoltage machine or linear accelerator, deep therapy, etc.)

67. How many EXTERNAL BEAM THERAPY procedures did you perform or assist with during a typical week at this longest job in the 1960s?

- ☐ zero
- ☐ less than 25 per week
- ☐ 25-49 per week
- ☐ 50 or more per week

68. **Did you work as a radiologic technologist between 1970 through 1979?**
- ☐ Yes
- ☐ No → **[GO TO 92 ON PAGE 14]**

The following questions are about *some types* of radiologic procedures you may have worked or assisted with on the job you held the longest between 1970 and 1979. You don't need to know exact answers. Just choose the answers that best fit your experience.

ROUTINE DIAGNOSTIC X-RAY
(such as chest x-rays, portable x-rays, mammograms, etc.)

69. **How many ROUTINE DIAGNOSTIC X-RAY procedures did you perform or assist with during a typical week at this longest job in the 1970s?**
- ☐ zero **[GO TO 75]**
- ☐ less than 10 per week
- ☐ 10-24 per week
- ☐ 25-49 per week
- ☐ 50-99 per week
- ☐ 100 or more per week

70. **When performing ROUTINE DIAGNOSTIC X-RAY procedures, what percentage of the time were you inside the room?**
[*Inside* the room includes standing behind a shield or partial wall open to the patient. *Outside* means separated from the patient by the room walls and a door.]
- ☐ zero **[GO TO 75]**
- ☐ less than 25%
- ☐ 25-74%
- ☐ 75% or more

71. **When performing ROUTINE DIAGNOSTIC X-RAY procedures, what percentage of the time did you wear a protective apron?**
- ☐ zero
- ☐ less than 25%
- ☐ 25-74%
- ☐ 75% or more

72. **When performing ROUTINE DIAGNOSTIC X-RAY procedures, what percentage of the time did you stand behind a screen, shield or other protective enclosure?**
- ☐ zero **[GO TO 74]**
- ☐ less than 25%
- ☐ 25-74%
- ☐ 75% or more

73. **How high did the screen, shield or other protective enclosure extend?**
- ☐ above the head
- ☐ up to the shoulders
- ☐ up to the waist

74. **When performing ROUTINE DIAGNOSTIC X-RAY procedures, what percentage of patients being x-rayed did you hold during the procedure?**
- ☐ zero
- ☐ less than 10%
- ☐ 10-24%
- ☐ 25% or more

FLUOROSCOPY
(such as angiograms, barium swallows, etc.)

75. **How many FLUOROSCOPY procedures did you perform or assist with during a typical week at this longest job in the 1970s?**
- ☐ zero **[GO TO 81]**
- ☐ less than 10 per week
- ☐ 10-24 per week
- ☐ 25-49 per week
- ☐ 50-99 per week
- ☐ 100 or more per week

76. **When performing FLUOROSCOPY procedures, what percentage of the time were you inside the room?**
- ☐ zero **[GO TO 80]**
- ☐ less than 25%
- ☐ 25-74%
- ☐ 75% or more

77. **When performing FLUOROSCOPY procedures, what percentage of the time did you wear a protective apron?**
- ☐ zero
- ☐ less than 25%
- ☐ 25-74%
- ☐ 75% or more

78. **When performing FLUOROSCOPY procedures, what percentage of the time did you stand behind a screen, shield or other protective enclosure?**
- ☐ zero **[GO TO 80]**
- ☐ less than 25%
- ☐ 25-74%
- ☐ 75% or more

79. **How high did the screen, shield or other protective enclosure extend?**
- ☐ above the head
- ☐ up to the shoulders
- ☐ up to the waist

80. **Excluding set-up time, what percentage of the FLUOROSCOPY procedures that you performed or assisted with were more than 15 minutes long?**
- ☐ zero
- ☐ less than 25%
- ☐ 25-49%
- ☐ 50% or more

DIAGNOSTIC RADIOISOTOPE
(such as using I-131 or technecium-99M, etc.)

81. How many DIAGNOSTIC RADIOISOTOPE procedures (injections or oral procedures) did you perform or assist with <u>during a typical week at this longest job in the 1970s</u>?

- ☐ zero **[GO TO 86]**
- ☐ less than 10 per week
- ☐ 10-24 per week
- ☐ 25-49 per week
- ☐ 50-99 per week
- ☐ 100 or more per week

82. When working with DIAGNOSTIC RADIOISOTOPES, how many times did you prepare the radiopharmaceutical kit and/or elute generators *(i.e., separate and measure a concentrated amount ofisotope from its source for use in a radiopharmaceutical kit)* **<u>during a typical week at this longest job</u>?**

- ☐ zero
- ☐ 1-9 per week
- ☐ 10-24 per week
- ☐ 25-49 per week
- ☐ 50+ per week

83. When performing injections, or administrations, was the isotope usually shielded?

- ☐ Yes
- ☐ No

84. During the duration of a procedure, how far from the patient were you usually standing?

- ☐ less than 3 feet
- ☐ 3-9 feet
- ☐ 10 feet or more

85. When performing DIAGNOSTIC RADIOISOTOPE procedures, what percentage of the time did you wear a protective apron?

- ☐ zero
- ☐ less than 25%
- ☐ 25-74%
- ☐ 75% or more

INTERNAL THERAPEUTIC RADIOISOTOPES
(such as radium, cesium 137, iridium 192, Cobalt-60 or radioactive iodine, etc.)

86. How many times did you perform, administer or assist with INTERNAL THERAPEUTIC RADIOISOTOPE procedures <u>during a typical week at this longest job in the 1970s</u>?

- ☐ zero **[GO TO 91]**
- ☐ 1 time per week
- ☐ 2 times per week
- ☐ 3 times per week
- ☐ more than 3 times per week

87. When performing INTERNAL THERAPEUTIC RADIOISOTOPE procedures, what percentage of the time did you wear a protective apron?

- ☐ zero
- ☐ less than 25%
- ☐ 25-74%
- ☐ 75% or more

88. When performing INTERNAL THERAPEUTIC RADIOISOTOPE procedures, what percentage of the time did you stand behind a shield or use a protective shield around the radioactive source?

- ☐ zero
- ☐ less than 25%
- ☐ 25-74%
- ☐ 75% or more

89. What percentage of the time were procedures done using afterloading *(that is, when the radioactive source was remotely withdrawn from the safe and introduced into the patient through cables, etc.)***?**

- ☐ zero
- ☐ less than 25%
- ☐ 25-74%
- ☐ 75% or more

90. What percentage of the time were you responsible for maintaining the radioisotope sources used for implants in the safe, or transporting them to the patient?

- ☐ zero
- ☐ less than 25%
- ☐ 25-74%
- ☐ 75% or more

EXTERNAL RADIATION THERAPY
(such as a betatron, Cobalt-60 teletherapy, Orthovoltage machine or linear accelerator, deep therapy, etc.)

91. How many EXTERNAL BEAM THERAPY procedures did you perform or assist with <u>during a typical week at this longest job in the 1970s</u>?

- ☐ zero
- ☐ less than 25 per week
- ☐ 25-49 per week
- ☐ 50 or more per week

92. Did you work as a radiologic technologist after 1980 through the present?

- ☐ Yes
- ☐ No → **[GO TO 116 ON PAGE 16]**

The following questions are about *some types* of radiologic procedures you may have worked or assisted with <u>on the job you held the longest from 1980 through the present</u>. You don't need to know exact answers. Just choose the answers that best fit your experience.

ROUTINE DIAGNOSTIC X-RAY
(such as chest x-rays, portable x-rays, mammograms, etc.)

93. How many ROUTINE DIAGNOSTIC X-RAY procedures did you perform or assist with <u>during a typical week at this longest job from 1980 to the present</u>?

- ☐ zero **[GO TO 99]**
- ☐ less than 10 per week
- ☐ 10-24 per week
- ☐ 25-49 per week
- ☐ 50-99 per week
- ☐ 100 or more per week

94. When performing ROUTINE DIAGNOSTIC X-RAY procedures, what percentage of the time were you <u>inside</u> the room?
[*Inside* the room includes standing behind a shield or partial wall open to the patient. *Outside* means separated from the patient by the room walls and a door.]

- ☐ zero **[GO TO 99]**
- ☐ less than 25%
- ☐ 25-74%
- ☐ 75% or more

95. When performing ROUTINE DIAGNOSTIC X-RAY procedures, what percentage of the time did you wear a protective apron?

- ☐ zero
- ☐ less than 25%
- ☐ 25-74%
- ☐ 75% or more

96. When performing ROUTINE DIAGNOSTIC X-RAY procedures, what percentage of the time did you stand behind a screen, shield or other protective enclosure?

- ☐ zero **[GO TO 98]**
- ☐ less than 25%
- ☐ 25-74%
- ☐ 75% or more

97. How high did the screen, shield or other protective enclosure extend?

- ☐ above the head
- ☐ up to the shoulders
- ☐ up to the waist

98. When performing ROUTINE DIAGNOSTIC X-RAY procedures, what percentage of patients being x-rayed did you hold during the procedure?

- ☐ zero
- ☐ less than 10%
- ☐ 10-24%
- ☐ 25% or more

FLUOROSCOPY
(such as angiograms, barium swallows, etc.)

99. How many FLUOROSCOPY procedures did you perform or assist with <u>during a typical week at this longest job from 1980 to the present</u>?

- ☐ zero **[GO TO 105]**
- ☐ less than 10 per week
- ☐ 10-24 per week
- ☐ 25-49 per week
- ☐ 50-99 per week
- ☐ 100 or more per week

100. When performing FLUOROSCOPY procedures, what percentage of the time were you <u>inside</u> the room?

- ☐ zero **[GO TO 104]**
- ☐ less than 25%
- ☐ 25-74%
- ☐ 75% or more

101. When performing FLUOROSCOPY procedures, what percentage of the time did you wear a protective apron?

- ☐ zero
- ☐ less than 25%
- ☐ 25-74%
- ☐ 75% or more

102. When performing FLUOROSCOPY procedures, what percentage of the time did you stand behind a screen, shield or other protective enclosure?

- ☐ zero **[GO TO 104]**
- ☐ less than 25%
- ☐ 25-74%
- ☐ 75% or more

103. How high did the screen, shield or other protective enclosure extend?

- ☐ above the head
- ☐ up to the shoulders
- ☐ up to the waist

104. Excluding set-up time, what percentage of the FLUOROSCOPY procedures that you performed or assisted with were more than 15 minutes long?

- ☐ zero
- ☐ less than 25%
- ☐ 25-49%
- ☐ 50% or more

DIAGNOSTIC RADIOISOTOPE
(such as using I-131 or technecium-99M, etc.)

105. How many DIAGNOSTIC RADIOISOTOPE procedures (injections or oral procedures) did you perform or assist with <u>during a typical week at this longest job from 1980 to the present</u>?

- ☒ zero **[GO TO 110]**
- ☒ less than 10 per week
- ☒ 10-24 per week
- ☒ 25-49 per week
- ☒ 50-99 per week
- ☒ 100 or more per week

106. When working with DIAGNOSTIC RADIOISOTOPES, how many times did you prepare the radiopharmaceutical kit and/or elute generators *(i.e., separate and measure a concentrated amount ofisotope from its source for use in a radiopharmaceutical kit)* **<u>during a typical week at this longest job</u>?**

- ☒ zero
- ☒ 1-9 per week
- ☒ 10-24 per week
- ☒ 25-49 per week
- ☒ 50+ per week

107. When performing injections, or administrations, was the isotope usually shielded?

- ☒ Yes
- ☒ No

108. During the duration of a procedure, how far from the patient were you usually standing?

- ☒ less than 3 feet
- ☒ 3-9 feet
- ☒ 10 feet or more

109. When performing DIAGNOSTIC RADIOISOTOPE procedures, what percentage of the time did you wear a protective apron?

- ☒ zero
- ☒ less than 25%
- ☒ 25-74%
- ☒ 75% or more

INTERNAL THERAPEUTIC RADIOISOTOPES
(such as radium, cesium 137, iridium 192, Cobalt-60 or radioactive iodine, etc.)

110. How many times did you perform, administer or assist with INTERNAL THERAPEUTIC RADIOISOTOPE procedures <u>during a typical week at this longest job from 1980 to the present</u>?

- ☒ zero **[GO TO 115]**
- ☒ 1 time per week
- ☒ 2 times per week
- ☒ 3 times per week
- ☒ more than 3 times per week

111. When performing INTERNAL THERAPEUTIC RADIOISOTOPE procedures, what percentage of the time did you wear a protective apron?

- ☒ zero
- ☒ less than 25%
- ☒ 25-74%
- ☒ 75% or more

112. When performing INTERNAL THERAPEUTIC RADIOISOTOPE procedures, what percentage of the time did you stand behind a shield or use a protective shield around the radioactive source?

- ☒ zero
- ☒ less than 25%
- ☒ 25-74%
- ☒ 75% or more

113. What percentage of the time were procedures done using afterloading *(that is, when the radioactive source was remotely withdrawn from the safe and introduced into the patient through cables, etc.)***?**

- ☒ zero
- ☒ less than 25%
- ☒ 25-74%
- ☒ 75% or more

114. What percentage of the time were you responsible for maintaining the radioisotope sources used for implants in the safe, or transporting them to the patient?

- ☒ zero
- ☒ less than 25%
- ☒ 25-74%
- ☒ 75% or more

EXTERNAL RADIATION THERAPY
(such as a betatron, Cobalt-60 teletherapy, Orthovoltage machine or linear accelerator, deep therapy, etc.)

115. How many EXTERNAL BEAM THERAPY procedures did you perform or assist with <u>during a typical week at this longest job from 1980 to the present</u>?

- ☒ zero
- ☒ less than 25 per week
- ☒ 25-49 per week
- ☒ 50 or more per week

Radiologic Technologist Work History - General

The following questions pertain to any job you may have held as a radiologic technologist.

116. As a radiologic technologist, were you ever removed from any job because you had exceeded a radiation protection limit?

☐ Yes ☐ No **[GO TO 119]**

117. If YES, how many times . . . ?

☐ 1 to 2 times
☐ 3 or 4 times
☐ 5 or more times

118. In what year were you first removed from a job for this reason?

Y Y Y Y YEAR

119. While you worked as a radiologic technologist, was your white blood cell count ever found to be below normal due to your work as a radiologic technologist?

☐ Yes ☐ No **[GO TO 122]**
☐ Never tested **[GO TO 122]**

120. If YES, how many times . . . ?

☐ 1 to 2 times
☐ 3 or 4 times
☐ 5 or more times

121. In what year were you first told that your white blood cell count was below normal due to your work as a radiologic technologist?

Y Y Y Y YEAR

122. Did you ever work with radiation in a non-medical job?

☐ Yes ☐ No **[GO TO 126]**

123. What year did you start? Y Y Y Y YR

124. What year did you stop? Y Y Y Y YR

125. Briefly describe the type of business and radiation procedures you performed on this job?

____

____

____

SUN EXPOSURE

The following questions focus on your complexion and your exposure to the sun.

126. What is the natural color of your eyes?

- ☐ Blue
- ☐ Green/blue or green/grey
- ☐ Hazel (light brown or yellow with blue or green flecks)
- ☐ Light brown
- ☐ Dark brown
- ☐ Other: ____

127. What was your natural hair color when you were 20 years old . . .

- ☐ Blonde
- ☐ Red
- ☐ Reddish-brown
- ☐ Light brown
- ☐ Medium brown
- ☐ Dark brown
- ☐ Black
- ☐ Other: ____

128. Do you have a light, medium or dark complexion?

- ☐ Light
- ☐ Medium
- ☐ Dark
- ☐ Other: ____

129. How would your skin react if you had no tan the first time in summer you were exposed to strong sunlight for 30 minutes without protective sunscreen? Strong sunlight means noonday sunlight on the brightest, clearest day in summer. Would you . . .

- ☐ Get a severe sunburn with blisters
- ☐ Get a painful sunburn but no blisters
- ☐ Get a mild sunburn followed by some suntan
- ☐ Become tanned without any sunburn
- ☐ No change in skin color

130. After repeated and prolonged exposure to sunlight, would your skin become . . .

- ☐ Very brown and deeply tanned
- ☐ Moderately tanned
- ☐ Lightly tanned
- ☐ Not tanned at all

131. Were you ever sunburnt so severely as to cause blisters?

☐ Yes ☐ No **[GO TO 134]**

132. How many times did this happen before age 15?

☐☐ # of blistering sunburns

133. How many times did this happen from age 15 to the age you are now?

☐☐ # of blistering sunburns

134. The next questions are about where you lived at different ages and about the amount of time you spent in the sun at those ages. Be sure to include time in the sun **on vacations**, **at work** and **where you lived**. (Leave blank if age group is not applicable to you.)<br><br>**When you were (AGE), where did you live the longest?**		**135. When you were (this AGE), on weekDAYS in the summer (between the hours of 9AM and 3PM), about how many hours per day did you usually spend in strong sunlight?**	**136. When you were (this AGE), on weekENDS in the summer (between the hours of 9AM and 3PM), about how many hours per day did you usually spend in strong sunlight?**
**AGE**			
**a. Under 13 years old**	CITY<br>STATE COUNTRY (If outside USA)	☐ 0<br>☐ less than 1 hr<br>☐ 1-2 hrs<br>☐ 3-4 hrs<br>☐ 5-6 hrs	☐ 0<br>☐ less than 1 hr<br>☐ 1-2 hrs<br>☐ 3-4 hrs<br>☐ 5-6 hrs
**b. 13 to 19 years old**	CITY<br>STATE COUNTRY (If outside USA)	☐ 0<br>☐ less than 1 hr<br>☐ 1-2 hrs<br>☐ 3-4 hrs<br>☐ 5-6 hrs	☐ 0<br>☐ less than 1 hr<br>☐ 1-2 hrs<br>☐ 3-4 hrs<br>☐ 5-6 hrs
**c. 20 to 39 years old**	CITY<br>STATE COUNTRY (If outside USA)	☐ 0<br>☐ less than 1 hr<br>☐ 1-2 hrs<br>☐ 3-4 hrs<br>☐ 5-6 hrs	☐ 0<br>☐ less than 1 hr<br>☐ 1-2 hrs<br>☐ 3-4 hrs<br>☐ 5-6 hrs
**d. 40 to 64 years old**	CITY<br>STATE COUNTRY (If outside USA)	☐ 0<br>☐ less than 1 hr<br>☐ 1-2 hrs<br>☐ 3-4 hrs<br>☐ 5-6 hrs	☐ 0<br>☐ less than 1 hr<br>☐ 1-2 hrs<br>☐ 3-4 hrs<br>☐ 5-6 hrs
**e. Age 65 to present**	CITY<br>STATE COUNTRY (If outside USA)	☐ 0<br>☐ less than 1 hr<br>☐ 1-2 hrs<br>☐ 3-4 hrs<br>☐ 5-6 hrs	☐ 0<br>☐ less than 1 hr<br>☐ 1-2 hrs<br>☐ 3-4 hrs<br>☐ 5-6 hrs

General Information

The final questions are about you and your spouse or partner's education, marital status and income. We're asking these questions to allow us to compare participants in this study with those in other health studies and because these factors may also affect disease risk.

137. What is the <u>highest</u> level of education you have completed?

- ☐ 1-8 years (grade school)
- ☐ 9-12 years (high school)
- ☐ 2-year hospital radiologic technology program
- ☐ 1-4 years college
- ☐ Graduate school
- ☐ Other (e.g. vocational)

138. What is your current marital status?

- ☐ Never married **[GO TO 140]**
- ☐ Married
- ☐ Living together but not married
- ☐ Divorced
- ☐ Widowed
- ☐ Separated

139. What was the <u>highest</u> level of education your (current/former) spouse or partner completed? *If you've had more than one spouse or partner, please answer about the one you were with <u>the longest</u>.*

- ☐ 1-8 years (grade school)
- ☐ 9-12 years (high school)
- ☐ 2-year hospital radiologic technology program
- ☐ 1-4 years college
- ☐ Graduate school
- ☐ Other (e.g. vocational)

140. Which of the categories below best describes your current total annual household income before taxes? *Please include all sources of income for all members of your household.*

- ☐ Less than $25,000
- ☐ $25,000-$49,999
- ☐ $50,000-$74,999
- ☐ $75,000-$99,999
- ☐ $100,000 or more

141. In case we need to contact you, please provide your daytime and/or evening telephone number.

Daytime number:

☐☐☐ – ☐☐☐ – ☐☐☐☐

Area code Phone number

Evening number:

☐☐☐ – ☐☐☐ – ☐☐☐☐

Area code Phone number

142. Would you be willing to answer a future questionnaire over the internet using a secure website?

- ☐ Yes
- ☐ No, not willing
- ☐ No, do not have access to internet

Thank you for taking the time to complete this questionnaire.

If you would like to provide additional comments, please use the back of the survey.

We regret that we are unable to respond to individual questions at this time. If you have questions or concerns about your health, we encourage you to discuss these matters with your personal physician. We welcome your comments about the survey as this information may help us plan for the study in the future.

Do you have any comments about this survey?

What would you like to see in the future newsletters to participants in this study?

USRT **Skin Cancer Follow-up Study**

Study ID:

U.S. Radiologic Technologists Study

A collaborative effort between the University of Minnesota School of Public Health, National Cancer Institute, and American Registry of Radiologic Technologists.

SKIN CANCER MAP

Why study skin cancer in the USRT population?

- Skin cancer is the most common type of cancer.
- It can be caused by both ionizing radiation and ultraviolet (UV) radiation from sunlight.
- The USRT study provides a unique opportunity to study the independent and combined effects of these types of radiation on skin cancer and melanoma, which are also linked to sunlight exposure.
- More than 4,000 new cases of skin cancer and 700 new cases of melanoma were reported on the third survey.

Please take a few minutes to complete the enclosed **Skin Cancer Follow-up Form** and mark the location of each skin cancer on this **Skin Cancer Map**.

A postage paid envelope is enclosed for return of the study materials. If you have any questions, please call the University of Minnesota USRT study office at 1-800-447-6466.

Your participation in this Skin Cancer Follow-up Study is greatly appreciated.

PRIVACY AND CONFIDENTIALITY:
Please be assured that all information provided will be kept private under the Privacy Act, and will not be disclosed to anyone but the researchers conducting this study or as provided by law. To protect your privacy further, we have also obtained a Certificate of Confidentiality as described in the HIPAA form. Published results will be reported in statistical summaries only, and will never include a participant's name. This study is authorized under Section 411 of the Public Health Service Act [42 USC 285a]. Your participation in this study is completely voluntary, and failure to answer any particular questions or the information collection as a whole will not affect your future contacts with the A.R.R.T., any medical facility, or government agency.

Instructions:

You may use a pen or pencil to complete this form.

√ Review the enclosed **USRT Study Skin Cancer Follow-up form** to verify skin cancers you have previously reported. Add any new skin cancers you have had diagnosed since that time.

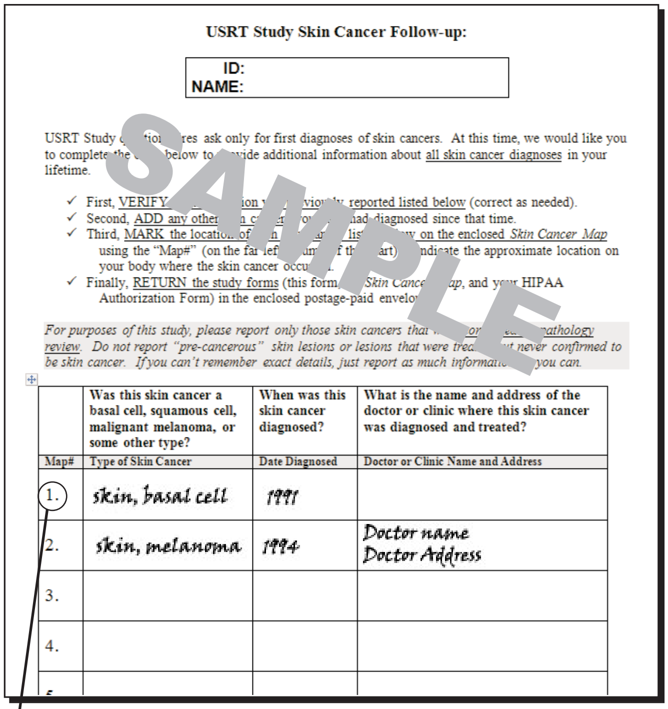

USRT Study Skin Cancer Follow-up:

ID:
NAME:

SAMPLE

USRT Study [illegible] ask only for first diagnoses of skin cancers. At this time, we would like you to complete [illegible] below to [illegible] additional information about all skin cancer diagnoses in your lifetime.

- ✓ First, VERIFY [illegible] reported listed below (correct as needed).
- ✓ Second, ADD any other [illegible] diagnosed since that time.
- ✓ Third, MARK the location [illegible] on the enclosed *Skin Cancer Map* using the "Map#" (on the far [illegible]) [illegible] the approximate location on your body where the skin cancer [illegible]
- ✓ Finally, RETURN the study forms (this form, [illegible] *Skin Cance*[illegible], and your HIPAA Authorization Form) in the enclosed postage-paid envelo[illegible]

For purposes of this study, please report only those skin cancers that [illegible] pathology review. Do not report "pre-cancerous" skin lesions or lesions that were trea[illegible] never confirmed to be skin cancer. If you can't remember exact details, just report as much informatio[illegible] you can.

	Was this skin cancer a basal cell, squamous cell, malignant melanoma, or some other type?	When was this skin cancer diagnosed?	What is the name and address of the doctor or clinic where this skin cancer was diagnosed and treated?
Map#	Type of Skin Cancer	Date Diagnosed	Doctor or Clinic Name and Address
1.	skin, basal cell	1991	
2.	skin, melanoma	1994	Doctor name<br>Doctor Address
3.			
4.			
[illegible]			

√ For each skin cancer listed on the **Skin Cancer Follow-up** Form, MARK the location on this **Skin Cancer Map** form using the Map# number on the **Skin Cancer Follow-up** Form.

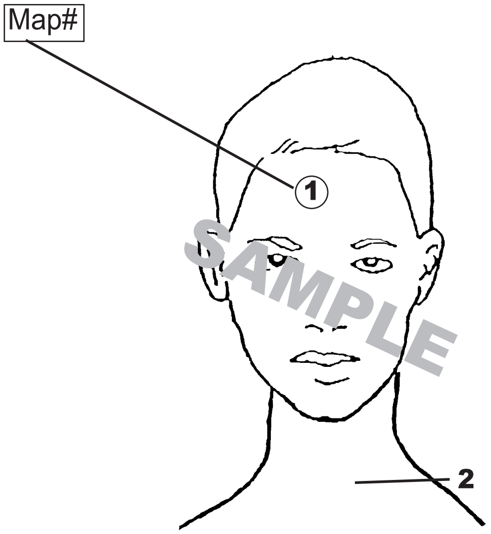

HEAD, FACE, AND NECK
FRONT

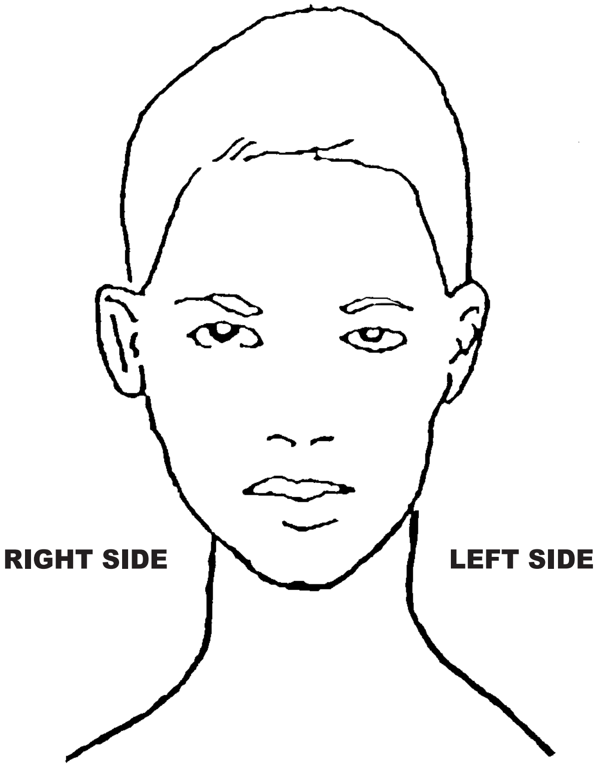

HEAD, FACE, AND NECK
FRONT

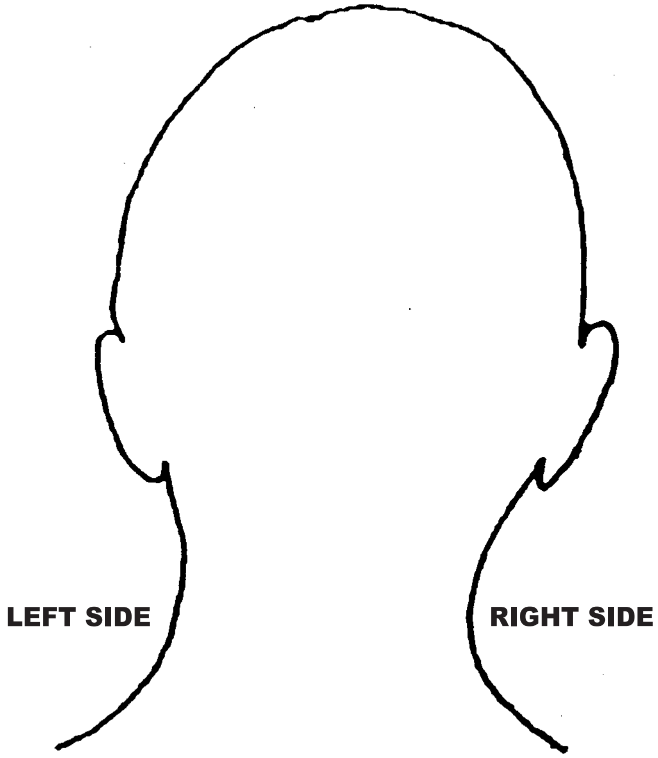

HEAD, FACE, AND NECK
BACK

RIGHT SIDE LEFT SIDE

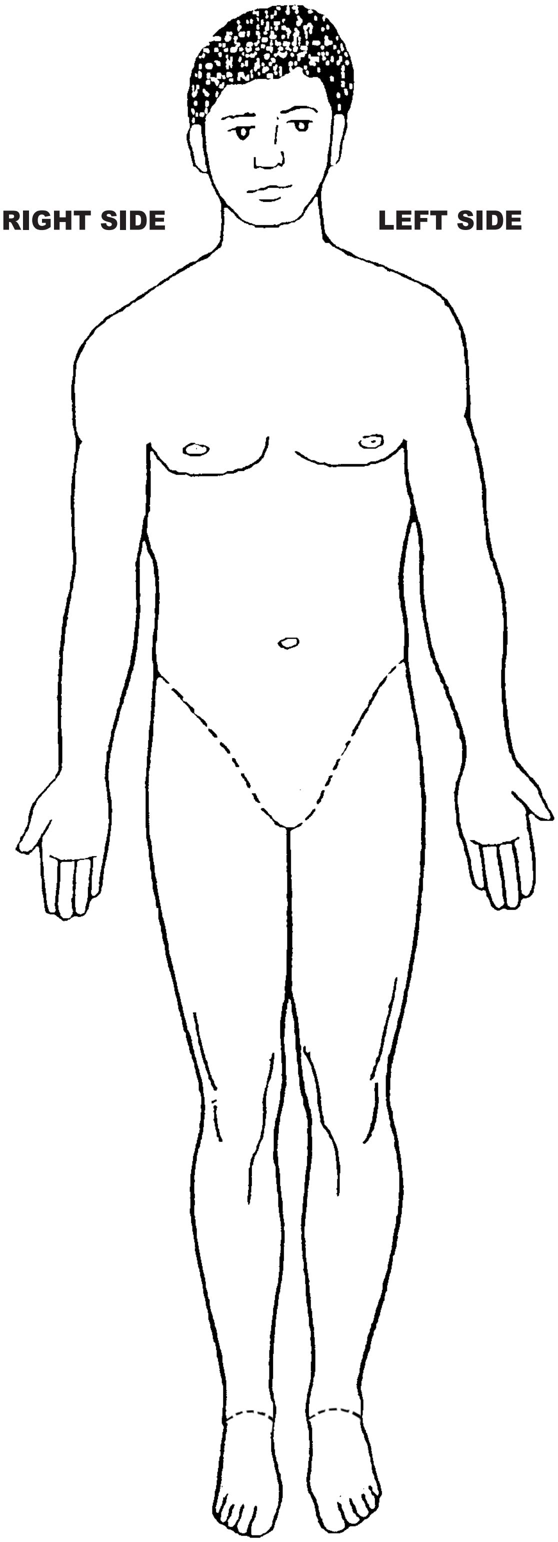

FRONT

LEFT SIDE RIGHT SIDE

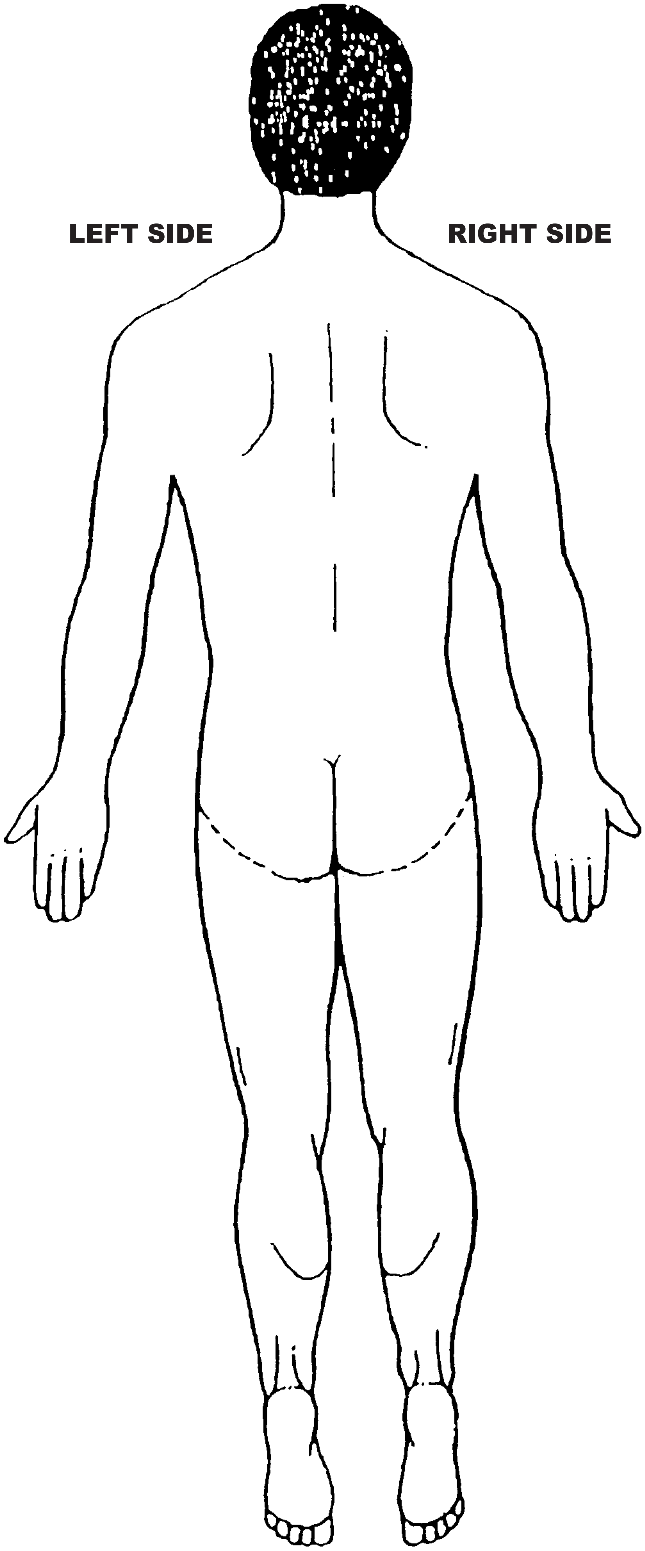

BACK
